# Biased localization of actin binding proteins by actin filament conformation

**DOI:** 10.1038/s41467-020-19768-9

**Published:** 2020-11-25

**Authors:** Andrew R. Harris, Pamela Jreij, Brian Belardi, Aaron M. Joffe, Andreas R. Bausch, Daniel A. Fletcher

**Affiliations:** 1 Department of Bioengineering and Biophysics Program, University of California, Berkeley, 648 Stanley Hall MC 1762, Berkeley, CA 94720 USA; 2 Lehrstuhl für Biophysik (E27), Technische Universität München, Garching, 85748 Germany; 3 Biological Systems and Engineering Division, Lawrence Berkeley National Laboratory, 648 Stanley Hall MC 1762, Berkeley, CA 94720 USA; 4 Chan Zuckerberg Biohub, San Francisco, CA 94158 USA

**Keywords:** Super-resolution microscopy, Kinetics, Single-molecule biophysics, Cell migration, Actin

## Abstract

The assembly of actin filaments into distinct cytoskeletal structures plays a critical role in cell physiology, but how proteins localize differentially to these structures within a shared cytoplasm remains unclear. Here, we show that the actin-binding domains of accessory proteins can be sensitive to filament conformational changes. Using a combination of live cell imaging and in vitro single molecule binding measurements, we show that tandem calponin homology domains (CH1–CH2) can be mutated to preferentially bind actin networks at the front or rear of motile cells. We demonstrate that the binding kinetics of CH1–CH2 domain mutants varies as actin filament conformation is altered by perturbations that include stabilizing drugs and other binding proteins. These findings suggest that conformational changes of actin filaments in cells could help to direct accessory binding proteins to different actin cytoskeletal structures through a biophysical feedback loop.

## Abstract (web summary)

The assembly of actin filaments into distinct cytoskeletal structures plays a critical role in cell physiology. Here, the authors use a combination of live cell imaging and in vitro single molecule binding measurements to show that tandem calponin homology domains (CH1–CH2) are sensitive to actin filament conformation, biasing their subcellular localization.

## Introduction

Multiple actin cytoskeletal structures co-exist within the cytoplasm, yet they are spatially organized, architecturally distinct, and perform specific functions^1,2^. In addition to branched actin networks in the lamellipodium, and stress fibers in the cell body, advances in both optical and electron microscopy continue to reveal more details about the organization and assembly of a broader range of actin structures, including filopodia^3^, asters and stars^4^, podosomes^5^ and patches^6^. In each of these structures, the interaction of actin filaments with a vast set of accessory proteins promotes the formation of distinct cytoskeletal architectures.

Interestingly, common probes for F-actin, including GFP-tagged actin, small actin-binding peptides (lifeact^7^, f-tractin^8^, affimers^9^) and phallotoxins^10^, are known to not distribute evenly on different actin cytoskeletal structures^10–13^. Similar observations have been made for fluorescent fusions to minimal actin-binding domains from different proteins^14–17^. Mechanistically, these results have been attributed to the complex and competitive interactions between side binding proteins^18,19^, the effect of actin nucleators^20,21^, and the kinetic properties of the reporting probe^10,22^. However, other properties of an actin filament, including its conformational state, could differ among cytoskeletal structures and be detected by actin-binding proteins to bias their localization.

Several studies have indicated that the conformational state of an actin filament is polymorphic^23–25^ and that actin filaments exist in a range of different states, including different nucleotide state^26^, oxidative state^27^ and twisted states^23,28,29^. Actin-binding proteins have also been shown to modulate filament structural conformations either as part of their regulatory activity or as a means for allosteric cooperative binding to actin^30–32^. In addition to effects of protein binding, mechanical perturbations to actin filaments such as torques, tension^33,34^ and bending have been suggested to influence protein interactions with filaments, including the binding activity of the Arp2/3 complex^21^ and severing activity of the protein cofilin^20,35,36^. Together, these observations suggest that different conformations of F-actin, induced either mechanically or biochemically, could impact the affinity of actin-binding proteins for F-actin.

We investigated whether filament conformational changes could be sensed by a common class of actin-binding domain, tandem calponin homology domains (CH1–CH2), and if differences in affinity for F-actin conformations could potentially influence the localization of CH1–CH2-containing proteins to different actin structures in cells. Using a combination of live cell imaging and in vitro single molecule binding kinetics measurements, we find that mutants of the actin-binding domain of utrophin (CH1–CH2) localize to different actin structures and exhibit different binding kinetics on actin filaments whose conformational state has been altered biophysically and biochemically. We also show that this mechanism potentially extends to native actin-binding domains, suggesting that sensitivity to actin filament conformational states could be playing an important role in the organization and regulation of actin-binding proteins in actin filament structures.

## Results

### Two mechanisms regulate CH1–CH2 binding to F-actin

CH1–CH2 domains are found in many actin crosslinking and regulatory proteins^37^, including α-actinins in stress fibers^38^ and filamins in the actin cortex^39^. The minimal actin-binding domain of utrophin (CH1–CH2) is often used as a generic marker for F-actin^40^, which raises the question of whether it uniformly labels all filaments in cells or might be biased towards a specific actin filament conformation.

**Fig. 1:**
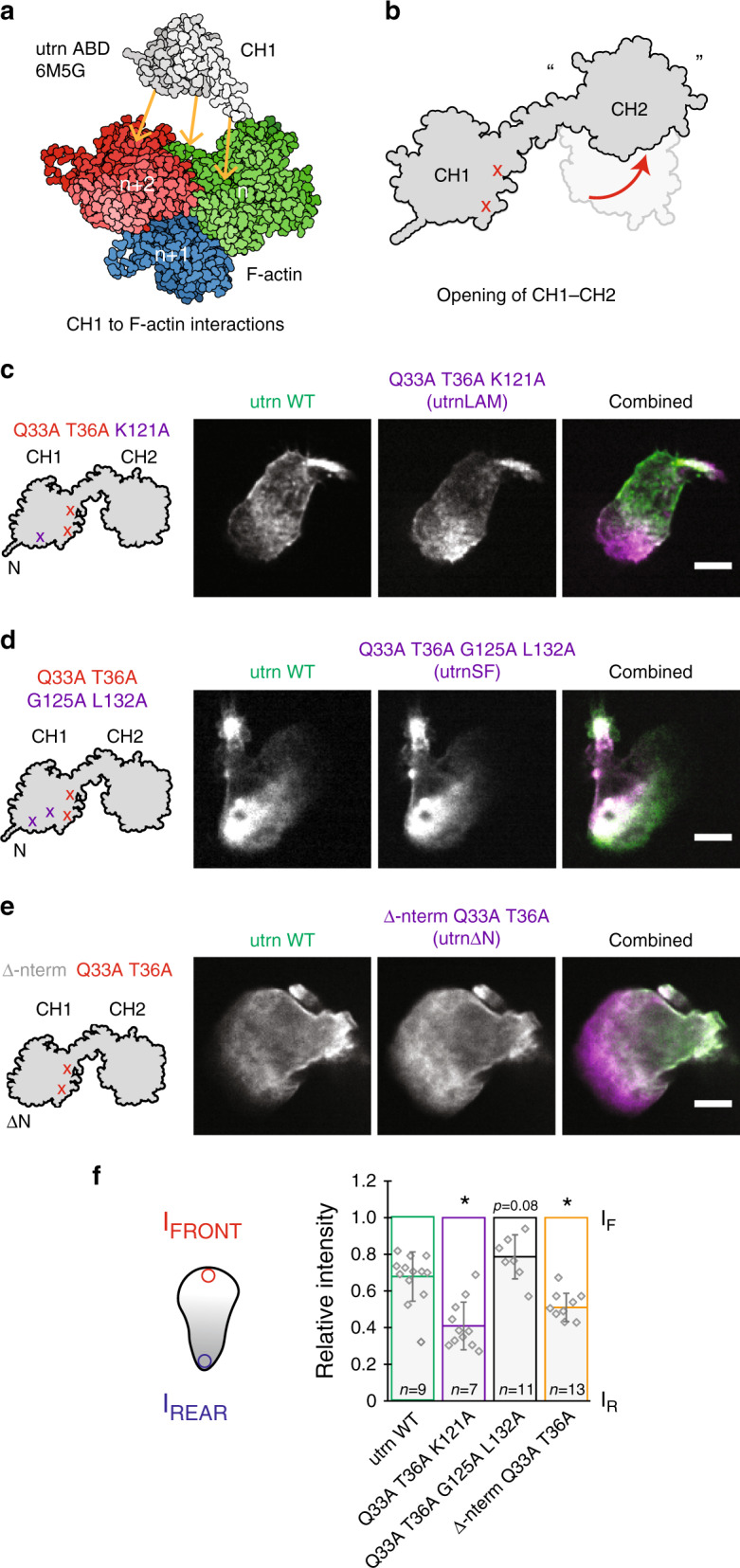
utrn CH1–CH2 mutants display differential localization in neutrophils. **a** Representation of the actin-binding domain of utrophin binding to actin (6M5G^42^) which makes contact on and in between adjacent monomers on an actin filament. CH1 only is shown in this graphic. **b** Mutations to residues at the interface between CH1 and CH2 change the ability of CH1–CH2 domains to adopt an open conformation, which relieves a steric interaction between CH2 and F-actin and results in an increase in binding affinity^37^. **c** The mutant utrn Q33A T36A K121A is localized more strongly to the leading edge than utrnWT. **d** The mutant utrn Q33A T36A G125A L132A is localized more strongly to the rear of the cell than utrnWT. **e** The mutant utrn Δ-nterm Q33A T36A is localized more evenly distributed at the front and back of the cell than utrnWT. Scale bars are 5 µm. **f** Comparisons of the relative utrn construct intensity at the front and back of migrating neutrophils, calculated by averaging the intensity in 1 µm regions at the front and back of the cell (left). Conditions were compared using a two-tailed students t-test and assumed significant at *p < 0.05.

Two mechanisms govern the overall binding affinity of CH1–CH2 domains with F-actin. Firstly, residues on CH1 make direct interactions with F-actin. Recent cryo-electron microscopy studies have mapped the interacting residues between the actin-binding domain from filamin A and F-actin^41^, and between the actin-binding domain of utrophin and F-actin to 3.6 Å resolution^42^. These studies identified three major regions on CH1 directly interact with F-actin, ABS-N, ABS2 and ABS2’, which make contact with two longitudinally adjacent subunits in an actin filament^41,42^ (Fig. 1a). Binding two adjacent actin subunits could potentially serve as a mechanism for sensing small changes in actin filament conformation such as filament twist. Secondly, CH2 acts as a negative regulator of F-actin binding affinity, by sterically clashing with the actin filament. Our previous work^37^, and the work of others^41,43,44^, has shown that mutations targeting residues involved in CH1–CH2 interactions can promote opening of the tandem domain (for example, Q33A T36A on utrnWT). This relieves the steric interactions between CH2 and F-actin and thus increases binding affinity (Fig. 1b).

These two mechanisms for modulating affinity – changing steric interference by CH2 and changing the binding surface of CH1 to f-actin—are likely to be independent for many residues in the domain. By combining CH1–CH2 interface mutations, which increase F-actin binding affinity, with mutations targeting CH1–F-actin interactions, which decrease F-actin binding affinity and potentially change conformational sensing, we hypothesized that it would be possible to generate combinations of mutations that retained a similar bulk binding affinity to F-actin but exhibited preferences for specific F-actin conformations. To test this idea, we chose to generate mutants targeting residues predicted to lie within actin binding surface 2 (ABS2) on utrophin CH1^41,42^, at the CH1–CH2 domain interface^37^ and within ABS-N (also referred to as the N-terminal flanking region)^37,44,45^.

### Utrophin actin-binding domain mutants localize to different actin structures

We first compared the localization of a range of mutants of the actin-binding domain of utrophin (utrnWT) in both HeLa cells (Fig. S1) and PLB neutrophils (Fig. 1), which exhibit distinct actin structures. Interestingly, we observed several combinations of mutations that caused significant changes in localization relative to utrnWT. The mutations Q33A T36A K121A caused an increased enrichment towards lamellipodial actin (Fig. 1c and Supplementary Movie S1, Supplementary Movie S2), while Q33A T36A G125A L132A was comparatively enriched at the rear of the cell (Fig. 1d). We have previously shown that truncating the N-terminal flanking region, Δ-nterm Q33A T36A, changes binding to focal adhesions in HeLa cells^37^ (Supplementary Movie S3), and this mutant was more evenly distributed at the rear and front of migrating neutrophils compared to utrnWT (Fig. 1e and Supplementary Movie S4). Subsequently, we refer to the minimal actin-binding domain of utrophin as utrnWT, Q33A T36A K121A mutant as utrnLAM, the Δ-nterm Q33A T36A mutant as utrnΔN, and the Q33A T36A G125A L132A mutant as utrnSF (Fig. 1f).

### Single molecule kinetic measurements show how mutations combine to determine affinity and specificity

**Fig. 2:**
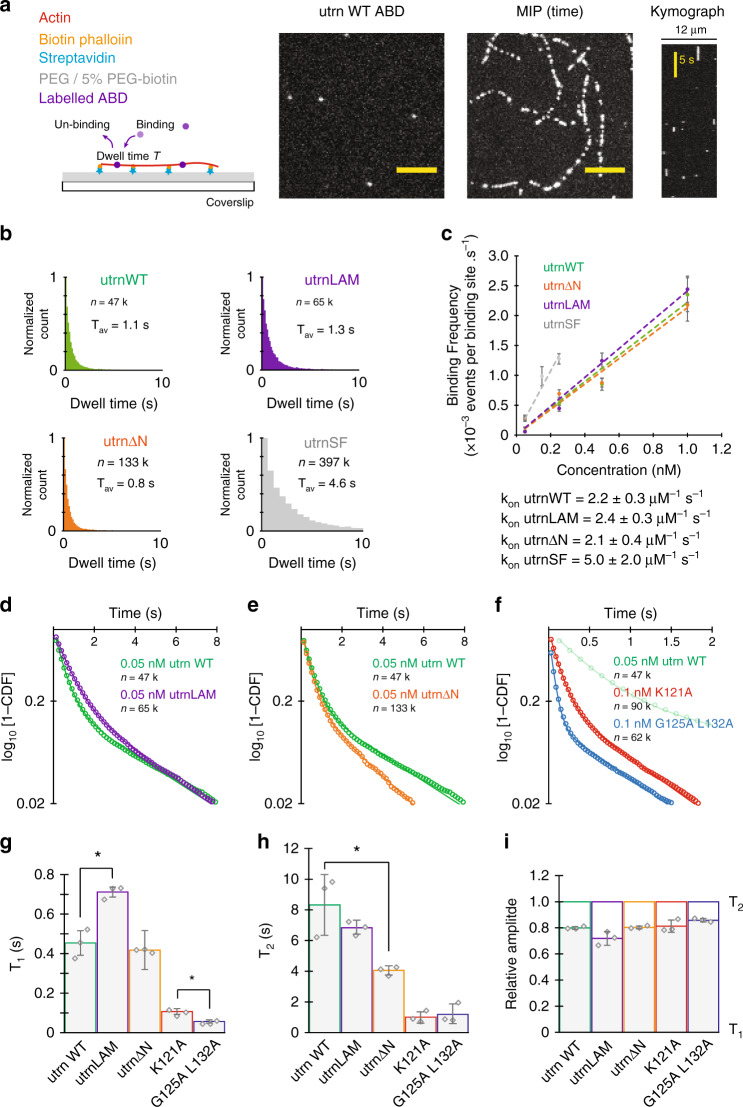
Single molecule kinetic measurements of utrn CH1–CH2 mutants in vitro. **a** Single molecule binding assay to measure the kinetic properties. Images in the example shown are for utrnWT. Maximum intensity projection through time displays the filament backbones and a kymograph the kinetics of binding. Scale bars are 5 µm. The kymograph is a 12 µm section of filament backbone. **b** Average binding dwell times for the different CH1–CH2 mutants. **c** Binding on-rates for the different utrophin mutants evaluated by fitting the binding event frequency over a range of different concentrations. Error bars are the mean ± SEM for each concentration measured from more than 12 fields of view collected from two imaging chambers. **d** Cumulative distribution function for utrnWT (green) and utrnLAM (magenta). **e** Cumulative distribution function for utrnWT (green) and utrnΔN (orange). **f** Cumulative distribution function for K121A (red) and G125A L132A (blue). **g** Comparisons of the first timescale τ_1_, **h** second timescale τ_2_, and **i** relative amplitude of events belonging to each timescale from a double exponential fit to the cumulative distribution functions for the different constructs. Error bars are the standard deviation from the mean of 3 technical replicates. Conditions were compared using a two-tailed students *t*-test, with *p* < 0.05 denoted by a star (*).

We wondered whether the differences in localization of these domains could be influenced by a bias in binding affinity for actin filaments in each specific network – which we refer to as specificity. To investigate this, we characterised the binding properties of each mutant in more detail in vitro (Fig. S2). Previously, single molecule kinetic measurements have been used to investigate the binding properties of actin severing proteins^46^, formins^47^, cofilin^48^ and the actin-binding domain of α-catenin^49^. For α-catenin, the binding dwell time of single molecules (inverse of the off-rate) was shown to follow a two-timescale binding behaviour, in which the binding dwell times increase as a function of concentration of the domain added. This cooperative change in dwell time was hypothesized to be due to structural changes in F-actin that are induced by α-catenin’s actin-binding domain binding to F-actin^49^. In addition, dynamic changes in actin filament conformation in response to biochemical perturbations have also been measured using single molecule FRET measurements on dual-labelled actin monomers^24^. Therefore, to obtain a detailed understanding of the actin-binding kinetics of our different mutants and potential effects of F-actin structural conformation on binding, we used a TIRF-based single molecule binding assay to measure binding kinetics (Materials and Methods, In vitro single molecule binding kinetics assay). Our assay consisted of single actin filaments tethered to the surface of a functionalized glass coverslip enclosed in a flow well geometry (Fig. 2a). The surface of the coverslip was passivated with a PEG monolayer containing 5% biotinylated PEG. The flow well was first incubated with excess streptavidin followed by biotin-phalloidin, which resulted in a surface that would tether actin filaments. We then monitored the binding kinetics of single molecules of labelled actin-binding domain to F-actin (at an actin-binding domain concentration of ~0.05–0.1 nM, Fig. S3).

We compared the distribution of binding dwell times for the different mutants identified in our live cell experiments. The average dwell times were similar between utrnWT (τ_av utrnWT_ ~ 1.1 s), utrnLAM (τ_av utrnLAM_ ~ 1.3 s) and utrnΔN (τ_av utrnΔN_ ~ 0.8 s) (Fig. 2b). In contrast, utrnSF had a longer average dwell time (τ_av utrnSF_ ~ 4.6 s), indicating that this mutant turned over more slowly. We then measured the binding on-rate using our single molecule assay by measuring the frequency of binding events for a range of concentrations of actin-binding domain in solution (Fig. 2c). We found that utrnWT (k_on_ = 2.2 ± 0.3 µM^−1^ s^−1^), utrnLAM (k_on_ = 2.4 ± 0.3 µM^−1^ s^−1^), and utrnΔN (k_on_ = 2.1 ± 0.4 µM^−1^ s^−1^) were similar and utrnSF (k_on_ = 5.0 ± 2.0 µM^−1^ s^−1^) had a higher on rate (Fig. 2c). Intrigued by the differences in localization in live cells and similar in vitro binding kinetics of utrnWT, utrnLAM and utrnΔN, we focused our attention on these mutants in particular.

For all of the mutants tested, the distribution of binding dwell-times was well characterised by a double exponential fit (*R*^2^ = 0.99 double exponential, *R*^2^ = 0.94 single exponential, Pearson’s correlation coefficient), suggesting a two-timescale binding model best described the behaviour of these constructs (Fig. 2d–f and Fig. S4, ‘Methods’, ‘In vitro single molecule binding kinetics assay’). By comparison, the common actin binding probe Lifeact was well characterised by a single exponential (*R*^2^ = 0.99, single exponential, Fig. S4d), indicating that the mechanisms of binding for CH1–CH2 domains is more complex than that of the short peptide.

To further evaluate the binding behaviour of the different mutants, we examined the cumulative distribution function (CDF) of binding dwell-times. We measured the characteristic dwell-time (τ_1_ and τ_2_) and relative amplitude (a_1_) of each timescale, making an additional correction for photobleaching in the experiments (Fig. S4f, ‘Methods’, ‘Single molecule analysis’). The two-timescale behaviour of utrnWT (Fig. 2d) was more distinct than the flatter behaviour of utrnLAM and utrnΔN (Fig. 2e), as characterised by a smaller difference in the two timescales (τ_2_/τ_1 utrnWT_ = 18.3, τ_2_/τ_1 utrnLAM_ = 9.6, τ_2_/τ_1 utrnΔN_ = 9.6). utrnLAM had a more even fraction of events belonging to each timescale (a_1 utrnWT_ = 0.8, a_1 utrnLAM_ = 0.7, a_1 utrnΔN_ = 0.8) (Fig. 2g–i).

We have previously shown that the mutations Q33A T36A lie at the interface between CH1 and CH2 and result in an increased binding affinity to F-actin by relieving the steric interaction between CH2 and F-actin^37^. Removing the Q33A T36A mutations from utrnSF and utrnLAM reduced the dwell-times of both mutants, consistent with a reduced binding affinity. However, these mutants retained their characteristic two-timescale response, with K121A being comparatively flatter than G125A L132A (Fig. 2f). Therefore, we speculated that the observed two-timescale binding behaviour could arise from direct interactions with F-actin. We sought to test this idea by measuring the CDF of binding dwell times of these different mutants on actin filaments in different conditions.

### Small molecules that change actin filament conformation alter utrophin ABD mutant dwell times

**Fig. 3:**
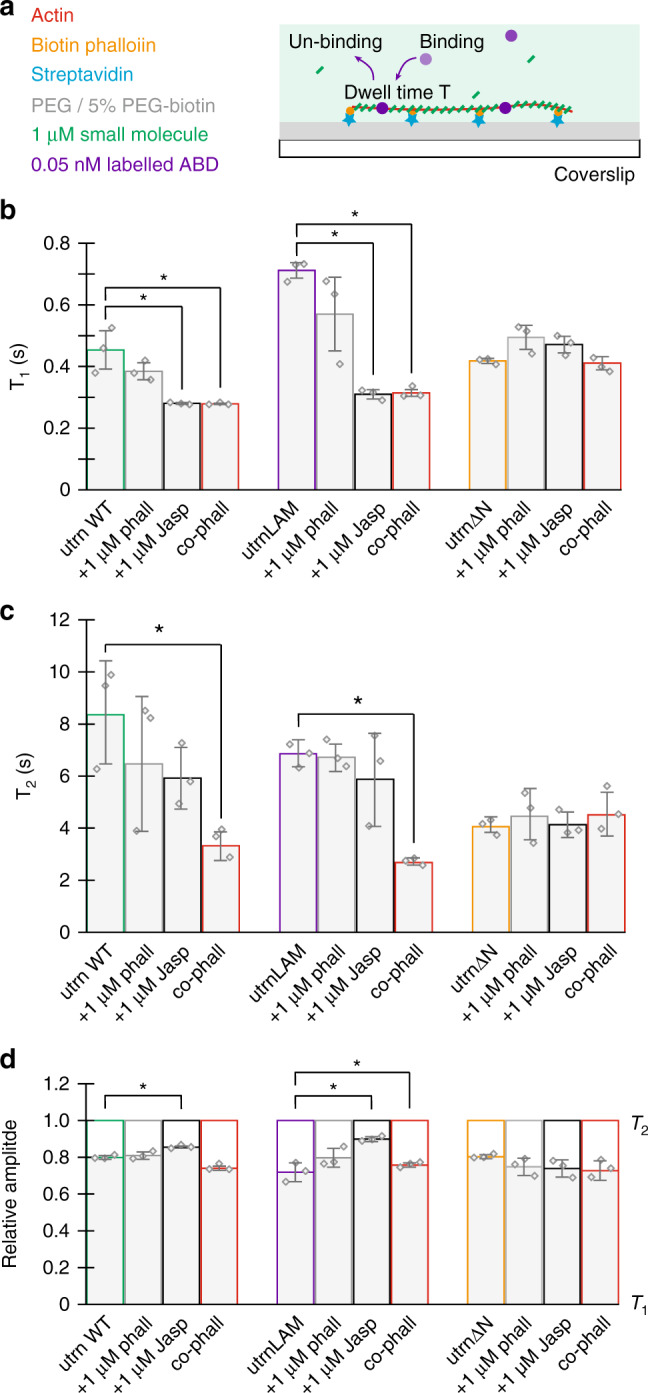
Filament stabilization by small molecules alter utrophin ABD mutant dwell times. **a** Measurement of binding dwell times in the presence of phalloidin and jasplakinolide that stabilize actin filaments. Either 1 µM phalloidin or 1 µM jasplakinolide were added to the assay chamber after actin filaments had been tethered to the glass coverslip. These conditions were also compared with actin filaments co-polymerized with 5 µM biotin-phalloidin which were subsequently added to the imaging chamber. **b** Comparisons of the first timescale, **c** second timescale, and **d** relative amplitudes from a double exponential fit to the cumulative distribution functions for the different constructs and conditions. Error bars are the standard deviation around the mean of 3 technical replicates. Conditions were compared using a two-tailed students *t*-test, with *p* < 0.05 denoted by a star (*).

We next investigated whether stabilization of actin filaments with the small molecules phalloidin and jasplakinolide altered the binding dwell-times of the different utrophin mutants (Fig. 3a). Phalloidin and jasplakinolide have a distinct binding site on F-actin that differs from that of utrophin ABD (Fig. S5). A recent structural study has shown that binding of these small molecules changes the conformation of F-actin at the site where utrophin binds^50^. Phalloidin stabilizes the D-loop of subdomain 2 on F-actin in a closed conformation, while jasplakinolide stabilizes the D-loop in an open filament conformation. We introduced different small molecules into our assay chamber after filaments had been tethered to the surface of the chamber and then used our single-molecule TIRF assay to measure the CDF of each mutant. We found that the introduction of 1 µM phalloidin did not have a significant effect on the binding dwell-time of either utrnWT (τ_1 utrnWT_ = 0.45 ± 0.04 s, τ_1 utrnWT+phall_ = 0.38 ± 0.02 s, *p* = 0.14), utrnLAM (τ_1 utrnLAM_ = 0.71 ± 0.02 s, τ_1 utrnLAM+phall_ = 0.57 ± 0.08 s, *p* = 0.50), or utrnΔN (τ_1 utrnΔN_ = 0.42 ± 0.01 s, τ_1 utrnΔN+phall_ = 0.49 ± 0.02 s, *p* = 0.83) (Fig. 3b–d, Fig. S6a–c). In contrast, introduction of 1 µM of the actin stabilizing agent jasplakinolide had a significant effect on both utrnWT and utrnLAM, reducing dwell-time of single molecules in both cases (τ_1 utrnWT+jasp_ = 0.28 ± 0.01 s, *p* = 0.05, τ_1 utrnLAM+jasp_ = 0.31 ± 0.01 s, *p* < 0.05, Fig. 3b–d). Interestingly, the effect of jasplakinolide was stronger on utrnLAM (~53% reduction in dwell time) than it was on utrnWT (~36% reduction in dwell time), suggesting that each mutant had a different degree of specificity for jasplakinolide-stabilized F-actin. Jasplakinolide treatment appeared to have a small effect on the binding dwell time of utrnΔN (τ_1 utrnΔN+jasp_ = 0.47 ± 0.02 s, *p* = 0.09), which was not statistically significant in our measurements. This result suggests that this mutant was less sensitive to the actin filament conformational change induced by jasplakinolide.

Intrigued by this observation, we used the recent finding that actin filaments polymerized in the presence of phalloidin have a different conformation than actin filaments stabilized by phalloidin after polymerization^50^, to test whether two different F-actin conformations induced by the same drug would be enough to alter utrophin mutant affinity. More specifically, it was found that actin polymerized in the presence of phalloidin results in an open D-loop configuration, while F-actin stabilized with phalloidin post polymerization results in a closed D-loop. Consistent with the structural study, the data we obtained with the filaments polymerized in the presence of phalloidin was similar to that of jasplakinolide treatment for utrnWT (τ_1 utrnWT_ = 0.45 ± 0.04 s, τ_1 utrnWT+co-phall_ = 0.28 ± 0.01 s, *p* = 0.01), utrnLAM (τ_1 utrnLAM_ = 0.71 ± 0.02 s, τ_1 utrnLAM+co-phall_ = 0.31 ± 0.01 s, *p* = 0.01), and utrnΔN (τ_1 utrnΔN_ = 0.42 ± 0.01 s, τ_1 utrnΔN+co-phall_ = 0.41 ± 0.01 s, *p* = 0.07) (Fig. 3b–d and Fig. S6d–f). Taken together, these results show that changes in actin filament conformation induced by small molecules can alter the binding properties of actin regulatory proteins, with utrnLAM and utrnWT preferring the closed D-loop conformation, and utrnΔN being less sensitive to the conformation of the D-loop.

### Filament binding by cofilin and drebrin alters dwell time of utrophin ABD mutants

In addition to the small molecules phalloidin and jasplankinolide, several actin-binding proteins have been shown to impact filament conformation. Cofilin is an actin severing protein that breaks actin filaments by forming discontinuities in filament mechanical properties^36,51^. Non-continuous mechanical properties are caused by changes in filament twist induced by cofilin binding, which change the helical half pitch of F-actin from a mean of ~36 nm to ~27nm^52,53^. Given our observations that utrophin ABD mutants were sensitive to actin filament conformation induced by jasplakinolide, we investigated how the different utrophin ABD mutants interacted with cofilin.

**Fig. 4:**
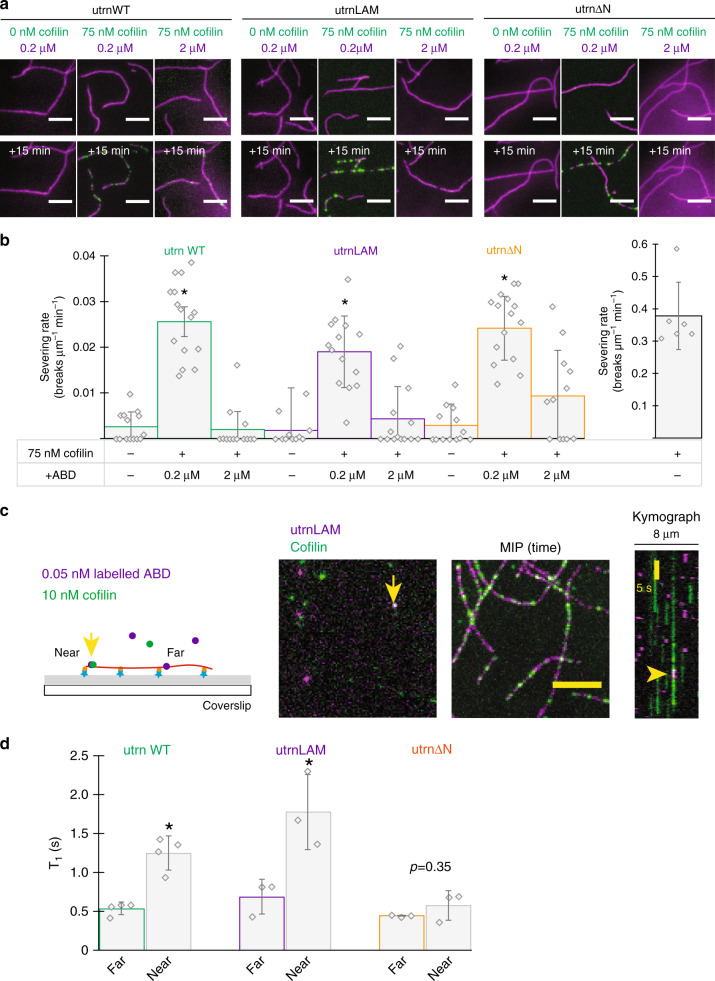
Filament binding by cofilin alters utrophin ABD mutant dwell times. **a** Actin filament severing by 75 nM cofilin (green) in the presence of 0.2 μM and 2 µM of the different utrophin mutants (magenta). Scale bars are 5 µm. **b** Quantification of actin filament severing rate. The error bars represent the standard deviation of more than 12 imaging regions collected from two imaging chambers. **c** Single molecule colocalization and kinetic measurements at low concentrations of cofilin (10 nM) analysed both near and far from a cofilin binding event. Scale bar 5 µm. The kymograph is from an 8 µm section of filament. **d** The first timescale from the fit to the CDF for molecules near (<30 nm) and far (>30 nm) from cofilin for utrn WT, utrnLAM and utrnΔN. Error bars are the standard deviation around the mean of 3 technical replicates. Conditions were compared using a two-tailed students *t*-test, with *p* < 0.05 denoted by a star (*).

First, we measured the severing activity of cofilin in the presence of different utrophin mutants (Fig. 4a). When we introduced either 2 µM or 200 nM of each of the different mutants with 75 nM cofilin to the assay chamber, we found that cofilin binding appeared to be slowed (Fig. 4a images), which was reflected by a reduced severing rate (Fig. 4b). This likely arises due to direct competition for a similar binding site on F-actin. Similar observations have been made for the actin-binding domain of α-catenin and drebrin, which have been found to slow cofilin severing through competitive binding^49,54^. Given that the actin-binding domains (~2 µM^−1^ s^−1^) have a faster on-rate than cofilin (0.06 µM^−1^ s^−1 ^^48^), and that both proteins are introduced into the assay at the same time, we expect that competitive binding plays a role in the slower rate of severing of cofilin in our assay. Next, we sought to test whether conformational changes induced by cofilin binding impacted the dwell time of the different mutants. We used a dual-colour binding assay with a low concentration of labelled cofilin (10 nM), and single molecule levels of utrophin mutant ABD (0.05–0.1 nM). We then sorted the utrophin mutant ABD single molecule binding events based on their distance from cofilin events^48^, which we were able to localize with a precision of ±30 nm (Fig. 4c). Since structural changes in actin induced by cofilin are reported to propagate locally, at distances ranging from 1 to 2 subunits^55,56^, we considered single molecule binding events within 30 nm from a cofilin binding event to be ‘near’ and those beyond 30 nm to be ‘far’. The 30 nm threshold was the highest resolution we could achieve in our measurements. We measured the CDF for near and far cofilin molecules (Fig. S7) and compared the τ_1_ values (‘Methods’, ‘Cofilin near-far measurements’). The dwell time of utrnWT single molecules ‘near’ cofilin was longer than those ‘far’ from a cofilin binding event (τ_1 utrnWTnear_ = 1.24 ± 0.10 s, τ_1 utrnWTfar_ = 0.53 ± 0.04 s, *p* = 0.004). The presence of cofilin had an even stronger effect on utrnLAM (τ_1 utrnLAMnear_ = 1.77 ± 0.30, τ_1 utrnLAMfar_ = 0.68 ± 0.13, *p* = 0.04). The τ_1_ dwell times for utrnΔN ‘near’ cofilin were more similar to those ‘far’ from cofilin (τ_1 utrnΔNnear_ = 0.57 ± 0.11 s, τ_1 utrnΔNfar_ = 0.44 ± 0.01 s, *p* = 0.36) (Fig. 4d). In addition, the τ_1_ values that were ‘far’ from cofilin in these experiments were comparable to those measured in the absence of cofilin (Fig. 2a). These results show that conformational changes in F-actin near a cofilin binding event may feed-back on the binding dwell times of nearby regulatory proteins (CH1–CH2 domain containing proteins).

**Fig. 5:**
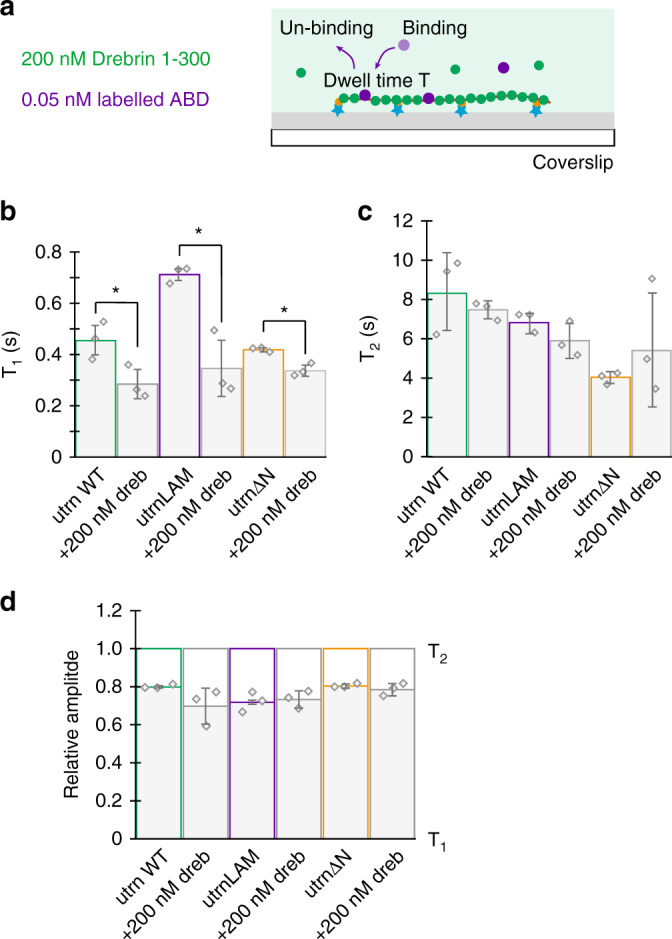
Filament binding by drebrin alters utrophin ABD mutant dwell times. **a** Single molecule binding kinetics in the presence of 200 nM of the actin-binding domain of the protein drebrin (drebrin 1–300). Comparisons of the first timescale **b** second timescale **c** and relative amplitudes **d** from a double exponential fit to the cumulative distribution functions for the different constructs. Error bars are the standard deviation around the mean of 3 technical replicates. Conditions were compared using a two-tailed students *t*-test, with *p* < 0.05 denoted by a star (*).

While cofilin shortens the helical half pitch on actin, the actin binding protein drebrin extends the helical half pitch of an actin filament to a mean of ~40 nm^32,52,57^. We tested the effect of 200 nM of the actin-binding domain of drebrin (AA 1–300) on the utrophin mutants (Fig. 5a and Fig. S6g–i). This concentration decorated actin filaments and reduced the dwell time of both utrnWT and utrnLAM (τ_1 utrnWT+dreb_ = 0.28 ± 0.02 s, *p* = 0.04, τ_1 utrnLAM+dreb_ = 0.35 ± 0.07 s, *p* = 0.03) (Fig. 5b). In addition, drebrin binding also had a smaller but significant effect on the binding lifetime of utrnΔN (τ_1 utrnΔN+dreb_ = 0.34 ± 0.01 s, *p* = 0.02, Fig. 5b). Taken together these results show that structural changes induced by actin-binding proteins, including under-twisting and over-twisting of F-actin, can have an allosteric effect on the kinetic properties of actin-binding domains.

### Myosin binding changes dwell time of utrophin ABD mutants

**Fig. 6:**
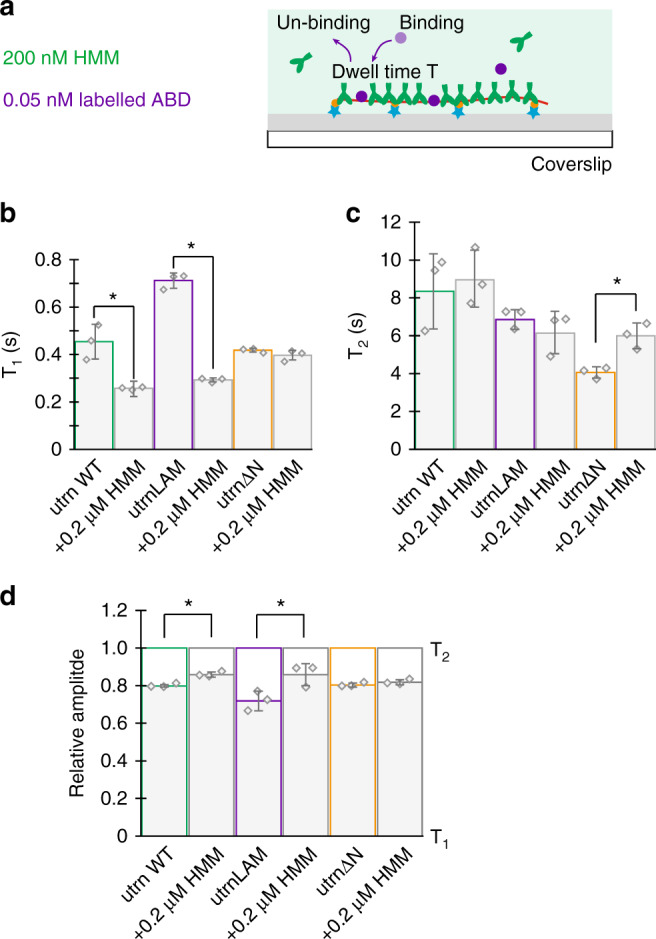
Filament binding by heavy meromyosin (HMM) alters utrophin ABD mutant dwell times. **a** Single molecule binding kinetics in the presence of 200 nM of the myosin fragment HMM. Comparisons of the first timescale (**b**), second timescale (**c**), and relative amplitudes (**d**) from a double exponential fit to the cumulative distribution functions for the different constructs. Error bars are the standard deviation around the mean of 3 technical replicates. Conditions were compared using a two-tailed students *t*-test, with *p* < 0.05 denoted by a star (*).

While cofilin locally remodels actin filaments near the leading edge of migrating cells, myosin generates contractile forces needed for cell migration at the rear of migrating cells^58,59^. Motivated by our observation of differential front-back localization of the utrophin ABD mutants (Fig. 1), we tested if myosin binding influenced the binding of purified forms of the utrophin mutants in vitro (Fig. 6a and Fig. S6j–l). We measured the single molecule dwell times of utrophin ABD mutants in the presence of the myosin fragment heavy meromyosin (HMM). We found that utrnWT and utrnLAM displayed a reduced dwell time in the presence of HMM (τ_1 utrnWT+HMM_ = 0.26 ± 0.02 s, *p* = 0.04, τ_1 utrnLAM+HMM_ = 0.29 ± 0.01 s, *p* = 0.001, Fig. 6b–d). However, HMM binding did not have a significant effect on the binding lifetime of utrnΔN (τ_1 utrnΔN+HMM_ = 0.40 ± 0.01 s, *p* = 0.26).

### Native CH1–CH2 domains display biased localization to different actin structures

**Fig. 7:**
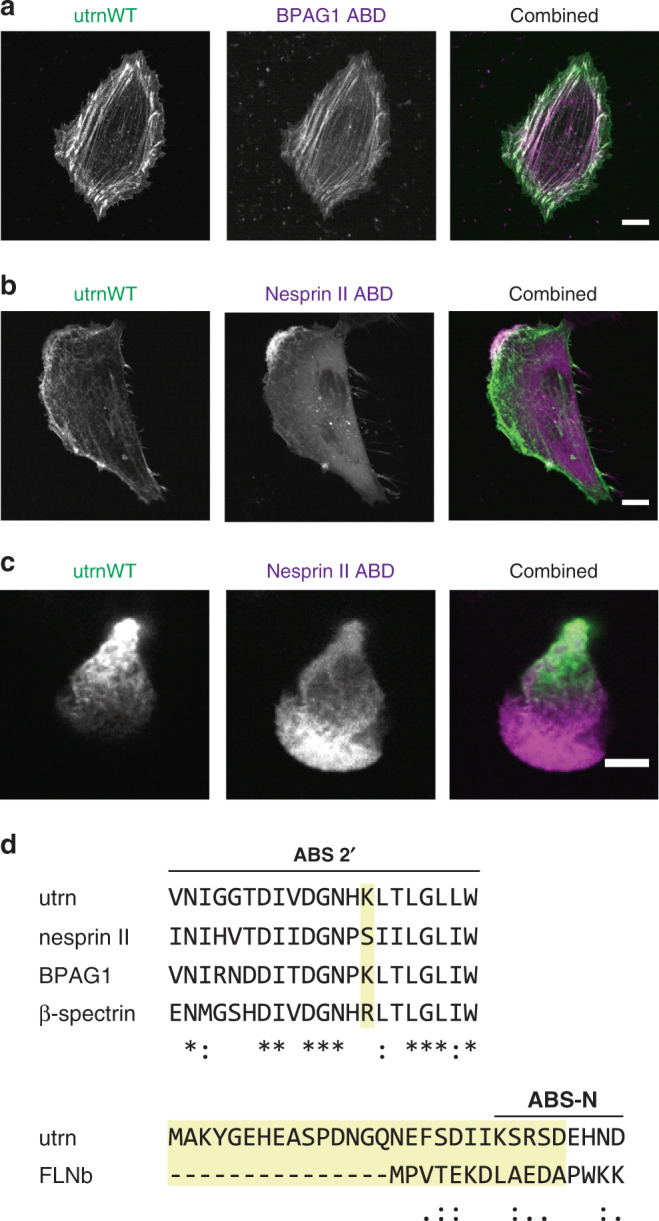
Native CH1–CH2 domains display different sub-cellular localizations. **a** Localization of BPAG1 ABD (magenta) relative to utrnWT (green) in HeLa cells. Scale bar is 5 µm. **b** Localization of Nesprin II ABD (magenta) relative to utrnWT (green). **c** Localization of Nesprin II ABD (magenta) relative to utrnWT (green) in PLB cells. Images shown are representative examples taken from a set of at least 10 different images. Scale bar is 5 µm. **d** Sequence alignment of native CH1–CH2 domains. Residue K121 for utrnWT highlighted in yellow (top) and the N-terminal region prior to CH1–CH2 and its truncation in yellow (bottom). Identical residues are annotated with ‘*’, strongly conserved with ‘:’, and weakly conserved with ‘.’.

Having identified that utrophin ABD mutants localize to different subcellular actin structures and that their binding affinity is altered by changes in actin filament conformation, we wondered if native CH1–CH2 domains displayed similar characteristics. We screened the localization of native CH1–CH2 domains relative to utrnWT (Fig. S9). Native CH1–CH2 domains displayed a range of actin-binding affinities, which we assessed from the relative pools of protein on actin and in the cytoplasm in live cells^37^. We also found that several native CH1–CH2 domains displayed enhanced localization to specific actin structures (Fig. 7 and Fig. S9). For example, the actin-binding domain of dystonin/BPAG1, a protein that links the actin cytoskeleton to other cytoskeletal networks^60^, was enriched on stress fibers in HeLa cells (Fig. 7a and Supplementary Movie S5). In contrast, the ABD of nesprin II, a protein that links the actin cytoskeleton to the nucleus^61^, was enriched in the lamellipodium in both HeLa cells (Fig. 7b and Supplementary Movie S6) and PLB neutrophils (Fig. 7c and Supplementary Movie S7). These results show that native CH1–CH2 domains, in addition to having different overall affinities, show preferential binding to specific actin structures in cells.

## Discussion

Using a combination of live cell imaging, in vitro characterisation, and single molecule binding measurements, we showed that utrophin ABD mutants have varying binding kinetics for different conformational states of F-actin and display differences in localization to actin structures in live cells. We found that two constructs, utrnWT and utrnLAM, had different degrees of specificity for structural changes in F-actin, while utrnΔN was comparatively less sensitive to structural changes. These domains responded to changes in actin filament conformation induced by biochemical perturbations and regulatory protein binding.

The identification of specificity of actin-binding domains to different actin conformations and actin networks has two broad implications for understanding cytoskeletal physiology. Firstly, in addition to generating mutant actin-binding domains from utrophin, we tested the localization of native CH1–CH2 domains. Many of these domains displayed differences in binding affinity, characterised by differences in cytoplasmic signal (Fig. S8), but in addition several actin-binding domains, including nesprin II CH1–CH2 and BPAG1 CH1–CH2 displayed differences in localization to actin structures. These observations highlight that small differences in sequence between native domains are important for both the affinity and specificity to different actin structures and has broader implications for the activity of full-length actin regulatory proteins. One example of this is indeed nesprin II, which has been shown to localise to the front of the nucleus as cells migrate through small constrictions^61^. This localization was dependent on the presence of the actin-binding domain, suggesting that conformational sensing could help to spatially organise this actin binding protein for its specific function. The broader notion that some actin-binding proteins modulate actin filament structure (such as cofilin, formins and myosin), while others can be sensitive to it (CH1–CH2 containing proteins), highlights the role of the actin cytoskeleton as a signalling substrate in its own right, with potential functional significance for a range of biological processes.

Secondly, it is interesting to speculate that CH1–CH2 domains could be used to engineer probes for different structural states of F-actin for use both in vitro and in vivo. In fact, although utrnWT has been commonly used as a marker for F-actin^40^, it has also been reported to localize more preferentially to the trailing edge of migrating cells^62^. C-terminal truncated forms of utrnWT have also been used for labelling of nuclear actin filaments^11^, and direct fusions to GFP via a helical linker have been used in fluorescence polarization studies^63^. We have shown that distinct residues control both affinity^37^ and, in this study, specificity, suggesting that it should be possible to engineer binding domains with a range of desired properties for live cell and tissue studies^37,64^. The ability to tune both the bulk affinity and the specificity of interaction with F-actin using these two mechanisms is highly desirable when designing actin probes so that the contribution of these different effects can be distinguished.

Indeed, it is important to note that actin filament conformation-induced differences in ABD localization likely act in concert with differences in localization that can arise from proteins having different bulk actin-binding affinities. Previous work has shown that high affinity actin-binding proteins such as myosin are depleted from dynamic actin networks due to their slow turnover rate, and by convective flows of actin^10,22^. Consistent with this finding, some of the mutants generated in our initial screen had slow turnover rate (as measured by FRAP) and displayed differences in localization (i.e., utrn Q33A T36A^37^ and utrnSF (Fig. S9)), showing depletion from dynamic actin networks (Fig. S1). By engineering mutant actin-binding domains to have bulk affinities similar to that of utrnWT, we were able to detect biases in binding to actin filaments structures independent of large-scale changes in bulk binding affinity. Our results suggest that specificity of actin-binding proteins to filament conformations could combine with their overall binding affinity to generate a rich landscape of actin binding properties and localizations. This concept could explain why myosin, which binds F-actin with high affinity, also binds cooperatively to actin filaments and displays context-dependent catch bonding behaviour^65,66^, or why highly dynamic Lifeact does not bind to actin decorated with cofilin^13^ or jasplakinolide-stabilized actin^42^, which changes the conformation of the filament. In our experiments, the turnover rates of our mutant ABDs (utrnWT, utrnLAM and utrnΔN) are very similar, based on measurements in live cells using FRAP (Fig. S9, ‘Methods’, Fluorescence recovery after photobleaching) and single molecule photoactivation (Fig. S10, ‘Methods’, ‘Single molecule binding measurements in live cells’), even though they display differential localization in cells. In particular, single molecule kinetics in live cells were indistinguishable between utrnWT and utrnLAM when F-actin structures were homogenised with 50 µM Y27632 treatment (depolymerises stress fibers, Fig. S10). In fact, utrnΔN turned over slightly more slowly than utrnWT, despite its comparative enrichment to more dynamic actin structures (Fig. S10e and Fig. 1e,f).

How do CH1–CH2 domains sense different conformations of F-actin? Recent evidence has suggested that jasplakinolide preferentially biases one state of F-actin, stabilizing the D-loop from subdomain 2 in a more open configuration^50^, which may partially explain our observations. CH1–CH2 domains have been shown to bind actin by making contacts both on and between actin subunits within the same protofilament^41,42^ (n and n + 2). The N-terminal flanking region contacts the N-terminal actin subunit, ABS2 binds between the two subunits (where the subdomain 2 D-loop from subunit n, contacts subdomain 1 from subunit n + 2) and ABS2’ contacts subunit n + 2. Making several contacts on and between neighbouring F-actin subunits could explain why utrophin’s ABD is sensitive to filament level structural changes induced by these small molecule agents. Indeed, the K121A mutation in utrophin corresponds to a key interaction with the D-loop of subdomain 2, and may explain why mutating this residue changes the degree of specificity to jasplakinolide-stabilized actin. Sequence alignment of native CH1–CH2 domains revealed that this residue is well conserved between domains, though some differences do exist (Fig. 7d). Interestingly, K121 is changed to serine in nesprin II which also enriched to lamellipodial actin in a similar fashion to utrnLAM (Q33A T36A K121A). In our previous work we have shown that ABS-N is important for localization of CH1–CH2 domains. In particular, Filamin B which has a short N-terminal flanking region displayed a similar localization pattern at focal adhesions to utrnΔN^37^ (Fig. 7d). Here, we extend this observation by showing that this region also appears to have a crucial role in actin filament conformational sensing. In all of the conditions tested utrnΔN showed little to no difference in binding dwell time, suggesting it is less sensitive to differences in F-actin conformation.

In addition to actin drugs, we show that actin-binding proteins that modify actin filament conformations such as drebrin, myosin, and cofilin, impact the binding affinity of the different utrophin mutants. While in vitro off-rate measurements provide a precise measurement of binding rates in a controlled environment, further work will be needed to dissect the contribution of different mechanisms that could influence actin filament conformation. For example, the role of different actin isoforms was not assessed here, and the reported binding dwell times include the conformational effects of different actin filament tethering strategies.

Our data suggests that filament structural conformations can be biased by physical forces exerted on them by binding of proteins and drugs. Actin-binding proteins that change the helical pitch of actin, including cofilin, drebrin and formins, exert a torque on the filament^20,36^. This is also the case for myosin II, which steps at a distance shorter than the helical half pitch of an actin filament, causing filaments gliding on a myosin-coated surface to spiral^66,67^. Since actin filaments are inherently helical in nature, torsion and bending are believed to be coupled to twisting^68^ and could arise as filaments are tethered to the glass surface, in a similar fashion to the dynamic conformational changes in actin filaments shown by single molecule FRET^24^. While the mutagenesis study performed here highlights significant functional roles for different residues on CH1–CH2 domains, structural work will be needed to identify the binding mechanisms in more detail and how these different regions combine with overall bulk affinity to give rise to unique actin binding properties.

## Methods

### Generation of constructs

To visualize the relative localization of fluorescent fusions to actin-binding domains, we generated both bi-cistronic expression plasmids for transient transfection and two separate lentiviral plasmids for creating double expression stable cell lines^37^. Mutations to the actin-binding domain of utrophin were introduced by PCR (Supplementary Table 1). Two sets of primers containing the point mutation were used to amplify two separate segments of mCherry-utrn ABD (or in some cases EGFP-utrn ABD or RubyII-utrn ABD) which were then assembled using Gibson assembly into a PCS2 + backbone for transient transfection. To generate constructs for expressing native actin-binding domains, DNA was synthesized by IDT and inserted into the backbone by Gibson assembly. Transient transfections were performed using effectene (Qiagen, #301425), following the manufacturer’s protocol and imaged 24 h after transfection. For generating stable cell lines GFP-utrn ABD and the construct of interest fused to mCherry were amplified by PCR and inserted into the Lentiviral plasmid pHR using Gibson assembly. Lentiviruses were then generated by transfecting the plasmids into HEK293 cells for viral packaging using Transit 293. Lentiviral supernatants were collected 48 h after infection, filtered using a 0.4um filter and used directly to infect the target cell line in a 1:1 ratio with normal culture media. PLB cells were infected by centrifuging cells at 300 rcf for 10 min in lentiviral supernatant containing polybrene. For generating plasmids for protein expression and purification, the actin‐binding domain of human utrophin (CH1–CH2, AA 1–261) was cloned into a modified pETM vector containing an N‐terminal TEV‐cleavable His10‐z‐tag and a C‐terminal Lys‐Cys‐Lys‐(KCK)‐tag for maleimide labelling. Mutations were introduced with PCR and Gibson assembly.

### Protein purification and labelling

Actin was purified from rabbit muscle acetone powder by extraction, polymerisation, ultracentrifugation and then de-polymerisation (Pel Freez Biologicals, #41995-1)^69^. Actin and stored in monomeric form in G-buffer (2 mM Tris-Cl, pH 8.0, 0.2 mM ATP, 0.5 mM TCEP, 0.1 mM CaCl_2_, pH 8.0) at 4 °C. Utrophin’s actin-binding domain (CH1–CH2, AA 1–261) and its associated mutants were expressed recombinantly in E. coli BL21 (DE3) pLysS (Promega, #L1191). Cells were lysed by sonication and HIS tagged protein containing a SUMO solubility tag were purified by IMAC using a 5 mL HiTrap. The solubility tag was cleaved off using TEV protease which was also HIS tagged, and removed by recirculation over the HiTrap column. Finally, proteins were purified by size exclusion chromatography. Proteins were stored in 20 mM Tris-Cl, pH 7.5, 150 mM KCL, 0.5 mM TCEP and 0.1 mM EDTA (Storage buffer) and snap frozen in the presence of 20% glycerol. Utrophin ABD sequences included a KCK linker (GGSGKCKSA) on the C terminus for labelling. Proteins were labelled using either Alexa-488 C_5_ maleimide, Alexa-555 C_2_ maleimide or Alexa-647 C_2_ maleimide (ThermoFisher, #A10254, #A20346, #A20347). Firstly, proteins were reduced with 5 mM TCEP for 30 mins on ice. The buffer was then exchanged to storage buffer lacking TCEP and incubated with 5-fold excess dye for a minimum of 2 h for labelling. Free dye was then removed by size exclusion chromatography using a Superdex 200 column. The minimal actin binding portion of drebrin AA1-300, was purified and labelled using the same strategy. Acanthamoeba α-actinin and Atto488-ybbr-hCofilin^49^ were a kind gift from Peter Bieling (Max Plank Institute of Molecular Physiology, Dortmund).

### Surface functionalization and flow well assembly

Single filament assays were performed in a flow well configuration consisting of a functionalized coverslip and passivated counter-surface assembled using Tesa double sided tape. Glass slides (VWR, #48300-047) were plasma cleaned then passivated using PLL-PEG (g = 3.5), by incubating with ~3 mg/mL PLL_PEG for ~5 mins followed by extensive washing with ultrapure water and air dired. 22 × 22mm coverslips (Zeiss, #474030-9020-000) were passivated using PEG-silane chemistry^70^. Firstly, glass coverslips were cleaned with 3 N NaOH, rinsed in miliQ water, piranha cleaned, rinsed and dried, and then incubated with GOPTS ((3-Glycidyloxypropyl)trimethoxysilane (Sigma #440167)) for 1 h at 75 °C. After silanizaion, the coverslips were rinsed in anhydrous acetone and dried. PEG was coupled to the silanized surface by preparing a PEG saturated acetone solution at 95% hydroxy-amino-PEG (Rapp Polymere, #10 3000-20) and 5% biotinyl-amino-PEG (Rapp Polymere, #13 3000-25-20) which was incubated for a minimum of 4 h at 50 °C. PEG passivated coverslips were then rinsed in miliQ, stored at room temperature and used within 1 month.

### Total internal reflection fluorescence microscopy (TIRF)

TIRF microscopy was used for measuring single molecule binding kinetics in cells and in vitro. The imaging system consisted of a Nikon TIRF inverted scope (Nikon Eclipse Ti, 488/560/642 nm OPSL lasers) with perfect focus, a ×100 N.A. 1.4 APO TIRF oil objective, and an EMCCD camera (Andor iXon Ultra). Hardware was controlled using MicroManager 1.4.

### In vitro single molecule binding kinetics assay

To evaluate the binding properties of different utrophin ABD mutants, single molecule binding kinetics were measured. Actin filaments were polymerized at a final concentration of 5 µM at room temperature in assay buffer (25 mM Immidizole, 25 mM KCl, 4 mM MgCl_2_, 1 mM EGTA, 1 mM DTT, pH 7.4). To immobilize actin filaments to the surface of the flow chamber, flow wells were first incubated with 10 µg/mL streptavidin (Sigma #S0677) for 1 min, washed with assay buffer and then incubated with 1 µM biotin phalloidin, (ThermoFisher #B7474) for 1 min. Actin filaments were then diluted 50× in assay buffer and immediately introduced into the flow well and allowed to attach for 5 min. To prevent shearing of filaments, pipette tips were cut when pipetting actin filaments. Remaining filaments were washed away with assay buffer containing Beta Casein (25 mM Immidizole, 25 mM KCl, 4 mM MgCl_2_, 1 mM EGTA, 1 mM DTT and 10 µg/mL Beta Casein (Sigma C6905)). Binding proteins were diluted to a sufficiently low concentration to enable the visualisation of single molecules in TIRF, 0.05–10 nM in assay buffer. For single molecule kinetic measurements 600 frames were acquired at an interval of 30–130 ms depending on the construct.

### Single molecule analysis

Single molecules were identified and tracked using the TrackNTrace software package^71^. A custom written MatLab routine was then used to post-process the particle tracks and calculate binding dwell-times. As a first step, a maximum intensity projection (MIP) through time of the single molecule movie was used to identify the filament backbone (Fig. S3). An image mask was generated from the MIP by thresholding above the background intensity, and made contiguous by image closure. The MIP mask was then used to filter out single binding events in the maximum intensity projection which did not reside within filament backbone. This filter excluded a small fraction of total events. Binding measurements were then calculated from single molecule tracks that occurred within the filament masked regions.

To calculate the binding on-rate, the length of actin filaments within an image was calculated from the MIP mask by skeletonization. The on-rate was then calculated as the total number of events that occurred during the time of the single molecule movie, for a given number of available binding sites^48^. This measurement was made for a range of concentrations of binding protein and evaluated using linear regression between binding frequency and concentration.

For dwell time measurements, the population of recovered single molecule binding events for different actin binding mutants were analysed in two different ways. Firstly, the average dwell time (τ_av_) for the entire population was measured as a metric for bulk binding dwell time. Secondly, the cumulative distribution function (CDF) of binding dwell times was calculated and fitted with a two-timescale binding model. $\left( {1 - {\mathrm{CDF}}} \right) = a_1e^{ - t/\uptau _1} + (1 - a_1)e^{ - t/\uptau _2}$. Pearson’s correlation coefficient was used to compare single versus two-timescale models (Fig. S4). The two-timescale binding model yields values for the relative amplitudes of each timescale (*a*)—the abundance of binding events belonging to each timescale, and the characteristic dwell time (τ_1_ and τ_2_) which is the inverse of the off-rate. We made additional measurements to account for the effect of photobleaching on dwell times. We measured the rate of photobleaching of the dye used in these experiments (Alexa-555), by imaging Alexa-555 tagged streptavidin with the same experimental conditions (Fig. S4f), which has a high affinity for the biotin peg substrates. We report CDFs as the raw data but make a correction for the characteristic dwell times (τ) reported in bar charts. The bleaching correction was implemented by subtracting the bleaching rate from the measured off-rate: $\frac{1}{\tau\; corrected}=\frac{1}{\tau\; measured}+\frac{1}{\tau\; bleaching}.$

### Cofilin near-far measurements

To evaluate the effect of cofilin binding on the dwell times of utrophin mutants we compared dwell times near and far from a cofilin binding event. Dual colour imaging was used to image 10 nM Atto-488-Cofilin and Alexa-555-utrn, which we imaged with a rate of 450 ms per frame. This concentration in our assay limited the formation of cofilin clusters and the majority of cofilin events were single molecule level. The same single molecule analysis was used for single channel imaging with the additional separation of events being either near or far from a cofilin binding event. Because binding events ‘near’ cofilin were a small fraction of the total events measured we chose to fit τ_1_ only, fixing τ_2_ and the relative amplitude to be the same as the value for ‘far’ events. We then made statistical comparisons between different chambers as technical replicates.

### Actin filament severing

To evaluate the effect of utrophin actin-binding domain (ABD) mutants on the rate of cofilin severing, we monitored the breaks in actin filaments occurring over time. Different concentrations of utrophin ABDs were compared, either 200 nM or 2 µM in the presence of 75 nM cofilin. Both of these proteins were added to the assay chamber at the same time, after filaments had been attached to the glass coverslip.

### Cell culture

HeLa (Homo sapiens, epithelial, ATCC CCL-2) and HEK293 (Homo sapiens, epithelial, ATCC CRL-1573) cells were cultured at 37 °C in an atmosphere of 5% CO_2_ in air in DMEM (Gibco, #10566024) supplemented with 10% FBS (Gibco, #16140071) and 1% penicillin–streptomycin (Gibco, #15140122). Adherent cells were passaged at a 1:5 dilution using 0.25% trypsin EDTA (Gibco #25200056). PLB cells were a kind gift from Dr. Sean Collins (UC Davis). PLB cells were cultured in RPMI (Gibco, #11875093) containing 10% FBS and 1% Penicillin–Streptomycin and differentiated into neutrophil like cells by adding 1.5% DMSO for 5–6 days.

### Cellular confocal imaging

Cells expressing fluorescent fusion proteins were imaged using the following excitation and emission: GFP was excited at 488 nm and emission was collected at 525 nm, mCherry was excited at 543 nm, and emission was collected at 617 nm. Live imaging experiments were performed in normal cell culture media using an OKO labs microscope stage enclosure at 37 °C in an atmosphere of 5% CO_2_. Cells were imaged on glass bottomed 8 well chambers that had been coated with 10ug/ml fibronectin in PBS for 30 min. Cells were imaged with a 60x oil immersion objective N.A. 1.4. Hardware was controlled using MicroManager 1.4.

### Fluorescence recovery after photobleaching (FRAP)

To assess the turnover kinetics and mobility of utrnABD mutants fluorescence recovery after photobleaching (FRAP) experiments were performed. FRAP measurements were performed specifically on stress fibers in HeLa cells. The turnover of different mutants was measured by bleaching a 6pixel diameter spot (~1 µm) using a scanning laser confocal microscope (Zeiss LSM 880 with Airyscan). Fusion constructs to mCherry were used in FRAP experiments. To analyse FRAP data, time lapse stacks were imported into Fiji and bleached regions analysed as ROI. FRAP data were bleaching corrected by measuring the bleaching rate in a ROI far from the bleaching spot which was then subtracted from the recovery curve^72^. The initial rate of recovery found from the initial slope of the recovery curve using MatLab.

### Single molecule binding measurements in live cells

To complement the kinetic measurements in live cells using FRAP on stress fibers, we used photoconversion and single molecule binding measurements. Mutants of interested were generated as fusions to mEOS for single molecule photoactivation with TIRF microscopy. Because cells contain a range of different actin structures that could influence the binding kinetics results, we pre-treated cells with 50 µM of the ROCK inhibitor Y27632 for 30 min, to depolymerize stress fibers. Single molecules were then activated with a 30 ms pulse of 405 nm light in TIRF, and then imaged with 561 nm excitation at an interval of 50 ms. Single molecules were identified and tracked using the TrackNTrace software package^71^. A custom written MatLab routine was then used to post-process the image tracks and calculate binding dwell-times.

### Statistics

Statistical significance was determined by a two-tailed student’s *t*-test and assumed significant when *p* < 0.05. For single molecule dwell time measurements, individual replicates were considered to be individual imaging chambers imaged on different days. Three replicates were measured for each condition. The error bars on bar charts reporting dwell times are the standard deviation of these three replicates. The total number of molecules used to generate the CDF is shown next to the CDF in the figures. τ values in the text are reported as the mean ± standard error. For severing rate measurements, the error bars represent the standard deviation of more than 12 imaging regions collected from two imaging chambers. Scale bars are given in the figure legends.

### Reporting summary

Further information on research design is available in the Nature Research Reporting Summary linked to this article.

## Supplementary information

Supplementary Figures

Peer Review File

Supplementary Movie 1

Supplementary Movie 2

Supplementary Movie 3

Supplementary Movie 4

Supplementary Movie 5

Supplementary Movie 6

Supplementary Movie 7

Reporting Summary

Description of Additional Supplementary Files

## Data Availability

Data supporting the findings of this paper are available from the corresponding author upon reasonable request. A reporting summary for this article is available as a Supplementary Information file. Source data are provided with this paper. The following accession codes were used for generating illustrations of actin filaments and binding proteins in Fig. 1 and Fig. S5 (https://www.rcsb.org/). 1QAG, 6M5G, 6T20, 6T23, 6T1Y, 3J0S.

## Code availability

The illustration of actin filaments and binding proteins were generated using CellScape molecular visualization software developed by Jordi Silvestre-Ryan (https://github.com/jordisr/cellscape). MatLab code to analyse single molecules are available from the corresponding author upon reasonable request.
