# Supplementary material for: Biased localization of actin binding proteins by actin filament conformation
Source: Nat Commun. 2020 Nov 25;11:5973. doi: 10.1038/s41467-020-19768-9 (PMC7688639; doi:10.1038/s41467-020-19768-9)
Supplement: Supplementary file 1 — Supplementary Figures [file 41467_2020_19768_MOESM1_ESM.pdf]

SUPPLEMENTARY INFORMATION

Table S1: List of primers

Primer name	Sequence
pHR Cherry-fwd	GAGAATTCTCACGCGTGCCACCGGATCCAAACCATGGTGAGCAAGGGCGAGGAGGATAAC
pHR utrn-rev	CCTGCAGGTCGACTCTAGAGTCGCGGCCGCCTCGAGTTAGTCTATGGTGACTTGCTGAGG
pHR Cherry-utrnΔN-fwd	CGGCGGCATGGACGAGCTGTACAAGTCCGGAACCGAACACAATGACGTAGCGAAGAAAGCC
pHR Cherry-utrnLAM-fwd	GGAACTGACATTGTGGATGGAAATCACGCGCTGACTTTGGGGTTACTTTGGAGCATC
pHR Cherry-utrnLAM-rev	GATGCTCCAAAGTAACCCCAAAGTCAGCGCGTGATTTCCATCCACAATGTCAGTTCC
pHR Cherry-utrnSF-fwd	GAAATCACAAACTGACTTTGGCGTTACTTTGGAGCATCATTGCGCACTGGCAGGTGAAAG
pHR Cherry-utrnSF-rev	CTTTCACCTGCCAGTGCGCAATGATGCTCCAAAGTAACGCCAAAGTCAGTTTGTGATTTC
pHR EOS-fwd	GGAGCTCTCGAGAATTCTCACGCGTGCCACCATGAGTGCGATTAAGCCAGACATGAAGAT
pHR EOS-utrnΔN-fwd	GGATTGCCTGACAATGCCAGACGATCCGGAACCATGGAACACAATGACGTAGCGAAG
Petm60 utrn-fwd	GCGGGTGAGAATCTTTATTTTCAGGGCGCCATGGCCAAGTATGGAGAACATGAAGCCAGT
Petm60 utrn-rev	GGTGGTGGTGCTCGAGTGCGGCCGCTCATTACGCAGATTTGCATTTACCAGAACCACCG
Petm60- utrnΔN-fwd	GCGGGTGAGAATCTTTATTTTCAGGGCGCCATGGAACACAATGACGTACAGAAGAAAACC

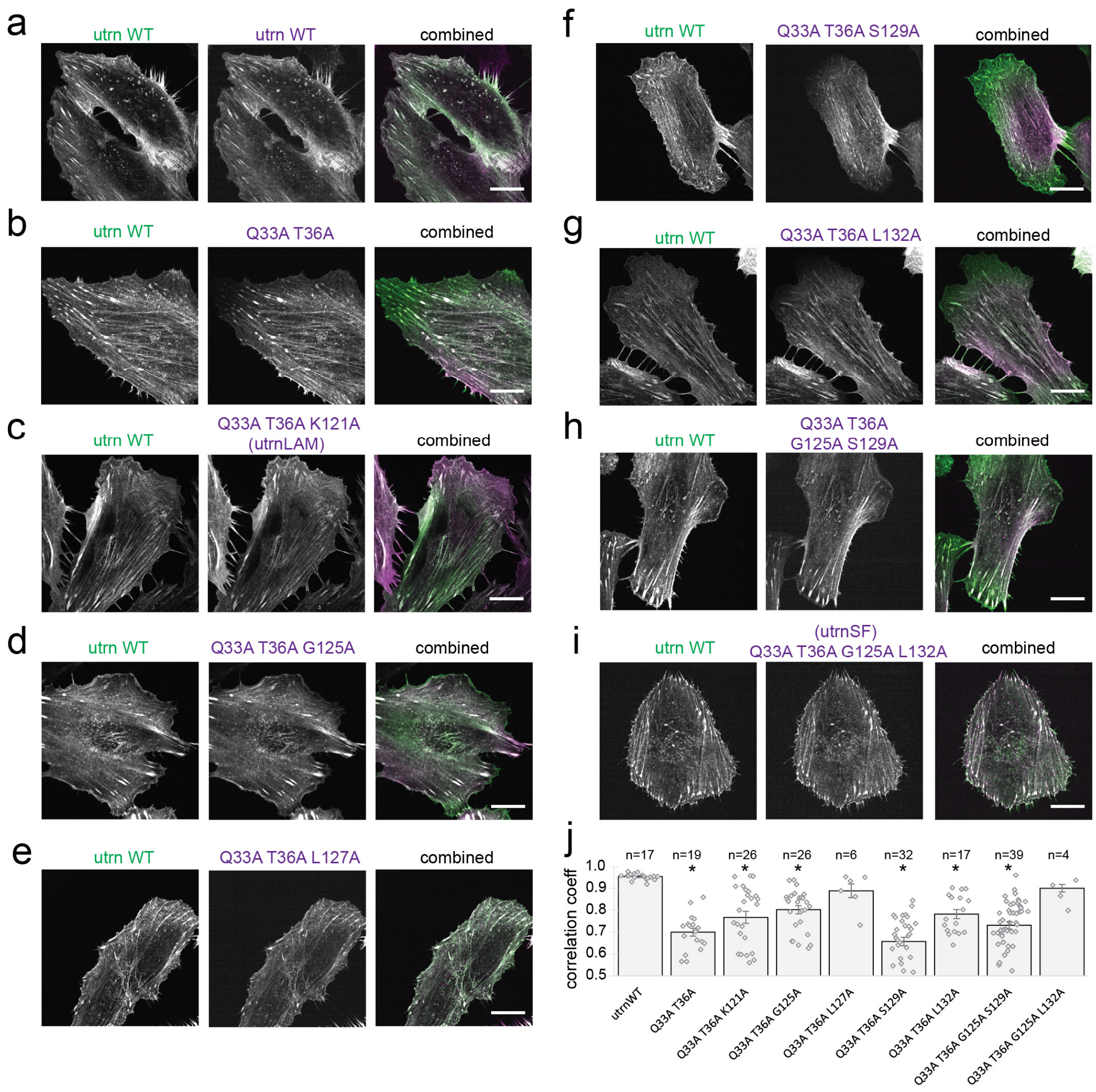

Figure S1: Localization of utrn CH1 mutants

(a-i) Localization of alanine mutations to different residues on utrn CH1 shown in HeLa cells. utrnWT is shown in green and the mutant in magenta. Scale bars are 10μm. (j) Quantification of localization by Pearson's correlation coefficient for the two-color channels. Error bars represent the standard error on the mean.

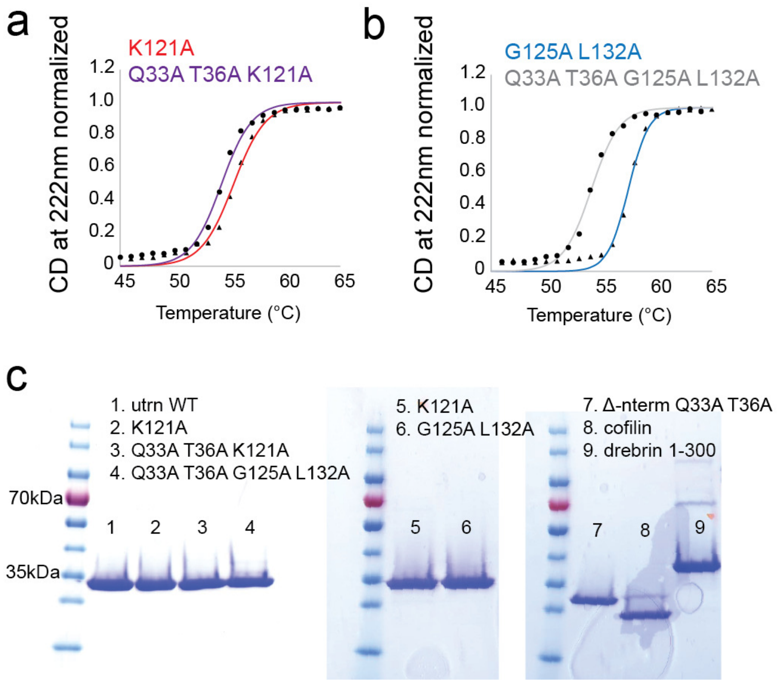

Figure S2: In vitro characterization of utrn CH1-CH2 mutants and purified proteins

(a) Melting temperature curves for K121A (red) and Q33A T36A K121A (purple). (b) Melting temperature curves for G125A L132A (blue) and Q33A T36A G125A L132A (grey). The results shown are representative examples for melting temperature curves. (c) SDS page gels for the purified proteins.

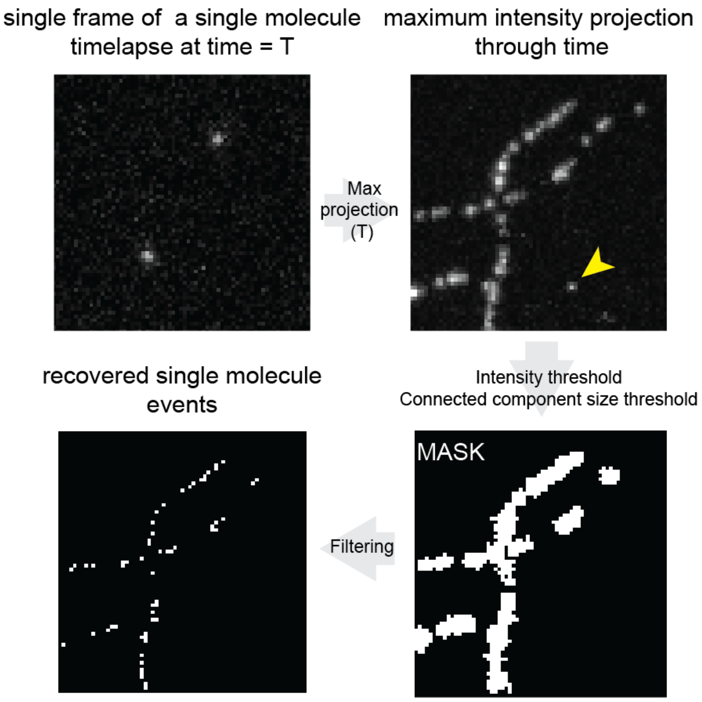

Figure S3: Single molecule image processing

Binding events that occurred outside of the filament backbone were filtered out with the following approach. The maximum intensity projection in time of the single molecule timelapse was generated. Intensity thresholding was then used to make a binary mask for assessing whether binding event occurred within the filament backbone. Events occurring outside of the filament backbone were excluded from the analysis.

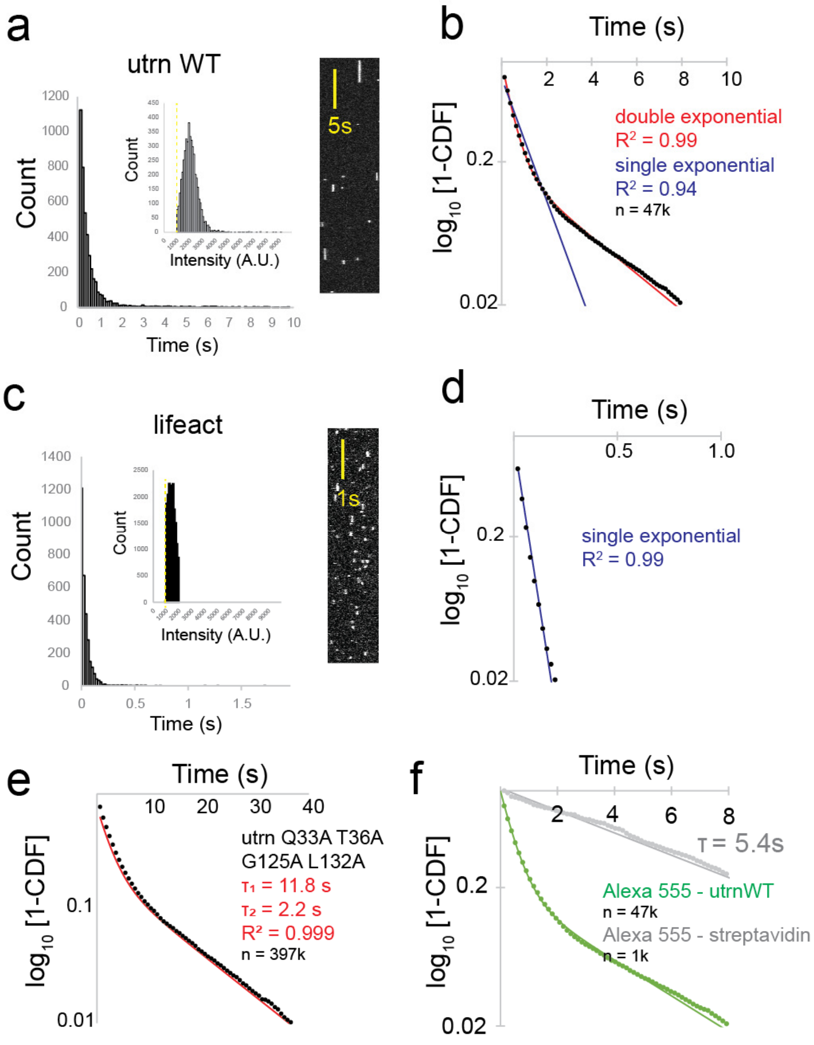

Figure S4: Calibration of single molecule binding kinetics

(a) Dwell time histogram, intensity histogram and kymograph for utrnWT. (b) Cumulative distribution function for utrnWT fit with either a single (blue) or double exponential (red). (c) Dwell time histogram, intensity histogram and kymograph for Lifeact. (d) Cumulative distribution function for Lifeact, fit with a single exponential. (e) Cumulative distribution function for utrn Q33A T36A G125A L132A fit with a double exponential. (f) Characterization of alexa 555 bleaching rate in the experiments. In green the cumulative distribution function for utrnWT-alexa555, and in grey the bleaching rate of alexa555-streptavidin imaged with the same conditions.

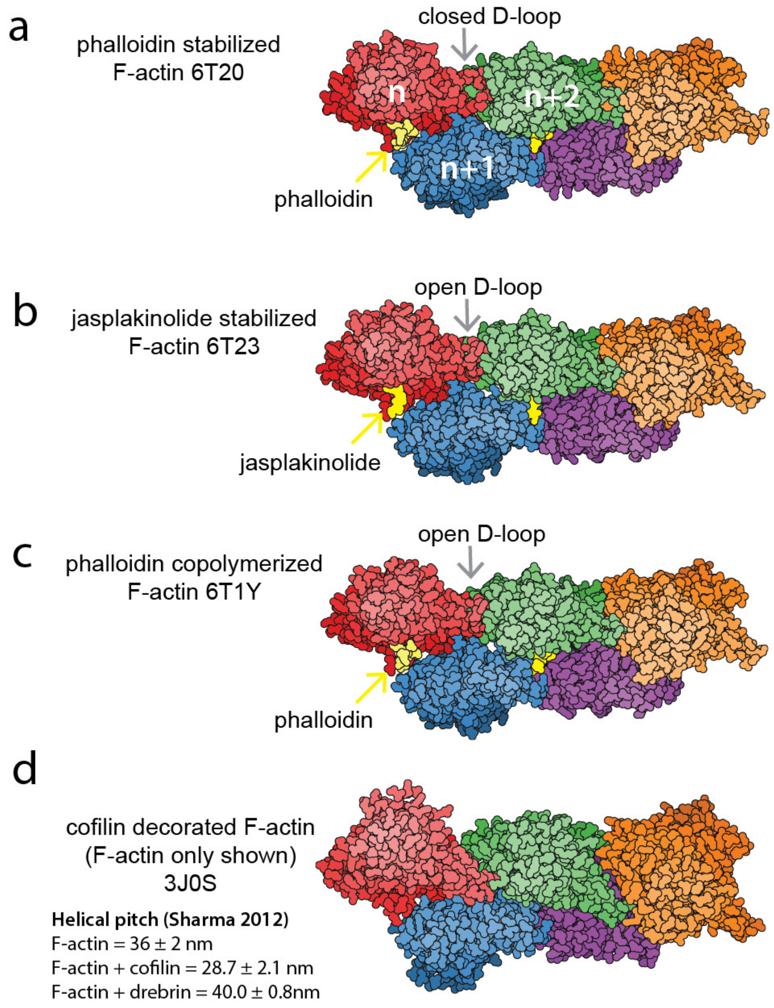

Figure S5: Representations of different F-actin conformations induced by phalloidin, jasplakinolide and cofilin

(a) F-actin stabilized with phalloidin after polymerization (6T20). (b) F-actin stabilized with jasplakinolide after polymerization (6T23). (c) F-actin polymerized in the presence of phalloidin (6T1Y). (d) Cofilin decorated F-actin, with just the actin filament shown here (3J0S).

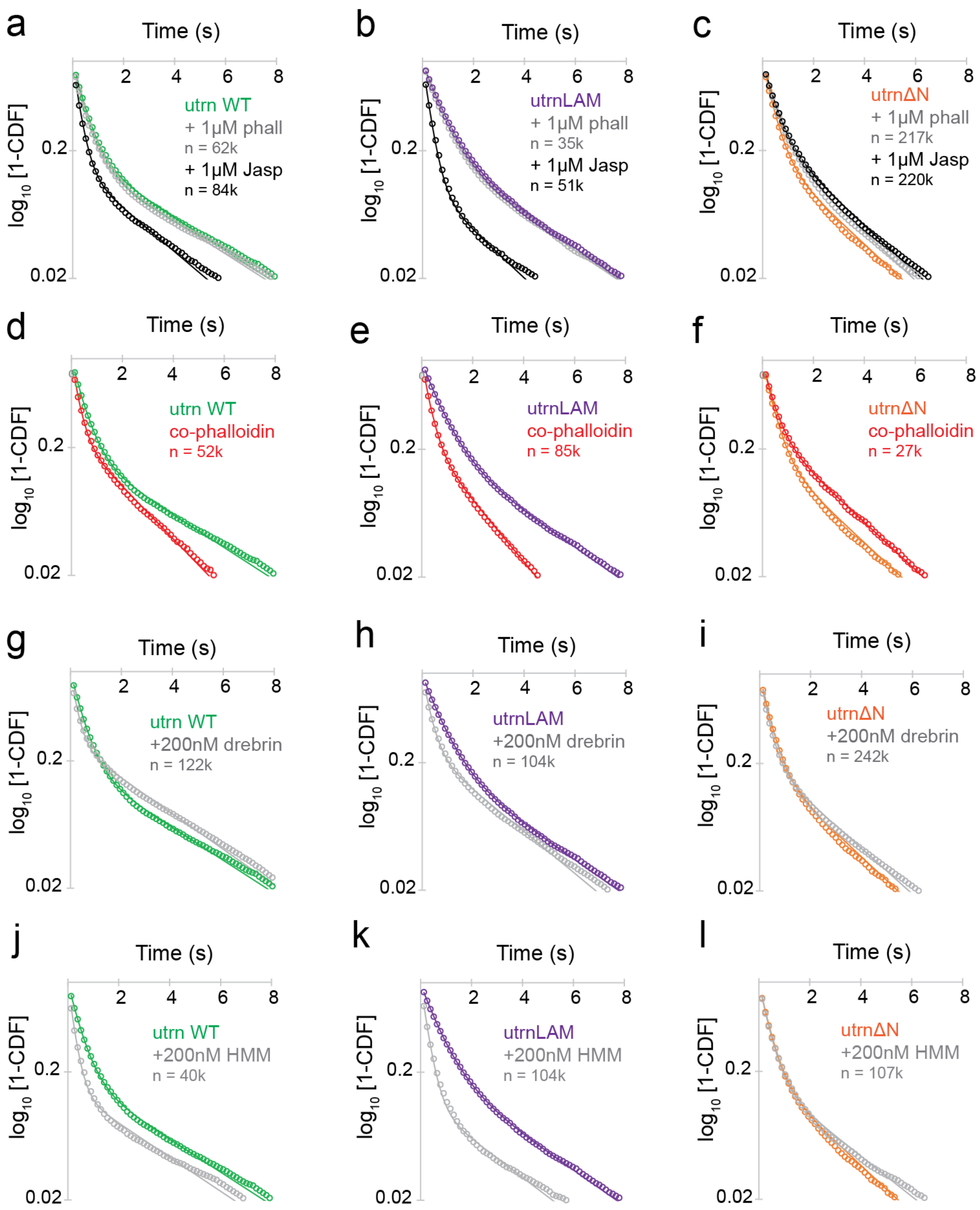

Figure S6: Cumulative distribution functions (CDF) for different constructs and conditions. (a-c) In the presence of either 1μM phalloidin or 1μM jasplakinolide. (d-f) F-actin polymerized in the presence of biotin-phalloidin. (g-i) In the presence of 200nM drebrin. (j-l) In the presence of 200nM HMM. The concentration of the utrophin mutants in the single molecule measurements is 0.05-0.1nM.

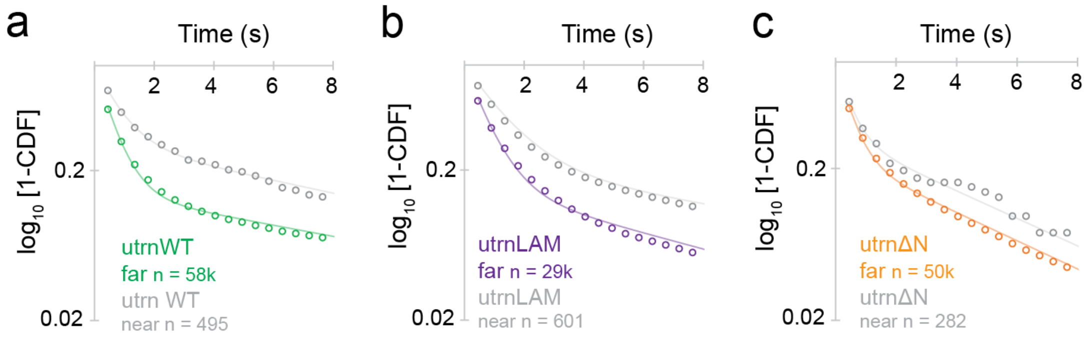

Figure S7: Cumulative distribution functions (CDF) for different constructs near and far from cofilin binding events. (a) utrnWT (green) (b) utrnLAM (magenta) (c) utrnΔN (orange) far from a cofilin binding event and near a cofilin binding event (grey). The concentration of cofilin is 10nM and the concentration of utrn mutants is 0.05nM.

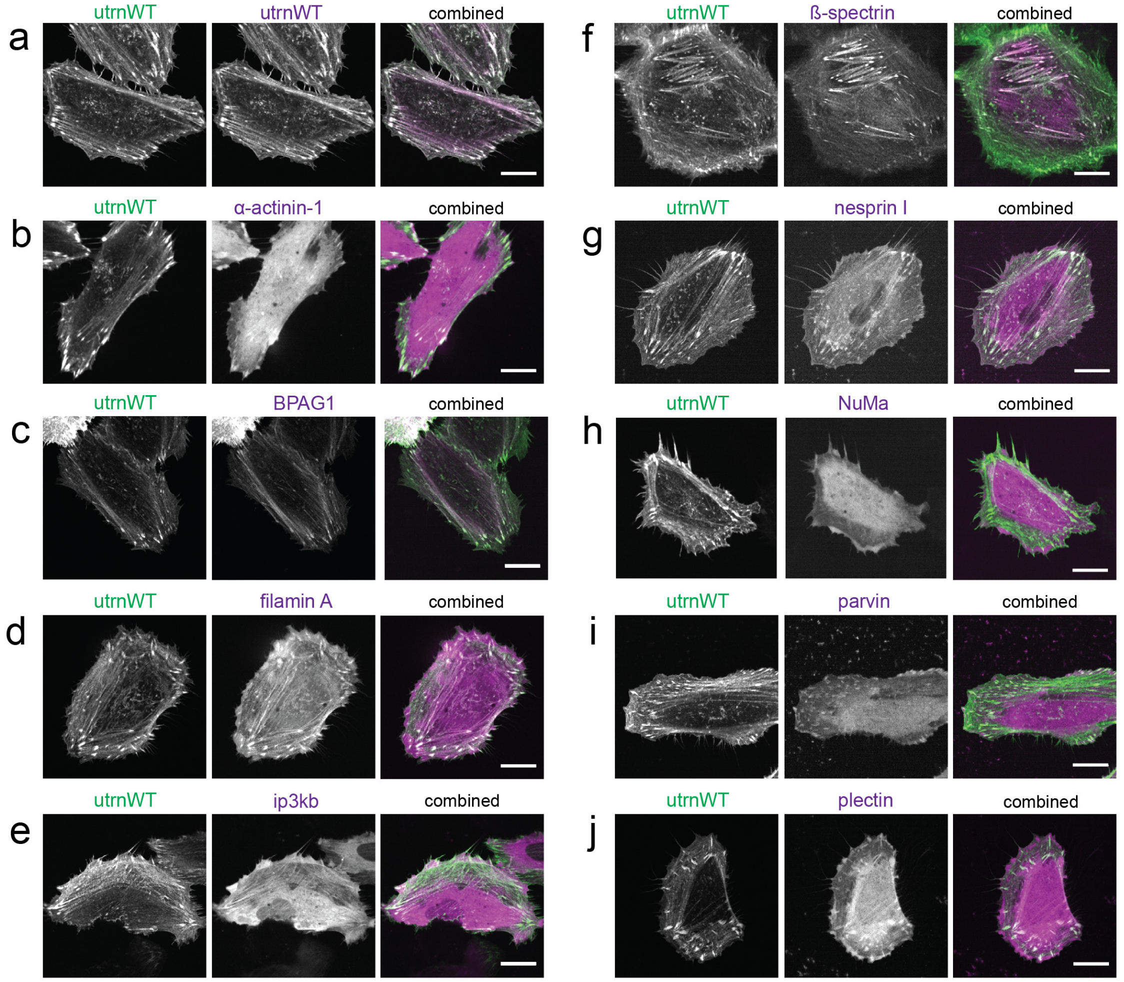

Figure S8: Localization of native CH1-CH2 domains

Localization of native CH1-CH2 domains shown in magenta relative to utrnWT shown in green. (a) utrnWT, (b) α-actinin 1, (c) BPAG1, (d) Filamin A, (e) ip3kb, (f) β-spectrin, (g) nesprin I, (h) NuMa, (i) parvin, (j) plectin. Scale bars are 10μm.

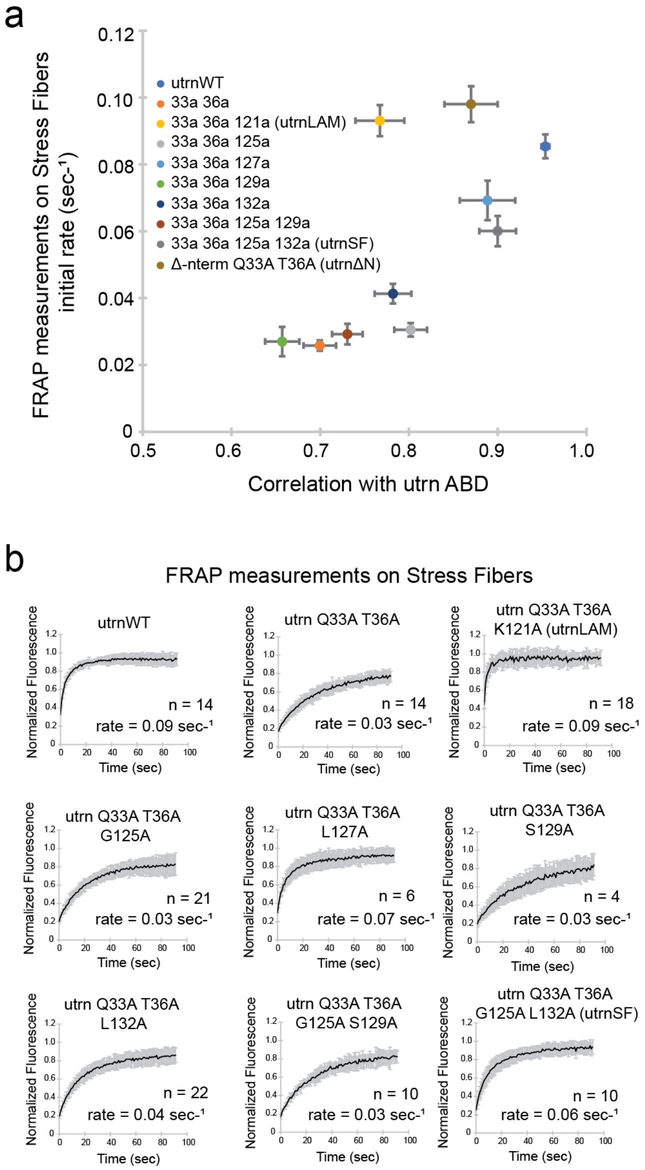

Figure S9: Comparison of localization and recovery rate on stress fibers for utrn CH1-CH2 mutants

(a) Comparison of Pearson's correlation coefficient and the initial recovery rate of different utrn CH1-CH2 mutants measured via Fluorescence Recovery After Photobleaching (FRAP) on stress fibers in HeLa cells. utrnΔN recovery and localization values from Harris et al[37]. Points are the mean values and error bars the standard error on the mean. (b) FRAP recovery curves from measurements on stress fibers. The black lines are the mean value at each given time point and the grey bars the standard error on the mean. The n numbers are individual bleaching regions from separate cells.

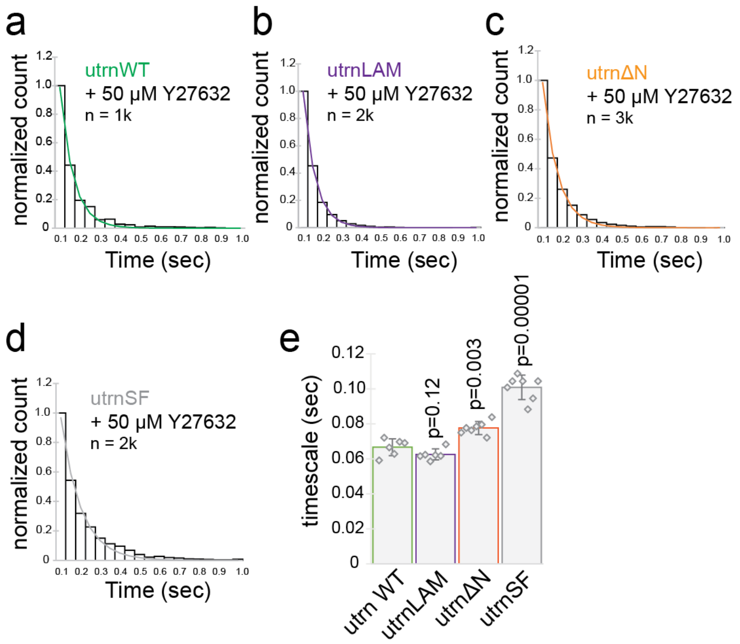

Figure S10: Kinetic measurements of utrn CH1-CH2 mutants in live cells

Single molecule dwell time histogram measured by photoactivation of utrophin mutant fusions to EOS in HeLa cells treated with 50µM Y27632 for (a) utrnWT, (b) utrnLAM, (c) utrnΔN and (d) utrnSF. The number of individual molecules analysed 'n' is shown in the figure inset. (e) Measurements of binding timescales for the different constructs evaluated from a minimum of 6 individual cells. Error bars are standard deviation. p values are calculated from a two-sided students t-test and assumed significant at $p<0.05$.
