# Supplementary material for: Biased localization of actin binding proteins by actin filament conformation
Source: Nat Commun. 2020 Nov 25;11:5973. doi: 10.1038/s41467-020-19768-9 (PMC7688639; doi:10.1038/s41467-020-19768-9)
Supplement: Supplementary file 10 — Reporting Summary [file 41467_2020_19768_MOESM10_ESM.pdf]

nature research

Corresponding author(s): Daniel A. Fletcher

Last updated by author(s): Oct 27, 2020

Reporting Summary

Nature Research wishes to improve the reproducibility of the work that we publish. This form provides structure for consistency and transparency in reporting. For further information on Nature Research policies, see Authors & Referees and the Editorial Policy Checklist.

Statistics

For all statistical analyses, confirm that the following items are present in the figure legend, table legend, main text, or Methods section.

n/a	Confirmed	
☐	☒	The exact sample size (*n*) for each experimental group/condition, given as a discrete number and unit of measurement
☐	☒	A statement on whether measurements were taken from distinct samples or whether the same sample was measured repeatedly
☐	☒	The statistical test(s) used AND whether they are one- or two-sided<br>*Only common tests should be described solely by name; describe more complex techniques in the Methods section.*
☒	☐	A description of all covariates tested
☐	☒	A description of any assumptions or corrections, such as tests of normality and adjustment for multiple comparisons
☐	☒	A full description of the statistical parameters including central tendency (e.g. means) or other basic estimates (e.g. regression coefficient) AND variation (e.g. standard deviation) or associated estimates of uncertainty (e.g. confidence intervals)
☐	☒	For null hypothesis testing, the test statistic (e.g. *F*, *t*, *r*) with confidence intervals, effect sizes, degrees of freedom and *P* value noted<br>*Give P values as exact values whenever suitable.*
☒	☐	For Bayesian analysis, information on the choice of priors and Markov chain Monte Carlo settings
☒	☐	For hierarchical and complex designs, identification of the appropriate level for tests and full reporting of outcomes
☐	☒	Estimates of effect sizes (e.g. Cohen's *d*, Pearson's *r*), indicating how they were calculated

Our web collection on statistics for biologists contains articles on many of the points above.

Software and code

Policy information about availability of computer code

Data collection: Microscope hardware was controlled using MicroManager 1.4. Fiji and MatLab 2020a were used for image analysis

Data analysis: Single particle events were tracked using TrackNTrace v2.0 (Stein and Thiart 2016) freely available software package for MatLab (2020a). Tracked events were analyzed in MatLab using a custom written routine. The illustration of actin filaments and binding proteins were generated using CellScape molecular visualization software developed by Jordi Silvestre-Ryan (https://github.com/jordisr/cellscape).

For manuscripts utilizing custom algorithms or software that are central to the research but not yet described in published literature, software must be made available to editors/reviewers.

Data

Policy information about availability of data

All manuscripts must include a data availability statement. This statement should provide the following information, where applicable:

- The following accession codes were used for generating illustrations of actin filaments and binding proteins in Figure 1 and Figure S5 (https://www.rcsb.org/). 1QAG, 6M5G, 6T20, 6T23, 6T1Y, 3J0S.

Single molecule CDF data and the MatLab code to analyze single molecules are available from the corresponding author upon reasonable request.

Field-specific reporting

Please select the one below that is the best fit for your research. If you are not sure, read the appropriate sections before making your selection.

☒ Life sciences ☐ Behavioural & social sciences ☐ Ecological, evolutionary & environmental sciences

Life sciences study design

All studies must disclose on these points even when the disclosure is negative.

Sample size	Sample sizes were chosen to bes 3 individual imaging chambers imaged on different days as technical replicates for single molecule measurements. Replicate consisted of >1000 events which could be used to generate statistically significant kinetic measurements
Data exclusions	No data were excluded from analysis
Replication	All attempts at replication were successful and technical replicates incorporated into the analysis
Randomization	Samples were randomly allocated into experimental groups
Blinding	The invesigators were blinded to group allocation during analysis of single molecule measurements. Data analysis was automated using MatLab

Reporting for specific materials, systems and methods

We require information from authors about some types of materials, experimental systems and methods used in many studies. Here, indicate whether each material, system or method listed is relevant to your study. If you are not sure if a list item applies to your research, read the appropriate section before selecting a response.

Materials & experimental systems

n/a	Involved in the study	
☒	☐	Antibodies
☐	☒	Eukaryotic cell lines
☒	☐	Palaeontology
☒	☐	Animals and other organisms
☒	☐	Human research participants
☒	☐	Clinical data

Methods

n/a	Involved in the study	
☒	☐	ChIP-seq
☒	☐	Flow cytometry
☒	☐	MRI-based neuroimaging

Eukaryotic cell lines

Policy information about cell lines

Cell line source(s)	HeLa and HEK293 cells were obtained from ATCC. PLB cells, an HL60 derivative (ATCC) were a gift from Sean Collins (UC Davis).
Authentication	none of the cell lines used were Authenticated
Mycoplasma contamination	All cell lines tested negative for mycoplasma contamination
Commonly misidentified lines (See ICLAC register)	No commonly misidentified cell lines were used in this study
