# Supplementary material for: Biased localization of actin binding proteins by actin filament conformation
Source: Nat Commun. 2020 Nov 25;11:5973. doi: 10.1038/s41467-020-19768-9 (PMC7688639; doi:10.1038/s41467-020-19768-9)
Supplement: Supplementary file 11 — Description of Additional Supplementary Files [file 41467_2020_19768_MOESM11_ESM.docx]

Description of additional supplementary information file

Title: Supplementary Movie 1

Description : HeLa cell expressing utrnWT (green) and utrnLAM (magenta)

Title: Supplementary Movie 2

Description : PLB cell expressing utrnWT (green) and utrnLAM (magenta)

Title: Supplementary Movie 3

Description : HeLa cell expressing utrnWT (green) and utrnΔN (magenta)

Title: Supplementary Movie 4

Description : PLB cell expressing utrnWT (green) and utrnΔN (magenta)

Title: Supplementary Movie 5

Description : HeLa cell expressing utrnWT (green) and BPAG1 ABD (magenta)

Title: Supplementary Movie 6

Description : HeLa cell expressing utrnWT (green) and NesprinII ABD (magenta)

Title: Supplementary Movie 7

Description : PLB cell expressing utrnWT (green) and NesprinII ABD (magenta)
