# Supplementary material for: Biased localization of actin binding proteins by actin filament conformation
Source: Nat Commun. 2020 Nov 25;11:5973. doi: 10.1038/s41467-020-19768-9 (PMC7688639; doi:10.1038/s41467-020-19768-9)
Supplement: Supplementary file 2 — Peer Review File [file 41467_2020_19768_MOESM2_ESM.pdf]

REVIEWER COMMENTS

Reviewer #1 (Remarks to the Author):

In this manuscript, Harris et al. address a very timely and interesting question: how do conformational changes in actin filaments, caused by a variety of factors, affect the binding of proteins to the filaments? The key experiments of this manuscript use different mutants of the actin-binding domain of utrophin (utrn), fluorescently labeled, to perform single molecule experiments. They provide a large numbers of events, to which a powerful analytical method is applied, allowing the authors to investigate the complex behavior (double exponential) of utrn mutants unbinding from the filaments.

However, the main claim of the manuscript, i.e. that the observed differences in utrn binding are due to conformational changes in the actin filaments, is not supported by the data. The experiments merely show that, in the presence of drugs/cofilin/drebrin/myosin, utrn binding is altered. A number of other interpretations could be made, and some could be tested by additional experiments, by a more thorough data analysis, and by basic control experiments. At this stage, I cannot recommend the publication of this manuscript.

I make the following specific comments with the hope that it will help the authors improve their manuscript. My comments focus on the in vitro experiments, on which the key points of the paper rest.

1. About the single molecule assay.

Controls and characterizations are missing. Most importantly, the contribution of photobleaching (not even mentioned) must be quantified and taken into account. This is crucial to ensure that the double exponential is not due to the observation of unbinding events plus bleaching events. Also, please provide more information about the filtered-out events corresponding to the binding of utrn to the surface, outside of the filaments (some of these events also take place within the filament pixels, and contribute as background).

To estimate the on-rates of Fig 2C, I assume that only one concentration was used for each utrn mutant (this is not specified). This is not enough. Different concentrations have to be used, and a linear regression must be reported.

The result of Figure 2D-I is extremely surprising: the utrn mutant K121A, which is closest to the WT (only one point mutation), differs more from the WT in its unbinding behavior, than utrn-LAM which has two additional point mutations (Q33A T36A K121A). How can that be?

2. Binding of small molecules

Changes in utrn kinetic parameters (mostly, dwell times), when filaments are also exposed to Jasplakinolide, are interpreted as being due to changes in filament conformation but there is no clear evidence of that. In the experiments, are filaments saturated with Jasplakinolide? Where does utrn bind, on the actin filament, compared to Jasplakinolide? Is a steric clash expected, or a change in electrostatic charge?

Do the side-decorating drugs (or, later, proteins) affect the anchoring of the filaments to the surface, via anchored phalloidin (which may contribute to changes in utrn kinetics, see point 6)? Soluble phalloidin likely competes with surface-attached phalloidin.

3. About cofilin and drebrin experiments

The global cofilin severing rate is a gross readout, and its decrease could be due to something else

than a direct competition for binding sites. In fact, a direct competition between cofilin and utrn for binding sites on the filament could even enhance severing. The proposition that a competition is at play could be tested easily by measuring utrn kinetics on cofilin-saturated filaments. Varying protein concentrations would also bring some insights.

When adding utrn after cofilin, it seems that cofilin clusters last for a while without severing the filament. Is that the case? Can the authors quantify the severing rate per cluster and compare it to reported values? Could some severing events go unnoticed because of the anchor density? How are severing events taken into account in the analysis?

Do cofilin clusters grow over time? If not, could it be that they stop growing when they reach a phalloidin anchor to the surface? Controlling the anchor density would also be very helpful, since the global severing rate depends on the density of anchoring points (see Pavlov et al. JMB 2007).

The utrn binding events taking place near the cofilin clusters may be partially on the cofilin clusters. Can this be considered, or ruled out? Here as well, data on cofilin-saturated filaments would help.

In Fig 4D, it would be useful to show the control situation (no cofilin), as a comparison.

With drebrin: are the filaments saturated? Are they exposed prior to utrn, or directly to both proteins at the same time? Do utrn mutants bind where drebrin is, or away from it?

4. Experiments with myosin

How does blebbistatin affect myosin binding to the filaments? In Fig 4D there seem to be a lot of actin filament bundles, are they due to blebbistatin? These bundles make the comparison with the images in Fig 4B difficult and unreliable. Perhaps the authors should have used an ATP-free situation rather than blebbistatin to turn off contraction (as in the recent preprints from the Alushin lab).

When comparing dwell-times, what is the control situation? Filament networks without myosin, but with alpha-actinin? Raw data of fluorescent spots should be provided, as for other experiments.

5. physical confinement

There is absolutely no evidence that the observed clustering is due to the anchoring points (there is no control, no variation of anchor density). The "clustering of dwell times" is observed because of a change in the observation method, not a change in the anchoring conditions.

If anchoring to the surface had an impact on utrn binding kinetics (and it might, indeed), that would put in question all of the previous results where filaments were also anchored to the surface.

6. The experiments are not sufficiently described, and basic information is missing.

The reader needs to get an idea of how each experiment is done, without going back and forth to the Methods section. And the Methods section does not always help. For example, the description of the "actin network assay" is very unclear. After reading it several times, I still have no idea how the experiment is done.

All protein concentrations should be indicated in the figures or their caption (not just in the Methods).

Most scales bars have no numbers, and are not specified in the caption. There are no scale bars for distances in kymographs.

The only information about cofilin is that it was "a kind gift from Peter Bieling" (in methods). How is it

labeled? What isoform is it? From what organism?

The composition of F-buffer is not given. The buffer used for experiments is never specified, except "AB buffer" for which the pH is unknown.

I am sorry to have to say this, but so much information is missing that I often wondered how many times the authors had read their manuscript before submitting it.

7. Minor points

In Fig 1 the purple residues are not easy to see.

Missing panel in Fig S3, and wrong letters (E, F) in its caption.

At least twice, the text seems to imply that severing scales with cofilin concentrations. It does not. It scales with the number of cofilin cluster boundaries, as indicated in the introduction.

Lots of letter are not capitalized, while it seems that, by convention, they usually are: f-actin (F-actin), g-actin (G-actin), n-terminal (N-terminal).

Fig 3D and 4H, y-axis: "fraction (tau1 to tau2)" seems incorrect. This is not a ratio of times, is it? Rather, it is "a1", referred to earlier as "relative amplitude of the two timescales", and which actually seems to be the fraction of unbinding events that belong to the fast population. Is that correct?

Several typos, missing or extra words. (e.g., bottom of page 10, "we measured generated…") and inaccuracies (e.g., top of page 13, a "dwell time" is a number, it cannot be "longer lived")

Sample size should be indicated in figures (in particular, when plotting a CDF).

Reviewer #2 (Remarks to the Author):

In the manuscript "Biased localization of actin binding proteins by actin filament conformation" Harris et al. present abundant binding data of different actin-interacting proteins and show that these are influenced by the actin structure. In general, I find the results very interesting and useful. However, I have a technical comment on the kSTORM measurements, as well as some minor comments.
Major comment, kSTORM analysis, Fig. 6: The idea to look at spatial variability of kinetic constants by kSTORM is certainly very interesting. However, I am not convinced that the results in Fig. C-E show more than noise.
a) The on-times that are plotted here follow an exponential distribution. Thus, in each pixel many binding events need to be detected to estimate the mean with a high accuracy. This information needs to be presented (e.g. a second image showing the counts per pixel). Also, consider taking longer measurements.
b) From the counts per pixel, please estimate the statistical error in t and compare it with the variation along the fibril to show that these are not statistical in nature but denote real variation.
c) A control I would like to see is to divide the data set into two sets and to plot the t value in the first vs the t value in the second half for each pixel in a scatter plot. Only if there is a strong correlation, we can interpret the actual t values in the pixels as being more than noise.
d) Please explain the Omega analysis better and motivate why it is applicable here (there are many algorithms that quantify clustering in images, why take an approach from genomics? What does it really measure?).
e) Is the interpretation based only on the shown figures? Or on how many figures? More statistical analysis is needed to demonstrate that indeed C-E show different degrees of clustering, including the

mean +/- SEM of the clustering metric for the different conditions and number of experiments.

Minor comments

1. The quantitative measurements of the two kinetic contestants for different mutants and proteins could be very useful for other work, including simulations. To exclude that photo bleaching has an effect on the values (especially the long time constant) I would like to see additional controls. Either taking the same measurements at different intensities to test from which intensity value a change in the kinetic parameters occurs, or an independent measurement of photo bleaching times to demonstrate that they are much longer than the slow time scales.
2. All plots showing the 1-CDF. The y-axis is a bit confusing. I assume it is in logarithmic units? Then please point this out and add a few more values to make it clear. Also, for comparison, it would be great to scale all 1-CDF plots the same way with the y-axis ranging from 0.02 to 1.
3. Fig. 3A. The authors write that phalloidin has no effect on utm wt or LAM, but that Jasp does. Looking at the graphs I don't agree with the interpretation. If a result is not significant, it does not mean there is no effect. The change in t1 is about 1/3 as big for phalloidin as for Jasp, so there seems to be an effect, however a weaker one for phalloidin compared to Jasp.
4. Fig. 4 D, Fig. S4. I am not entirely convinced that the results are not just due to statistical variability. Reporting the confidence intervals of fits is different to looking at actual variations of results (as is done for all the rest of the manuscript). I would prefer fitting individual experiments individually. If this is not robust, consider fixing t2 and its amplitude (same for far vs near) to show that there is indeed a significant change in t1. Or take more data.
5. Figure S3: F is missing.

Response to Reviewers

"Biased localization of actin binding proteins by actin filament conformation" Harris, et. al.

We thank the Reviewers for their careful consideration of our manuscript. We appreciate the helpful comments and constructive feedback, as well as the suggestions of new experiments and changes to the manuscript where we unintentionally left out information. In this document, we discuss each point raised by the Reviewers and describe the actions we have taken to address them. In particular, we have added a significant number of new experiments, controls and analysis to the manuscript as well as additional details to clarify our experiments. Thanks to the modifications and additional data collected based on Reviewer suggestions, we believe the manuscript is significantly improved.

Below please find our Point-by-Point Response to the Reviewers comments:

Reviewer #1 (Remarks to the Author):

In this manuscript, Harris et al. address a very timely and interesting question: how do conformational changes in actin filaments, caused by a variety of factors, affect the binding of proteins to the filaments? The key experiments of this manuscript use different mutants of the actin-binding domain of utrophin (utrn), fluorescently labeled, to perform single molecule experiments. They provide a large numbers of events, to which a powerful analytical method is applied, allowing the authors to investigate the complex behavior (double exponential) of utrn mutants unbinding from the filaments.

However, the main claim of the manuscript, i.e. that the observed differences in utrn binding are due to conformational changes in the actin filaments, is not supported by the data. The experiments merely show that, in the presence of drugs/cofilin/drebrin/myosin, utrn binding is altered. A number of other interpretations could be made, and some could be tested by additional experiments, by a more thorough data analysis, and by basic control experiments. At this stage, I cannot recommend the publication of this manuscript.

We appreciate the Reviewers positive comments about the work and suggestions for additional experiments to test our interpretations. We have now added new control experiments and analysis to support our conclusions, which are detailed below and included in the manuscript. Of particular note, we carried out additional kSTORM experiments imaging filaments attached to surfaces with different mechanical characteristics to explore in more detail the impact of physical confinement on ABD binding. We also measured the CDF of dwell times on filaments polymerized under different conditions which can result in filaments with different structural conformations. These experiments more directly confirm the role of actin filament conformation on ABD binding.

I make the following specific comments with the hope that it will help the authors improve their manuscript. My comments focus on the in vitro experiments, on which the key points of the paper rest.

1. About the single molecule assay.

Controls and characterizations are missing. Most importantly, the contribution of photobleaching (not even mentioned) must be quantified and taken into account. This is

crucial to ensure that the double exponential is not due to the observation of unbinding events plus bleaching events. Also, please provide more information about the filtered-out events corresponding to the binding of utrn to the surface, outside of the filaments (some of these events also take place within the filament pixels, and contribute as background).

We thank the reviewer for pointing this out and apologize for the omission. We have now incorporated the contribution of photobleaching into our analysis. We characterized bleaching in our experiments in two different ways.

(1) Firstly, we measured the bleaching rate of Streptavidin conjugated to Alexa555 attached to our biotinylated glass coverslips (Response Figure 1A). Alexa555 is the same dye we use to measure actin binding domain kinetics. The high affinity of the Biotin-Streptavidin interaction (~10^{-14} M) means that loss in fluorescence molecules during imaging is dominated by bleaching and allows us to characterize the overall bleaching rate. We used the same imaging conditions as for our single molecule experiments including the laser power (~7mW) and exposure time (130ms per frame, 600 frames). The bleaching time constant we measured was 5.6 ± 0.1 seconds. We note that this is approximately double that of the second timescale (longer) of the utrophin mutants that we measured (utrnWT = 3.2 ± 0.3 sec, utrnLAM = 3.0 ± 0.1 sec and utrnN = 2.3 sec ± 0.1 sec).

(2) Secondly, we repeated the single molecule utrnWT binding measurement with a range of different laser powers to examine potential changes in the second timescale, as suggested by Reviewer 2 (Response Figure 1B). We observed little change in the second timescale between 30% and 40% power settings (~7 mW and ~10 mW measured out of the objective), though the measured second timescale did begin to change significantly (τ_2 = 2.17 ± 0.4 sec, a 32% shorter) at double the laser intensity (60% power ~21mW).

Based on these measurements we can conclude that the double timescale response that we observe is not due to photobleaching, since (1) the photobleaching time is longer than our measured second timescale and (2) we use laser powers <10 mW. However, to account for the photobleaching rate in our measurements, we added a correction to the measured timescales for each actin binding domain based on the approach of Hayakawa et al[1]. The bleaching rate can be subtracted from the measured off-rate to provide a corrected off-rate (inverse of the dwell time), where $\frac{1}{\tau corr} = \frac{1}{\tau measured} - \frac{1}{\tau bleach}$. In the manuscript (Figures 2-6), we now report values in bar charts for both τ_1 and τ_2 as bleaching corrected dwell times. For cumulative distribution function plots (Figure 2 and Supplementary Figures 4,6,7), we left these as the raw collected data.

The bleaching measurements have been added to the manuscript in the supplementary information (Supplementary Figure 4). We have added additional details both to the materials and methods (see “single molecule analysis” and figure captions to clarify this point.

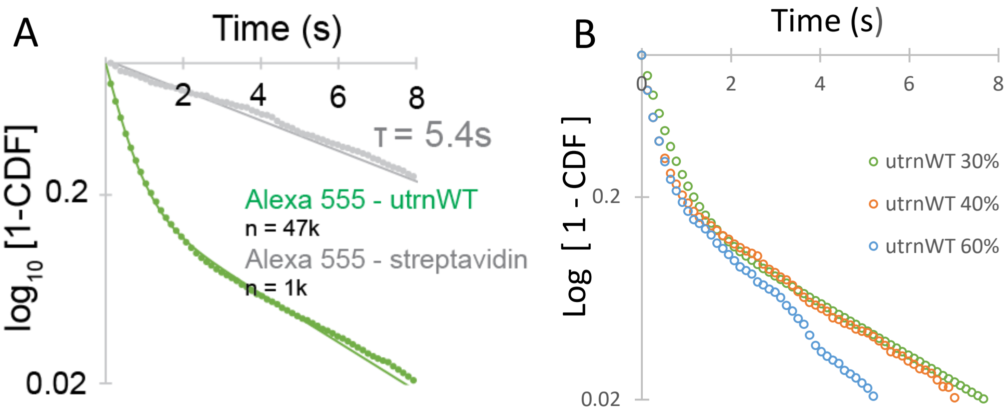

Response Figure 1: *(A) Measurement of the photobleaching rate of alexa555-streptavidin. (B) Measurement of the CDF of utrnWT imaged with different laser powers. 30%, 7mW shown in green, 40% 10mW shown in orange, 60% 21mW shown in blue.*

Regarding the Reviewer's second point about filtered-out events, we have updated the methods describing how single molecule images are analyzed so that they are more comprehensive, as described below (see "single molecule analysis" in the Methods). We have also added a supplementary figure (Supplementary Figure 3) to describe the single molecule analysis pipeline (shown below).

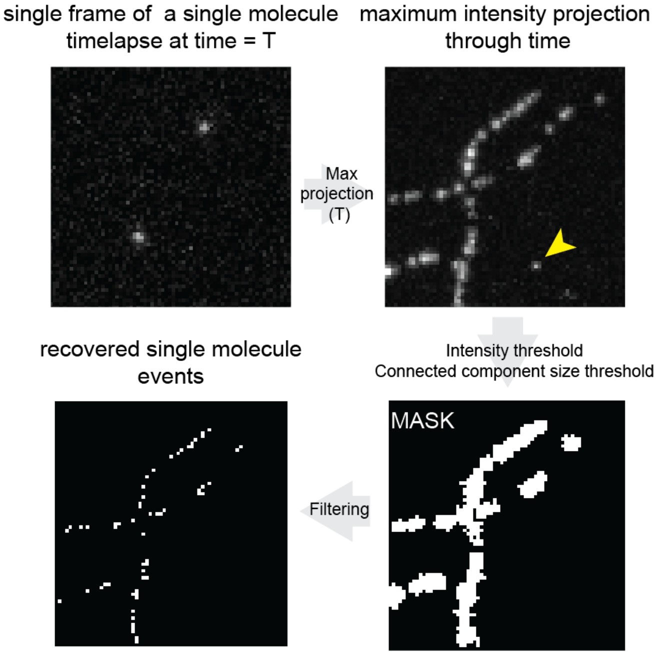

Response Figure 2: *Analysis pipeline for single molecule images.*

We filtered out events unrelated to filament binding by generating a mask based on maximum intensity projection (MIP) through time of the single molecule timelapse image. The mask was created by thresholding the MIP using Otsu's method followed by image closure of the mask (morphological erosion followed by dilation) and removal of objects

within the masked image that were smaller than 5 pixels. Objects of 5 pixels or less represent isolated binding events in the mask. Single molecule binding events occurring outside of the masked pixels were then removed from the analysis. However, the number of events removed corresponded to a small fraction of the total events (<1%). In general, non-specific events were rare, due to rigorous passivation of the glass surface with a PEG monolayer and additional orthogonal blocking with β-casein. However, as the reviewer points out, a small number of events could occur within the filament and contribute background to the measurements. We have noted this possibility in the methods (see “single molecule analysis” in the methods).

To estimate the on-rates of Fig 2C, I assume that only one concentration was used for each utrn mutant (this is not specified). This is not enough. Different concentrations have to be used, and a linear regression must be reported.

We agree with the Reviewer. As suggested, we have repeated the on-rate measurements as for a minimum of three different concentrations for each of the actin binding domain constructs that we have tested. We measured the frequency of binding events over a broad range of concentrations (0.05nM – 1nM), while retaining single molecule resolution (Response Figure 3).

Our results show that the on-rate of utrnWT, utrnLAM and utrnΔ were similar, with utrnSF having a higher on-rate. (Kon utrnWT = 2.2 ± 0.3 $\mu M^{-1}s^{-1}$, kon utrnLAM = 2.4 ± 0.3 $\mu M^{-1}s^{-1}$, kon utrnΔnterm = 2.1 ± 0.3 $\mu M^{-1}s^{-1}$, kon utrnSF = 5.0 ± 2.0 $\mu M^{-1}s^{-1}$).

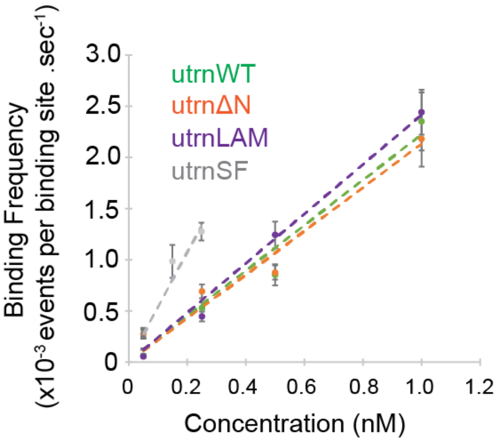

k_{on} utrnWT = 2.2 ± 0.3 μM^{-1} s^{-1}
k_{on} utrnLAM = 2.4 ± 0.3 μM^{-1} s^{-1}
k_{on} utrnΔN = 2.1 ± 0.4 μM^{-1} s^{-1}
k_{on} utrnSF = 5.0 ± 2.0 μM^{-1} s^{-1}

Response Figure 3: *Measurement of binding frequency for a range of different concentrations of actin binding domain in solution. Linear fits of binding frequency versus concentration yield the on-rate of binding. This was similar for the different constructs we investigated, aside from utrnSF which had an ~2 fold higher on-rate.*

The result of Figure 2D-I is extremely surprising: the utrn mutant K121A, which is closest to the WT (only one point mutation), differs more from the WT in its unbinding behavior, than utrn-LAM which has two additional point mutations (Q33A T36A K121A). How can that be?

Exactly! This is an important point highlighted by the Reviewer related to an interesting feature of CH1-CH2 domains, which we believe contributes to the conformational sensitivity we are reporting in this work. There are two mechanisms that govern the affinity of CH1-CH2 domain binding to actin.

(1) Firstly, inter-CH domain interactions are important for controlling binding affinity, as they are involved in controlling the overall conformation of the domain. In our previous work[2], we showed that mutations at the interface between CH1 and CH2 (in this case Q33A T36A) disrupt interdomain interactions. This allows the two CH domains to more easily separate from one another (which is commonly referred to as 'opening'[3]) and adopt a high affinity actin binding state. The mechanism underlying this behavior results from CH1 and CH2 having separate roles in actin binding. CH2 sterically interacts with F-actin and serves as a negative regulator of binding, while CH1 directly interfaces with F-actin and therefore favors actin binding. Opening of CH1-CH2 relieves the steric interaction between CH2 and F-actin, causing an increase in binding affinity. Mutations introduced at the interface between CH1 and CH2 (ie Q33A T36A) serve as a way to increase the overall actin binding affinity of the domain without changing the residues on CH1 that directly interact with F-actin (Response Figure 4A).

(2) Secondly, residues on CH1 make direct interactions with F-actin, and mutations to these residues reduce actin binding affinity and presumably change interactions with different monomers within an actin filament (Response Figure 4B). The single mutant K121A affects a direct interaction with F-actin[4,5] and hence reduces actin binding affinity and, we find, changes the specificity for different conformational states.

These two mechanisms for modulating affinity – changing steric interference by CH2 and changing the binding surface of CH1 to f-actin – are independent for many residues in the domain, and hence can be combined to create distinct properties. For example, adding Q33A T36A to the K121A mutant recovers the overall actin binding affinity of the WT domain by increasing the openness of the domain. Interestingly, it retains the same specificity for different conformational states of F-actin of the K121A mutant alone, as these interfacial residues are not involved in direct interactions with F-actin[2,3]. The mutant Q33A T36A K121A therefore has a similar overall binding affinity to utrnWT but a difference in its specificity to actin conformation. Fundamentally, it is the combination of these two mechanisms that enables us to investigate the different roles of affinity and actin filament conformational sensing by CH1-CH2 domains.

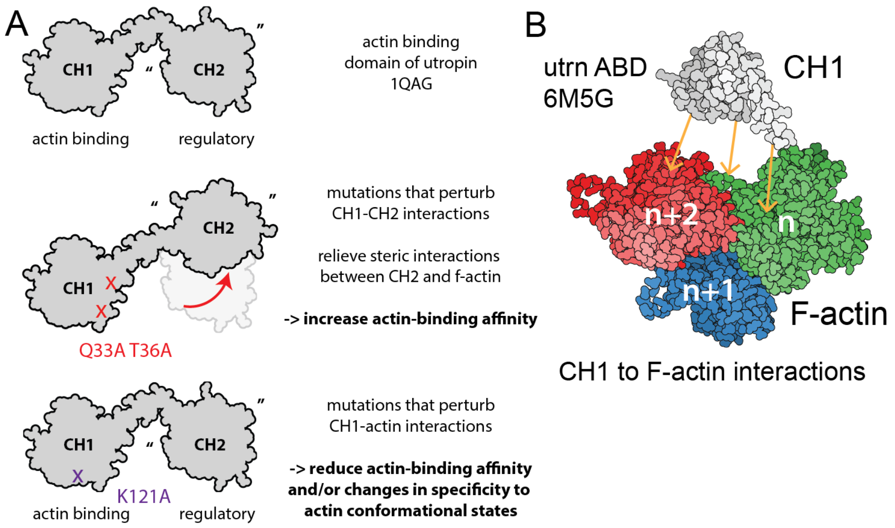

Response Figure 4: *Two mechanisms regulate the binding affinity of CH1-CH2 domains to F-actin. (A) Mutations at the interface between CH1 and CH2 change the ability of the domain to adopt an open conformation. This relives a steric clash between CH2 and F-actin resulting in increased F-actin binding affinity. (B) Mutations that target direct interactions between CH1 and F-actin reduce actin binding affinity. CH1 makes contacts on and in-between longitudinally adjacent monomers on the actin filament which is a possible mechanism for CH1-CH2 conformational sensing.*

To improve the clarity for this point in the manuscript, we have moved a description of these two mechanisms from the methods to the beginning of the results section. We have also added illustrations based on recently published cryo-EM structures of the actin binding domain of utrophin bound to F-actin to further clarify this point[5].

2. Binding of small molecules

Changes in utrn kinetic parameters (mostly, dwell times), when filaments are also exposed to Jasplakinolide, are interpreted as being due to changes in filament conformation but there is no clear evidence of that. In the experiments, are filaments saturated with Jasplakinolide? Where does utrn bind, on the actin filament, compared to Jasplakinolide? Is a steric clash expected, or a change in electrostatic charge?

We appreciate the concern of the Reviewer and we have added new experiments to address this point. Cryo-EM structures of phalloidin stabilized actin and jasplakinolide stabilized actin have recently been published that confirm differences in filament conformation[6]. We have added illustrations based on these crystal structures to clarify where these small molecules bind and how this changes the conformation of actin filaments[6].

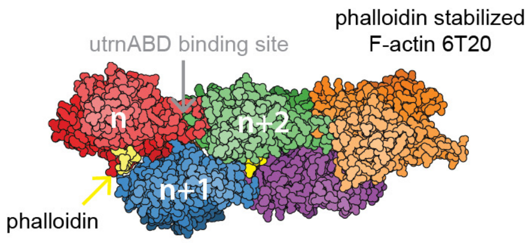

Response Figure 5: *Locations of the phalloidin and utrophin binding sites on F-actin.*

As shown in Response Figure 5, phalloidin and jasplakinolide occupy very similar binding sites on F-actin. Utrn ABD's have a completely distinct binding site on F-actin, interacting with the D-loop between adjacent monomers, which was also seen in a recent structural study[5]. We therefore do not expect a steric interaction to arise from the presence of these small molecules. We can also see in the structures that these two small molecules have a distinct allosteric effect on the conformation of the D-loop of subdomain 2 on F-actin, which is part of the utrophin ABD actin binding site (Response Figure 4B, Response Figure 5). Therefore, it is plausible that structural changes to this region could impact utrophin ABD mutants in different ways, in particular when the mutations interact directly with the D-loop.

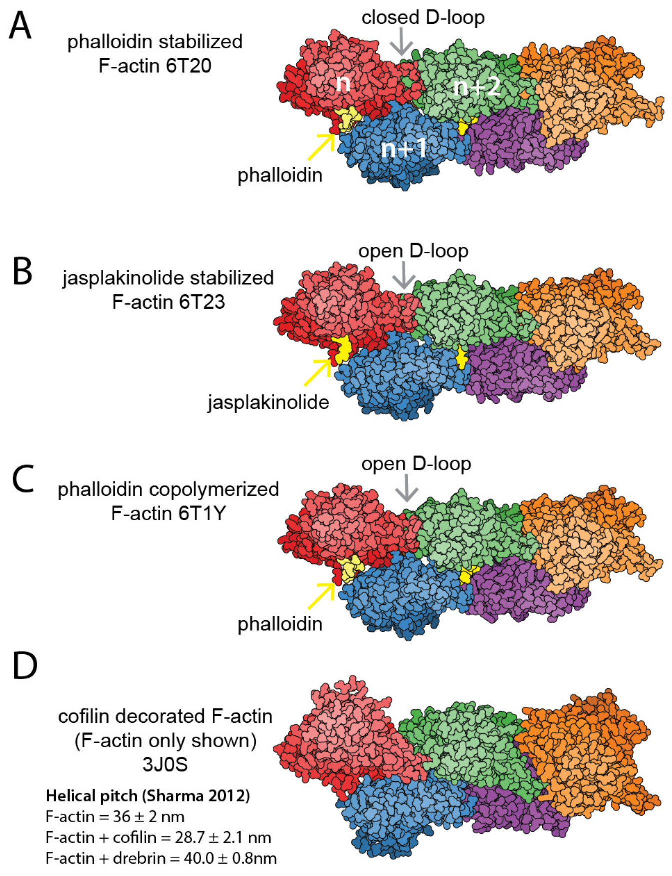

Response Figure 6: *(A) The actin binding domain of utrophin CH1 interacts with the D-loop on F-actin and makes contact with longitudinally adjacent actin monomers within the*

filament. Jasplakinolide and Phalloidin have a similar binding site to each other on F-actin which is distinct from the utrophin CH1 binding site. The small molecules Jasplakinolide (B) and Phalloidin (A) stabilize different D-loop conformations when added to actin filaments post polymerization. Interestingly, actin filaments polymerized in the presence of phalloidin (C) have a D-loop conformation that is similar to jasplakinolide stabilized filaments. The same small molecule can therefore be used to generate different actin filament conformations. (D) The binding proteins cofilin and drebrin also change actin filament conformation and alter the helical half pitch of the filament. The images shown here are taken from structural data with the PDB code shown next to the actin filament image.

Interestingly, it was shown in the same manuscript[6] that actin polymerized in the presence of phalloidin (as opposed to stabilized with phalloidin post polymerization) results in a structure that is very similar to actin bound by jasplakinolide (Response Figure 6). If our utrophin ABD mutants are indeed sensitive to conformational changes in F-actin rather than interactions with the different drugs, then we should see changes in dwell time on filaments polymerized in the presence of phalloidin and those stabilized by phalloidin after polymerization.

So, we tested the dwell times of the different utrophin actin binding domain mutants to actin that had been polymerized in the presence of biotinylated phalloidin, which we use for anchoring filaments to the coverslip. Interestingly we observed a very similar response of the utrophin ABD mutants to jasplakinolide, with a dwell time τ_1 that was indistinguishable for the two conditions. In Response Figure 6, we show CDFs for jasplakinoldie filaments (black, top row) and actin filaments co-polymerized with phalloidin (red, bottom row) and the fitted timescale for τ_1 shown in Response Figure 7. The effects of filament conformation on utrn ABD binding is similar for these two conditions, but it is different for filaments polymerized and then subsequently stabilized with phalloidin (grey, top row).

This result highlights that different conformational changes in F-actin introduced by the same stabilizing agent (with different polymerization conditions) can impact the binding of utrophin mutants. We have added this new data to the manuscript as Figure 3 and Supplementary Figure 6.

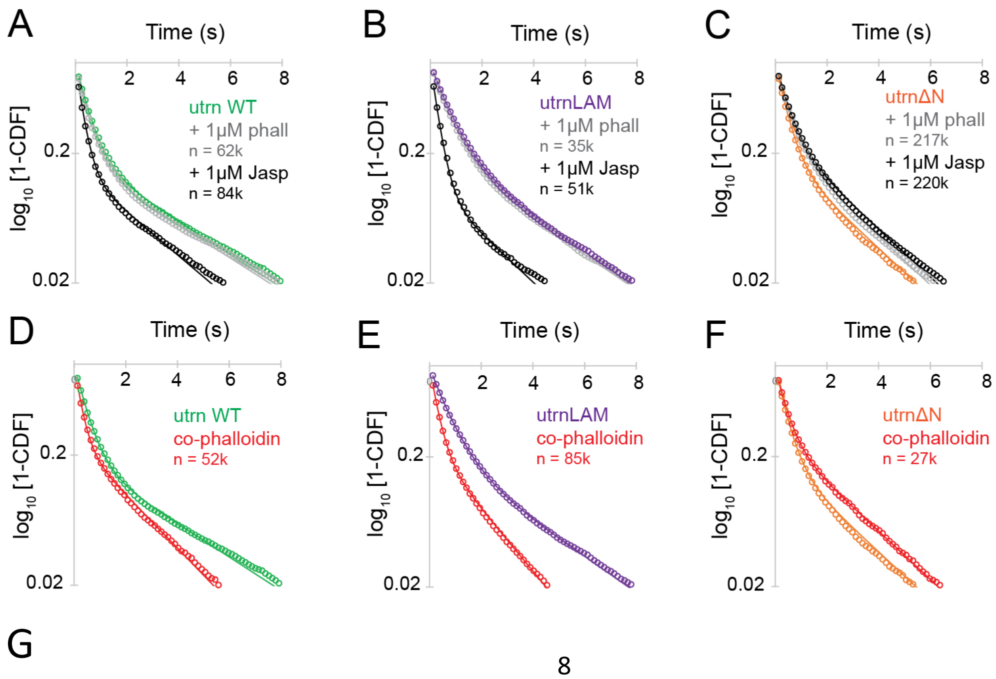

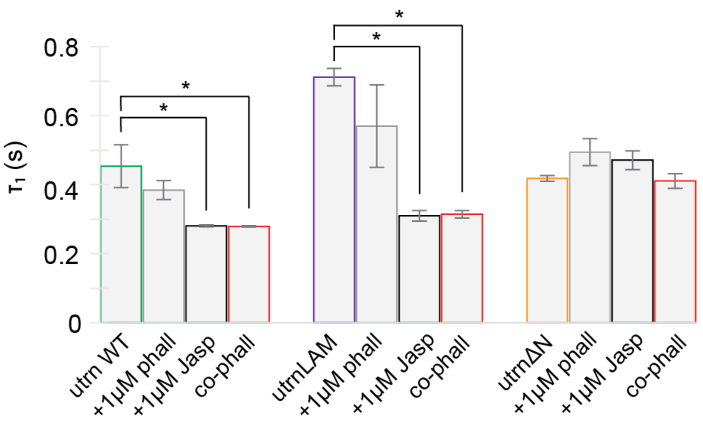

Response Figure 7*: (A-F) Cumulative distribution functions measured for the utrophin mutants binding to actin filaments stabilized in different conditions. (G)The timescales τ_1 measured by fitting the CDFs.*

To answer the Reviewer's comment related to the decoration of actin filaments with different proteins and stabilizing agents, we compare the reported k_d of the different molecules in our assay together with the abundance of actin filaments and the concentrations at which we add these molecules are added. We estimate from the imaging chamber volume and length of filaments on the surface in a typical field of view that we have a concentration of F-actin of ~50nM on the surface of the coverslip. All actin binding proteins (except for cofilin) are added at a concentration of >>50nM. The k_d of phalloidin is reported to be ~70-270nM[7] and jasplakinolide reported to be 15-300nM[8]. Since both of these reagents are added at a concentration higher than their k_d and we expect 100% labelling in these conditions.

Do the side-decorating drugs (or, later, proteins) affect the anchoring of the filaments to the surface, via anchored phalloidin (which may contribute to changes in utrn kinetics, see point 6)? Soluble phalloidin likely competes with surface-attached phalloidin.

This is an insightful point raised by the Reviewer. Indeed, soluble phalloidin will compete for binding sites with the surface anchored phalloidin. We cannot stabilize filaments with excess phalloidin (that is not biotinylated) and then attach them to the surface of the flow chamber that has been functionalized with biotin phalloidin, since the phalloidin binding site is already saturated. That is why soluble phalloidin and other proteins are only added after filaments are already tethered to the surface of the flow chamber. Because phalloidin has a high affinity for F-actin ~70-270nM, the tethering to the surface is strong and unbinds with a very slow rate. This means that examining the effect of different proteins and small molecules is somewhat distinguishable from examining differences in tethering, though it is difficult to ever decouple these effects entirely in any assay. We think that it is very interesting to consider how the combination of biochemical and physical conditions could change actin filament conformation, especially in a cellular context, where it is likely that a combination of these different mechanisms is at play.

We investigated further to what extent adding soluble small molecules might impact actin filament tethering in our assay. We added a range of different concentrations (0.1μM, 1μM and 5μM) of alexa-488 phalloidin after filaments have been tethered to the surface of the flow chamber. We found that we did not lose filaments from the surface of the flow chamber, as would be expected if the addition of soluble phalloidin dramatically changed tethering to

the surface. This is due to the high affinity of phalloidin for actin as well as the fact that filaments tethered to the chamber are already bound in those locations by biotin phalloidin on the surface. In Response Figure 8, we show images for 100nM, 1μM and 5μM phalloidin added after filaments are tethered to the surface (the same region of filaments has been imaged here for clarity).

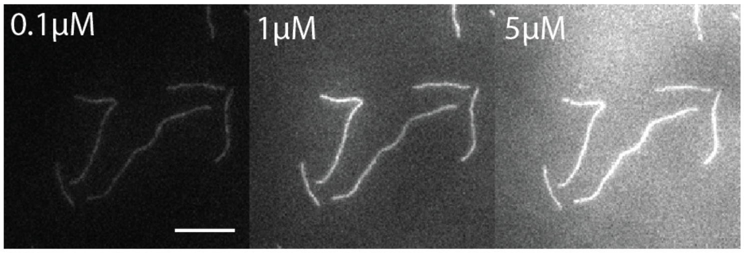

Response Figure 8: *From left to right, actin filaments labelled with 100nM, 1μM and 5μM fluorescently labelled phalloidin, which is added after filaments have been anchored to the surface of the flow chamber. The same filaments can be seen here in these images, indicating that filaments have remained attached, even in the presence of high concentrations of soluble phalloidin added. This feature is consistent at randomly chosen locations on the surface of the chamber and we see the same average amount of filaments on the surface, irrespective of the amount of soluble phalloidin added. The scale bar is 5μm.*

In addition, we measured the average abundance of filaments on the surface of the flow chamber in these different conditions for randomly chosen locations on the surface (Response Figure 9). Together, the filament imaging and quantification indicate that drug concentration does not have a significant effect on filament binding in our experiments.

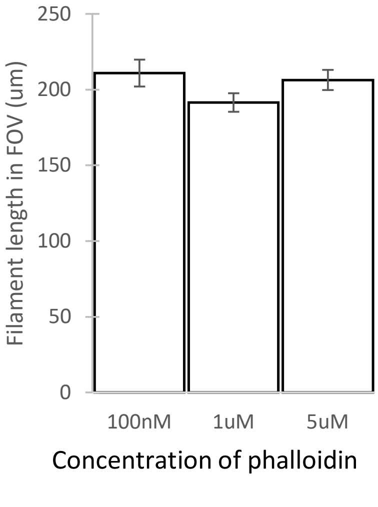

Response Figure 9: *Filament length in the Field Of View (FOV) for randomly selected regions after the addition of soluble phalloidin to the assay chamber. No loss of filaments was observed with the addition of phalloidin after filaments had already been tethered to the surface.*

In contrast to the potential effect of phalloidin, we do not expect addition of the side-binding proteins used in this study (utrnABD, myosin, cofilin and drebrin) would cause detachment of actin filaments from the surface since they have different binding sites from phalloidin and therefore should not disrupt tethering. Indeed, tethering to the surface via phalloidin is therefore highly desirable in our assay, as the high affinity of this interaction means that we do not lose filaments when additional agents are added. This tethering strategy is favorable for our measurements over other commonly used approaches, such as surface immobilized binding proteins (HMM, gelsolin etc), as these would compete for binding with utrophin ABD and the addition of high concentrations of the ABD would likely cause filament detachment.

3. About cofilin and drebrin experiments

We thank the Reviewer for the comments below about the drebrin and cofilin experiments. Before we address each point raised by the Reviewer and describe the additional experiments and controls we carried out, we have two overall responses:

(1) Firstly, previous work has shown that drebrin, cofilin and the actin binding domain from α-actinin (a CH1-CH2 domain) occupy similar binding sites on F-actin[9] and have interactions with subdomain 2 on F-actin. It is therefore likely that there would be competitive binding between these different proteins, as they share an overlapping binding site (in contrast to phalloidin which has a distinct binding site). Indeed, competitive binding exists between cofilin and drebrin, and drebrin "inhibits, but does not abolish cofilin-induced severing of actin filaments"[10]. The actin binding domain from alpha catenin, which shares a similar binding site on F-actin with utrophin ABD, also competes with cofilin and slows actin filament severing[11].

(2) Secondly, in response to the Reviewer's suggestion to use cofilin saturated filaments in a number of experiments, we tested this using high concentrations of cofilin and at different pH to generate cofilin saturated filaments in our assay. However, we were unable to generate cofilin saturated filaments that were sufficiently stable in our tethered-filament assay to carry out ABD binding experiments. As the reviewer points, mechanical heterogeneity causes filaments to be severed by cofilin, so we would need to change tethering conditions to minimize stresses introduced by tethering to avoid severing, which would make the experiments difficult to compare. We chose to keep the tethering conditions consistent among each experiment so that direct comparisons can be made, but that precludes use of cofilin saturated filaments in our specific assay.

Overall, we agree that it would be interesting to further investigate how the activity of different actin binding domains feedback on other regulatory proteins such as cofilin. While we have added new experiments and controls to support the results we present in the manuscript, we believe it is best to hold more extensive experiments for a future study focused on those proteins.

The global cofilin severing rate is a gross readout, and its decrease could be due to something else than a direct competition for binding sites. In fact, a direct competition between cofilin and utrn for binding sites on the filament could even enhance severing. The proposition that a competition is at play could be tested easily by measuring utrn kinetics on cofilin-saturated filaments. Varying protein concentrations would also bring some insights.

We agree with the Reviewer that the interactions with cofilin are of great interest and could be the basis for a future study. Indeed, we wanted to test if the severing rate was different – enhanced or reduced – in the presence of different actin binding domains. However, as

described above, we were unable to generate cofilin-saturated filaments in our assay where mechanical heterogeneity is introduced on the filaments by anchoring with biotin-phalloidin.

Instead, we have followed the second suggestion of the Reviewer to vary binding protein concentrations in the assay and test the impact on cofilin-mediated severing. We also added the no-cofilin control which is a subsequent suggestion of the reviewer below (Response Figure 10).

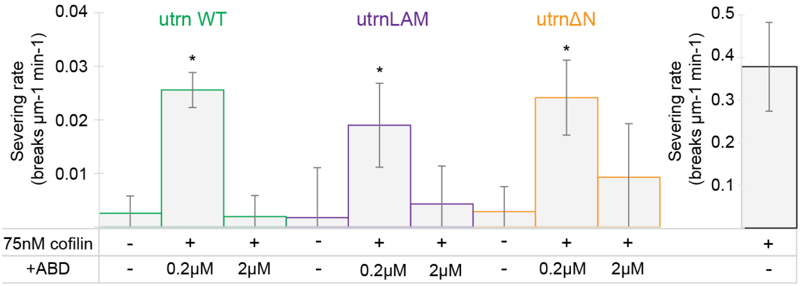

Response Figure 10: *Severing rate of cofilin measured in the presence of different concentrations of actin binding domain mutants. At high concentrations of the actin binding domain the rate of severing is slowed.*

From these results we can conclude that the presence of the actin binding domains in our assay significantly slows the rate of severing in an actin binding domain concentration dependent manner. We reason that this arises from a competition for binding sites and from the difference in on-rate of these two proteins, given that they are introduced into the assay at the same time. The on-rate for cofilin is reported to be 0.06 $\mu M^{-1}s^{-1}$ [1]. We have found that the utrophin mutants have a higher on-rate than cofilin, approximately 2 $\mu M^{-1}s^{-1}$. Therefore, we speculate that the utrophin actin binding domains bind rapidly in the assay and slow the formation of cofilin-bare actin boundaries and the growth of cofilin clusters through direct competition for binding sites and reduce the rate of actin filament severing.

When adding utrn after cofilin, it seems that cofilin clusters last for a while without severing the filament. Is that the case? Can the authors quantify the severing rate per cluster and compare it to reported values? Could some severing events go unnoticed because of the anchor density? How are severing events taken into account in the analysis?

We thank the Reviewer for these questions and realize that additional clarification is needed for how the experiments are performed. There are two key experiments using cofilin: (1) measurement of bulk actin filament severing and (2) measurement of single molecule dwell times near and far from cofilin clusters.

(1) In our bulk assay, the severing rates that we report (breaks μm^{-1} min^{-1}) are similar to previously reported values (~0.01 breaks $\mu m^{-1} min^{-1}$ in the presence of 1μM α-catenin actin binding domain[9]). In both assays, cofilin and other actin binding domains are introduced at the same time. Since the utrophin actin binding domains have a faster on-rate than cofilin, we see decoration of actin filaments first with the ABDs. We have updated the manuscript text to reflect this point.

(2) In the single molecule assay, both cofilin and actin binding domains are added at low concentration and hence can bind to actin that is undecorated. Both proteins are still added at the same time. The Reviewer raises an interesting question regarding severing events in the single molecule assay. Because filaments are not completely labelled at any one timepoint it is not possible to visualize the filament backbone and detect severing events directly. We therefore do not consider severing events into our analysis. To address this, we have added two additional analyses here. Firstly, we wanted to quantify what we describe as cofilin clusters in more detail and performed an intensity analysis on the cofilin spots in our images (shown below).

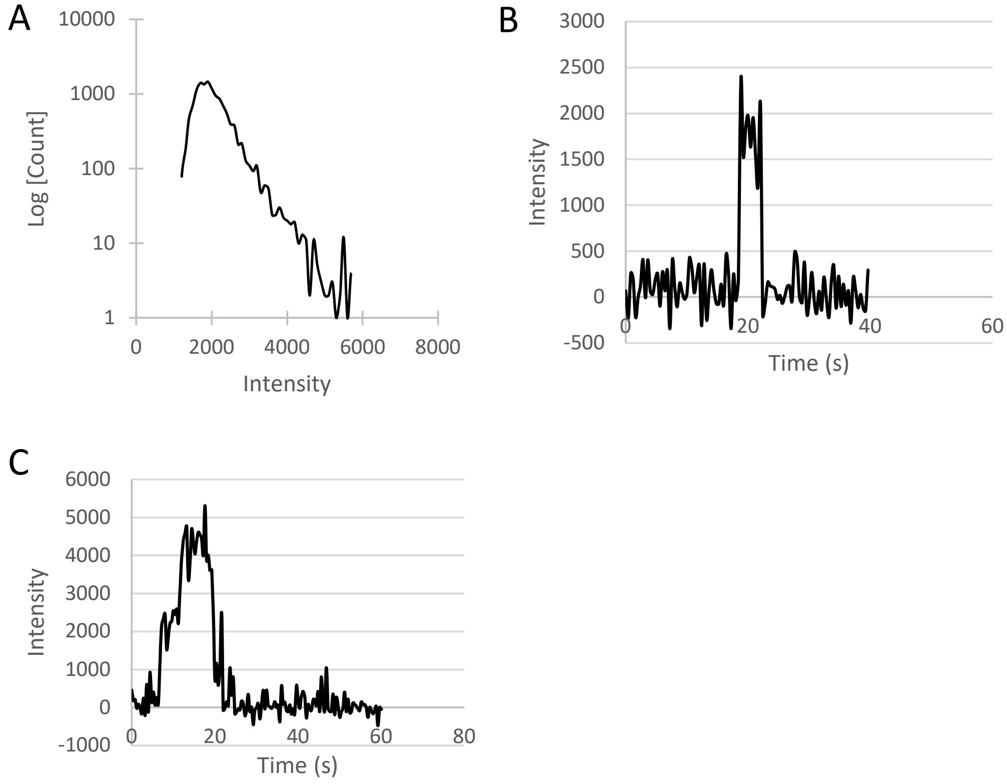

Response Figure 11: *Single molecule analysis of cofilin binding. (A) Number of events (counts) having a particular intensity, the y-axis is Log scale. The peak intensity measured was ~2000. (B) A single binding event which occurs in a single step has an intensity of ~2000, indicating that the vast majority of cofilin events are single molecule in our assay. (C) Some events occur in multiple steps showing that clusters can grow in our assay but corresponded to less than ~10% of total events measured.*

The above plot shows the abundance of cofilin binding events with different intensities (Response Figure 11). Here we can see that the majority of cofilin events that we observed have an intensity of ~2000 (Response Figure 11A). The high abundance of these events suggests that a large fraction of observed events represent single cofilin molecules. This is confirmed when analyzing the intensity of an event in time which occurs in a single step with an intensity ~2000 (Response Figure 11B). However, in some cases we also observe intensities up to ~4000-6000 meaning that some of the cofilin binding events consist of multiple molecules (Response Figure 11C). These clusters represent a smaller fraction of

the total cofilin events analyzed <10%. Single molecules of cofilin are not thought to be able to sever on their own, and this perhaps explains why some events are long lived.

Secondly, we attempted to look for severing events using maximum intensity projections of different parts of the single molecule imaging sequence, so that we could visualize the filament backbone. We broke up a series of 600 frames into two sets of 300 frames and created maximum intensity projections (MIP) from the first half and second half of the frames, allowing us to compare filaments 'before' and 'after' to detect potential; severing events that occurred at some point in the first set of frames (Response Figure 12).

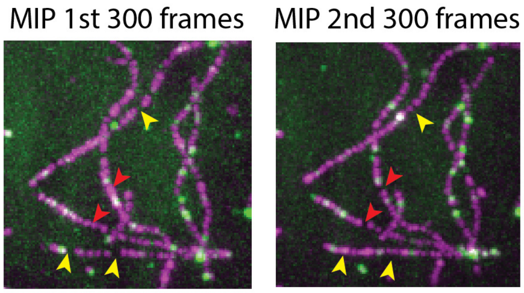

Response Figure 12: *Separation of single molecule imaging sequences into two maximum intensity projections to generate an image of the filament backbone and detect filament severing. Red arrowheads indicate contiguous filaments in the first half of the timelapse that appear as breaks in the second half of the timelapse. Yellow arrowheads indicate contiguous filaments in the second half of the timelapse that appear as breaks in the first half of the timelapse.*

Most of the filament backbones were preserved in both intensity projections indicating that the overall severing rate was low. To examine where severing events were occurring, we compared local regions on the filament backbones.

In the images above (Response Figure 12), red arrowheads indicate regions where a non-contiguous filament backbone is detected in the second sequence MIP where it had been contiguous in the first sequence MIP. While these 'breaks' might be severing events, similar 'breaks' were observed in the first sequence that appeared contiguous in the second sequence, shown by the yellow arrowheads. This indicates that 'breaks' in these images were not necessarily severing events and it was not possible to distinguish a severing event from a region of unlabeled actin filament with this approach. We postulate that severing events that are occuring could potentially relieve conformational changes induced by cofilin binding to F-actin. Therefore, the differences that we observe in our single molecule 'near' vs 'far' experiments might in fact be an underestimation of the effect that cofilin binding has on the dwell time of utrophin mutants.

Do cofilin clusters grow over time? If not, could it be that they stop growing when they reach a phalloidin anchor to the surface? Controlling the anchor density would also be very helpful, since the global severing rate depends on the density of anchoring points (see Pavlov et al. JMB 2007).

By analyzing cofilin intensity in our movies, we find that single molecule cofilin binding events can grow over time (Response Figure 11C). In the above intensity measurement, we monitor the intensity of a cofilin events over time. We can see that intensity can both increase to single molecule levels (~2000) and decrease, which corresponds to binding and unbinding of cofilin (Response Figure 11B). We can also observe increases above ~2000

over time which corresponds to the growth of a cofilin cluster (Response Figure 11C). This observation, in combination with our bulk severing assay measurement, shows that cofilin clusters can grow and sever actin filaments in our assay. We agree with the reviewer that differences in surface anchoring can affect severing and the activity of cofilin. We did not change the tethering conditions in our assay for cofilin severing experiments so that we could directly compare with results from other experiments.

The utrn binding events taking place near the cofilin clusters may be partially on the cofilin clusters. Can this be considered, or ruled out? Here as well, data on cofilin-saturated filaments would help.

The reported differences in dwell time do not distinguish being very near to, or directly within cofilin clusters due to the spatial resolution of our imaging. As higher resolution microscopy techniques continue to be developed it will be interesting to combine higher resolution imaging with the kSTORM method we present here and revisit this question. Such a higher resolution approach could be used to investigate how different actin filament conformations within a cofilin cluster compare to those at different distances from the cofilin cluster. Indeed, the propagation of conformational change in f-actin due to cofilin is an active area of research with both local and global changes are being reported[12–14].

In Fig 4D, it would be useful to show the control situation (no cofilin), as a comparison.

Thank you, we have added this experiment and reported the values.

With drebrin: are the filaments saturated? Are they exposed prior to utrn, or directly to both proteins at the same time? Do utrn mutants bind where drebrin is, or away from it?

As mentioned in our comment above, utrophin and drebrin share similar binding sites on F-actin. The drebrin construct used in our experiments, drebrin AA1-300, has an actin binding affinity reported to be ~100-200nM[9,10]. We therefore expect actin filaments in our assay to be close to saturation (~90% saturated) but still allow single molecule levels of utrophin actin binding domains to bind to the filament. Both proteins are introduced into the assay at the same time. Response Figure 13 shows an image of 200nM alexa488 labelled drebrin1-300 decorating actin filaments. This concentration produced highly decorated actin filaments.

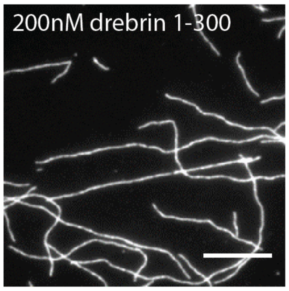

Response Figure 13: *Labelled actin binding domain of drebrin (AA 1-300) decorating actin filaments. Scale bar 5µm.*

4. Experiments with myosin

We apologize that the experiments involving acto-myosin networks were not clear in our

manuscript. We have decided not to include these experiments in the revised manuscript so that we can focus on the single molecule assay used throughout the manuscript, hopefully improving consistency and clarity. Despite this change, we have still added additional experiments and included the results here to address the Reviewer's comments (Response Figure 14).

We performed two key experiments to investigate the role of myosin activity on the binding of the different utrophin ABD mutants. Firstly, we prepared crosslinked actin networks and imaged the bulk localization of the different actin binding domains within these networks. Secondly, we measured the single molecule dwell times of the ABDs to actin filaments tethered to the surface (in the same way as for previous experiments) in the presence of the myosin fragment Heavy Meromyosin (200nM) in solution, to investigate how HMM binding might allosterically impact the dwell times of ABDs.

How does blebbistatin affect myosin binding to the filaments? In Fig 4D there seem to be a lot of actin filament bundles, are they due to blebbistatin? These bundles make the comparison with the images in Fig 4B difficult and unreliable. Perhaps the authors should have used an ATP-free situation rather than blebbistatin to turn off contraction (as in the recent preprints from the Alushin lab).

We followed previous work to generate crosslinked actin networks using the actin crosslinking protein alpha actinin, and myosin II filaments[15–17]. To generate actin networks, alpha actinin, myosin and two of the fluorescently labelled ABDs (for ratiometric localization comparison) are mixed and incubated for 5 minutes, allowing myosin mini-filaments to assemble. G-actin is then added to this mix and flowed directly into the imaging chamber. Actin rapidly polymerizes and is crosslinked in these conditions and assembles into a crosslinked network. Because actin is polymerized in these conditions, we are not aware of a way to add and remove ATP from the networks by washing. Therefore, we opted to use blebbistatin, which is commonly used as an inhibitor of myosin II ATPase. Since blebbistatin acts on myosin II ATPase, it does not interfere with actin binding, but instead blocks the myosin heads in a low affinity complex with actin[18], mimicking ATP depletion to some extent. Blebbistatin therefore presents a convenient way to change actin network contractility and has been widely used both *in vitro* and *in vivo*.

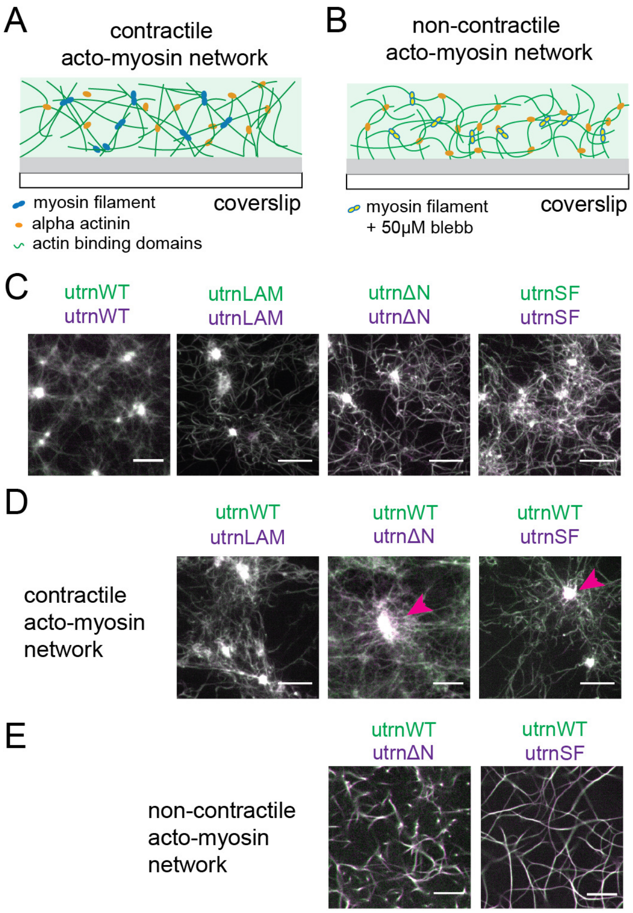

Response Figure 14: *Comparison of the localization of labelled utrophin actin binding domain mutants in actin networks. (A) Contractile actin networks generated with actin filament crosslinking by alpha actinin and contraction by myosin II. (B) Non-contractile networks generated by inhibiting myosin activity with blebbistatin. (C) controls showing the same actin binding domains in both color channels. (D) Localisation of utrnLAM, utrnΔN and utrnSF (magenta) in contractile networks with respect to utrnWT (green). (E) The localization of utrnΔN and utrnSF in non-contractile networks. Scale bars are 10μm.*

For the actin-network experiments, we have carried out the appropriate control for this measurement, including imaging the same fluorescently labelled ABD in both color channels for the different network conditions (Response Figure 14). This control can be used as a direct comparison, irrespective of the network geometry. However, as the Reviewer points out, actin networks can be very heterogeneous. Since this experiment is different from the rest of the assays used in the manuscript, we have decided not to include this data in the revised manuscript.

When comparing dwell-times, what is the control situation? Filament networks without myosin, but with alpha-actinin? Raw data of fluorescent spots should be provided, as for other experiments.

We did not perform single molecule measurements in actin networks. Rather, we imaged the bulk localization of the different actin binding domains. We apologize for the lack of clarity in our original manuscript on this point.

We performed single molecule binding measurements in the presence of the Heavy Meromyosin fragment of Myosin, which does not form myosin mini-filaments (Figure 6). For the single molecule measurements with HMM, we compared utrophin ABD binding on HMM decorated actin filaments to undectorated actin filaments in a similar fashion to our experiments with jasplakinolide, phalloidin and drebrin. The k_d of HMM is ~3 nM and therefore actin filaments should be 100% saturated with HMM in our experimental conditions[19].

5. physical confinement

There is absolutely no evidence that the observed clustering is due to the anchoring points (there is no control, no variation of anchor density). The "clustering of dwell times" is observed because of a change in the observation method, not a change in the anchoring conditions.

We thank the Reviewer for this comment and agree that clustering needs to be addressed with additional experiments. To do this, we compared clustering measured using kSTORM on filaments attached to rigid glass surfaces and fluid supported lipid bilayers (SLB), both tethered through biotin-phalloidin (Response Figure 15). Attaching actin filaments to a fluid lipid surface allows any stresses in the filament resulting from binding to relax as tethering to the surface takes place. This experiment provides a way to compare different mechanical constraints while using the same biochemistry to attach filaments to a surface in a geometry that is compatible with single molecule TIRF microscopy. We previously attempted to change anchor density in the original assay, as the Reviewer suggests, but we found that filaments would begin to fluctuate out of the TIRF field and hence were not compatible with single molecule imaging.

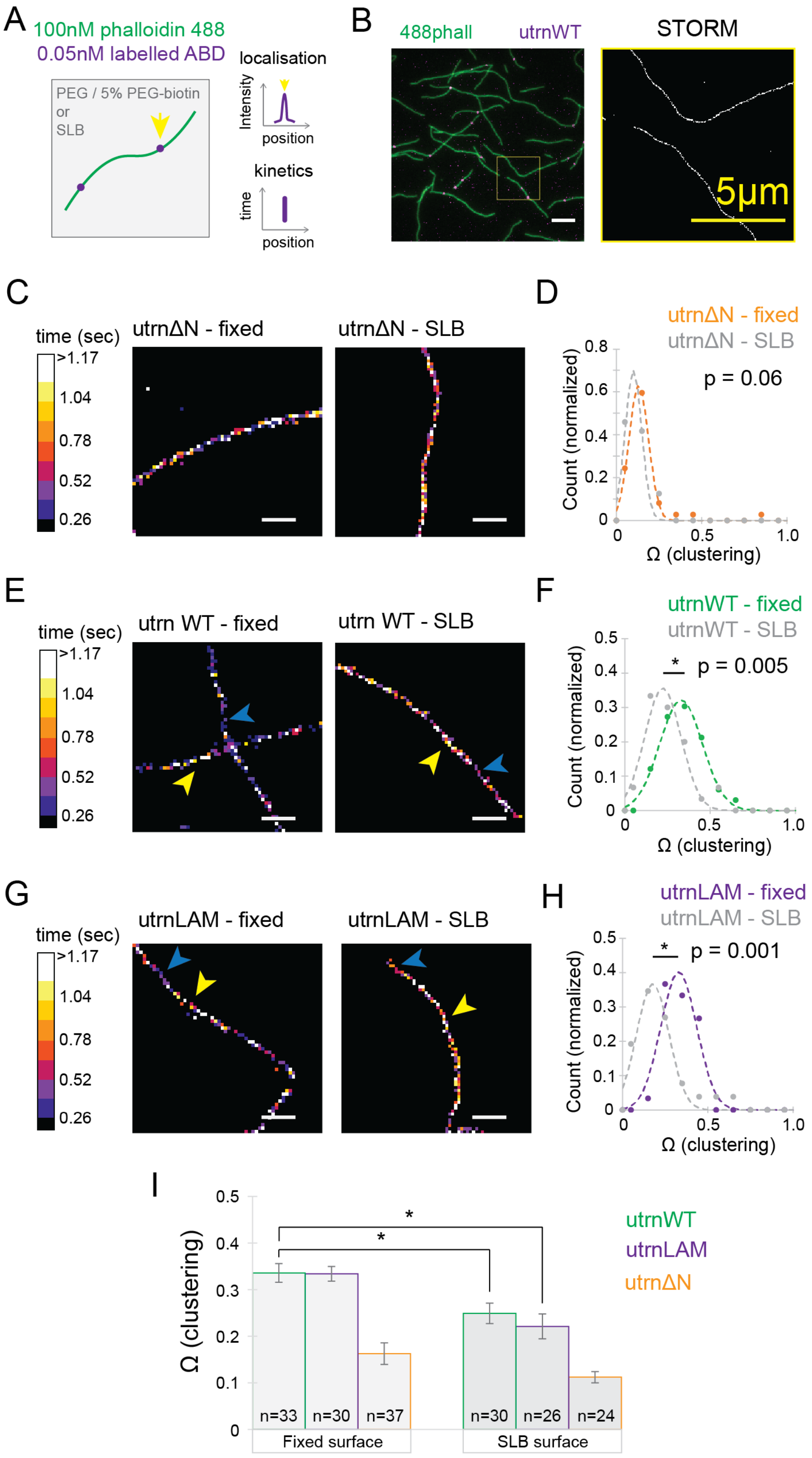

Response Figure 15: *(A) kSTORM combines subpixel localization with measurement of binding kinetics at that location. (B) kSTORM images are generated using low concentrations of 488-phalloidin (100nM) which is used for image registration and correction for sample drift. Example STORM reconstructions of single molecule measurements from utrnWT (right). Scale bar is 5μm. (C) kSTORM images for utrnΔN on glass and supported*

lipid bilayer (SLB) surfaces. Scale bar is 1μm. (D) Quantification of clustering in the different surface conditions (right). (E) kSTORM images for utrnWT on glass and supported lipid bilayer (SLB) surfaces. Scale bar is 1μm. (F) Quantification of clustering in the different surface conditions (right). (G) kSTORM images for utrnΔN on glass and supported lipid bilayer (SLB) surfaces. Scale bar is 1μm. (H) Quantification of clustering in the different surface conditions (right). (I) Summary of clustering in different conditions.

We can see from the data that clustering of dwell times is different for filaments tethered onto an SLB versus filaments immobilized onto a rigid surface (Response Figure 15, Figure7). This result points to how physical confinement can impact actin filament conformation and feeds back on the dwell time of actin binding proteins.

If anchoring to the surface had an impact on utrn binding kinetics (and it might, indeed), that would put in question all of the previous results where filaments were also anchored to the surface.

The Reviewer raises an interesting and important point. Our results do suggest that tethering filaments to surfaces could impact reported binding kinetics, but the magnitude of the impact will vary depending on the protein and the degree of confinement. It may also be the case the filaments anchored to a surface could, under some conditions, present a broad range of filament conformations such that variability in kinetics is increased but mean binding affinity is still accurate. Nonetheless, we do believe that our observation of conformation-dependent binding introduces an important new way of thinking about protein binding to actin filaments and a new perspective on how binding could be regulated by mechanical confinement.

6. The experiments are not sufficiently described, and basic information is missing.

The reader needs to get an idea of how each experiment is done, without going back and forth to the Methods section. And the Methods section does not always help. For example, the description of the "actin network assay" is very unclear. After reading it several times, I still have no idea how the experiment is done.

We apologize for the omission of important information regarding our methods. We have updated the manuscript main text to explain the experimental progression more clearly and the motivation for each experiment. We have included additional details in both the text and figures to make key information about the methods more accessible for readers. As indicated above, we have decided not to include the myosin-based actin network assay data in the revised manuscript.

All protein concentrations should be indicated in the figures or their caption (not just in the Methods).

We have added protein concentrations used directly to the figures.

Most scales bars have no numbers, and are not specified in the caption. There are no scale bars for distances in kymographs.

We have added distance information for the kymographs directly to the figures. For scale bars, we have specified the distance in the caption when not on the figure its self.

The only information about cofilin is that it was "a kind gift from Peter Bieling" (in methods). How is it labeled? What isoform is it? From what organism?

We have added additional details for cofilin to the materials and methods (Atto488-ybbr-hCofilin I). A reference detailing purification and labelling of cofilin was also added[11].

The composition of F-buffer is not given. The buffer used for experiments is never specified, except "AB buffer" for which the pH is unknown.

We have updated the materials and methods to give full details of the buffers used.

I am sorry to have to say this, but so much information is missing that I often wondered how many times the authors had read their manuscript before submitting it.

We apologize for the missing information in the manuscript and have added additional details throughout to improve clarity.

7. Minor points

In Fig 1 the purple residues are not easy to see.

We have replaced the images in figure 1 to improve visualization.

Missing panel in Fig S3, and wrong letters (E, F) in its caption.

We have corrected this and added photobleaching data to Fig S4.

At least twice, the text seems to imply that severing scales with cofilin concentrations. It does not. It scales with the number of cofilin cluster boundaries, as indicated in the introduction.

We have updated the text for consistency.

Lots of letter are not capitalized, while it seems that, by convention, they usually are: f-actin (F-actin), g-actin (G-actin), n-terminal (N-terminal).

We have updated the text to incorporate these changes and be consistent with convention.

Fig 3D and 4H, y-axis: "fraction (tau1 to tau2)" seems incorrect. This is not a ratio of times, is it? Rather, it is "a1", referred to earlier as "relative amplitude of the two timescales", and which actually seems to be the fraction of unbinding events that belong to the fast population. Is that correct?

The Reviewer is correct in that by fraction, we mean the fraction of the total events belonging to each timescale. We realize that our notation is unclear and have updated the axis title to relative amplitude to be consistent with the text.

Several typos, missing or extra words. (e.g., bottom of page 10, "we measured generated…") and inaccuracies (e.g., top of page 13, a "dwell time" is a number, it cannot be "longer lived")

We thank the Reviewer for pointing out these mistakes. We have corrected these and checked the manuscript for any additional errors.

Sample size should be indicated in figures (in particular, when plotting a CDF).

We have added the sample size to the figures and descriptions in the text. For CDF data we have added the total number of molecules directly to the figure and indicated the number of replicates in the statistics section.

Reviewer #2 (Remarks to the Author):

In the manuscript "Biased localization of actin binding proteins by actin filament conformation" Harris et al. present abundant binding data of different actin-interacting proteins and show that these are influenced by the actin structure. In general, I find the results very interesting and useful. However, I have a technical comment on the kSTORM measurements, as well as some minor comments.

Major comment, kSTORM analysis, Fig. 6: The idea to look at spatial variability of kinetic constants by kSTORM is certainly very interesting. However, I am not convinced that the results in Fig. C-E show more than noise.

We thank the Reviewer for the comments and suggestions, as well as the interest in the method that we present to investigate the spatial variability of kinetic measurements. The Reviewer raises an important point regarding the contribution of stochastic variability to the measurement of dwell time. We have added additional analysis and a stochastic simulation to address this question and verify our observations. We also added new controls to test how tethering to different types of substrates (fluid versus rigid) impact spatial variability in dwell times and clustering.

a) The on-times that are plotted here follow an exponential distribution. Thus, in each pixel many binding events need to be detected to estimate the mean with a high accuracy. This information needs to be presented (e.g. a second image showing the counts per pixel). Also, consider taking longer measurements.

This is an important point raised by the Reviewer. We have added the converged dwell time images, average images and counts per pixel images to the Supplementary Information of the manuscript (Supplementary Figure 8, Response Figure 16).

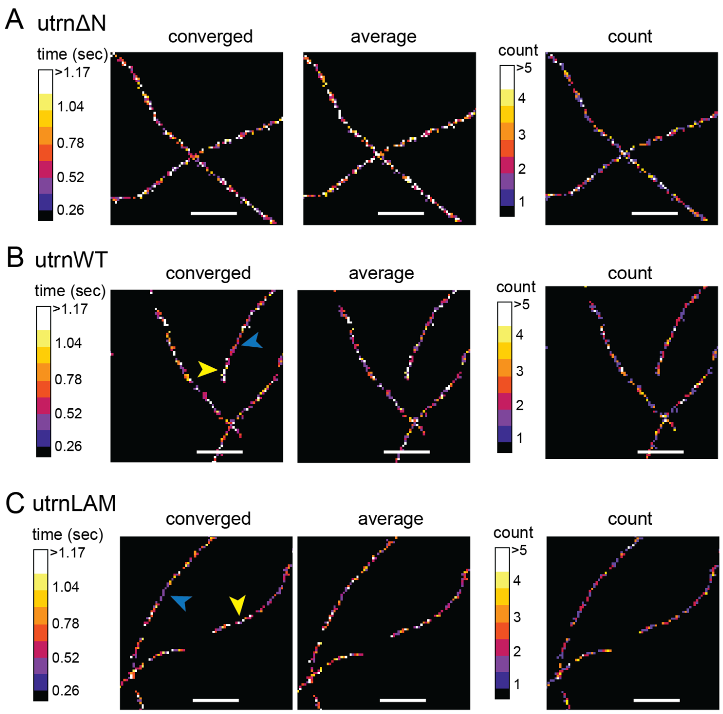

Response Figure 16: *Different projections shown for kSTORM imaging. The converged images shown in the left, the average dwell time images in the middle and the counts per pixel shown on the right. Scale bars are 1µm. Images shown for (A) utrnΔN, (B) utrnWT and (C) utrnLAM.*

The images that we present in the main text are converged images, where the dwell time at each pixel is the ultimate value that was measured at that location through the image stack. Taking longer measurements is desirable but presents practical limitations. To measure single molecule locations precisely, low concentrations of labelled protein are added, and many frames (~12,000) with a long acquisition, is required to build up the STORM image. As a result, it is not practically possible with our current setup to generate a full CDF at each pixel (with for example 100 events), and instead the pixel value represents a sub-sample of that CDF. On average we have ~3 counts at each pixel that we can use to make the average dwell time measurements.

Because of this limitation, we developed a simulation to investigate how stochastic variation from a double exponential distribution of dwell times impacts clustering and other metrics we use for quantification. Our simulation was based on a monte carlo method, drawing dwell times for binding events from a double exponential distribution. The characteristic dwell times $\tau 1$ and $\tau 2$ as well as the relative amplitudes of these timescales were inputs for the simulation and based on our experimental measurements. The iteration timestep of the simulation was then set to be 100ms, similar to the frame rate used in our experimental imaging acquisition, 130ms. Binding events were assigned randomly along a line to represent the filament backbone. With each iteration of the simulation binding events were removed (unbinding event) after being bound for their chosen dwell time. The number of bound molecules was kept constant (decoration 5%) by binding a new molecule to a free binding site on the backbone after an unbinding event. Using this approach, we were able to generate kymographs of binding events along the backbone that resembled those from our

experimental measurements. To verify that the simulation resembled the experimental measurements, we calculated the cumulative distribution function of binding dwell times and compared it to our experimental measurements. We see good agreement between the experimental and simulated data (Response Figure 17). We then tested the metrics used for clustering for both randomly assigned dwell times and dwell times that are clustered into a given region. Interestingly, the metric we use to measure spatial clustering (Ω) of the second timescale returned a value of Ω = 0.13±0.04, which was similar to our experimental measurement for utrnΔN. When we introduced clustering into our simulation by dwell times belonging to the second timescale τ_2 into distinct regions along the backbone, we measure a spatial clustering value Ω= 0.31±0.05, which was similar to our experimental measurement for utrnWT and utrnLAM.

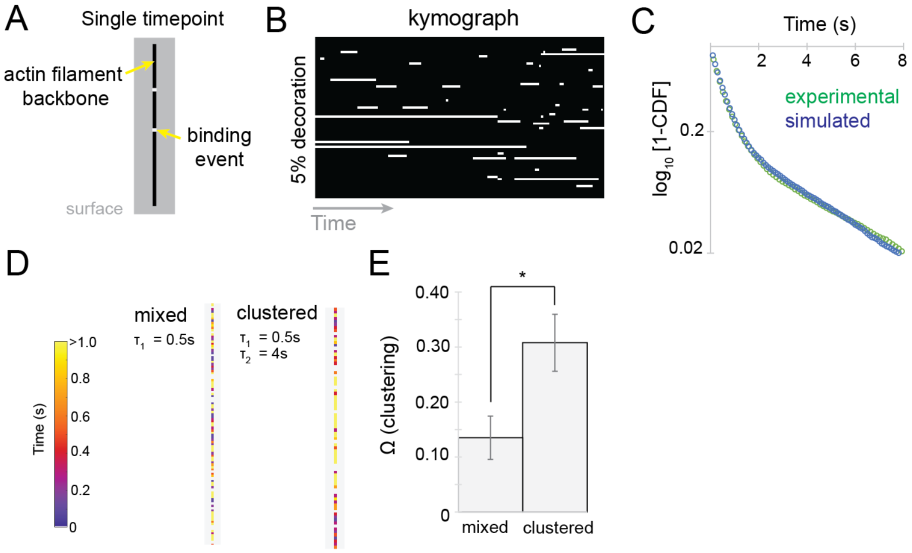

Response Figure 17: *Stochastic simulation of kSTORM imaging. (A) Monte Carlo based assignment of points along a line to represent the actin filament. Events dwell on the actin filament for a given time drawn from a double exponential distribution which was calibrated from the experimental data. (B) Kymograph of dwell times. (C) Comparison of experimental and simulated data using the characteristic timescale and relative amplitudes for utrnWT. (D) Simulated randomly mixed and clustered dwell times along the backbone. (E) Mixing, Ω, measured for each of the simulated conditions.*

b) From the counts per pixel, please estimate the statistical error in t and compare it with the variation along the fibril to show that these are not statistical in nature but denote real variation.

We measured the standard deviation of dwell times both in time and along the fibril and then compared these metrics. As the Reviewer suggests, if the variation in both space and time are the same, then both reflect stochastic variation. We measured the standard deviation in time at each pixel to be σ_{time} = 1.4 sec for utrnWT. Along the fibril the standard deviation in time was $\sigma_{position}$ = 2.8 sec. The higher standard deviation along the fibril indicates that there is a spatial variation in dwell time along the filaments that does not simply arise from stochastic variation that we might expect from the exponential distribution of dwell times. We made similar observations for utrnLAM σ_{time} = 0.9 sec, $\sigma_{position}$ = 2.2 sec and utrnΔN σ_{time} =

1.3 sec, $\sigma_{position}$ = 2.1 sec. The standard deviation in dwell time along the fibril was the lowest for utrnΔN, potentially indicating that this construct had a lower level of clustering than the other constructs. As a comparison, we measured the standard deviation of dwell times in our simulated images of kSTORM. When we simulated clustering in these images, the standard deviation in dwell time was higher than for images where there was only stochastic variation ($\sigma_{clustering}$ = 3.4 sec, $\sigma_{stochastic}$ = 2.6 sec) consistent with the trend shown in our experimental measurements.

c) A control I would like to see is to divide the data set into two sets and to plot the t value in the first vs the t value in the second half for each pixel in a scatter plot. Only if there is a strong correlation, we can interpret the actual t values in the pixels as being more than noise.

This is an interesting suggestion from the Reviewer. As we described above, the time that it takes to acquire a kSTORM image limits number of measurements at each pixel and our ability to reliably split the data into two sets and compare the measurements between each set. To address the Reviewer's question, we developed a simple stochastic simulation to test the role of stochastic variation on the spatial variation of dwell times which is described above and in Response Figure 17. We have also added an additional experiment which is to compare the spatial clustering of dwell times on filaments attached to a supported lipid bilayer (SLB). The SLB allows some of the stresses to relax as filaments attach to the surface. We see a reduction in the degree of clustering in these conditions. If it were the case that clustering was simply a result of stochastic variation, then no differences would be observed (Response Figure 18). In future work, we hope to continue to develop the kSTORM experimental technique to both increase the spatial resolution that can be achieved and provide longer measurements (for example, improving subpixel drift correction that occurs over multiple hours of imaging).

d) Please explain the Omega analysis better and motivate why it is applicable here (there are many algorithms that quantify clustering in images, why take an approach from genomics? What does it really measure?).

We have updated the materials and methods section of the manuscript with additional details on the choice of clustering metric we have used. To compare clustering in the different kSTORM images, we used the mixing parameter Ω, which has been previously used to analyze local variation in sequence data[20]. In our analysis, we used Ω to compare the clustering of the two different characteristic timescales τ_1 and τ_2. We used a threshold of 1 second which separates the two timescales in the CDF, with $f_{\tau 1}$ = the fraction of pixels having a dwell time less the threshold and $f_{\tau 2}$ = the fraction of pixels above the threshold. ∂ is then the average deviation with σ measured both for the whole fibril and at local regions 6 pixels long over the length of the filament.

$$\sigma = (f_{\tau 2} - f_{\tau 1})^2$$

$$\partial = \frac{\left(\sigma_{\text{local}} - \sigma_{global}\right)^2}{N}$$

$$\Omega = \frac{\partial}{\partial_{max}}$$

This metric therefore reports the mixing of pixels belonging to each timescale along the filament. We also evaluated this metric with our stochastic simulation (Response Figure 17)

to verify its use in quantifying clustering along the filament length. We chose to use this metric as it is applicable for measuring clustering of two species along a 1D space such as the filament backbone. We have updated the materials and methods section of the manuscript to include these additional details.

e) Is the interpretation based only on the shown figures? Or on how many figures? More statistical analysis is needed to demonstrate that indeed C-E show different degrees of clustering, including the mean +/- SEM of the clustering metric for the different conditions and number of experiments.

We have added statistical analysis including the number of regions analyzed and the distribution of clustering values for the different proteins and experimental conditions. We have reported both the distribution in the figure and the mean +/- SEM in an additional figure panel with the number of individual measurements (Figure 7, Response Figure 18).

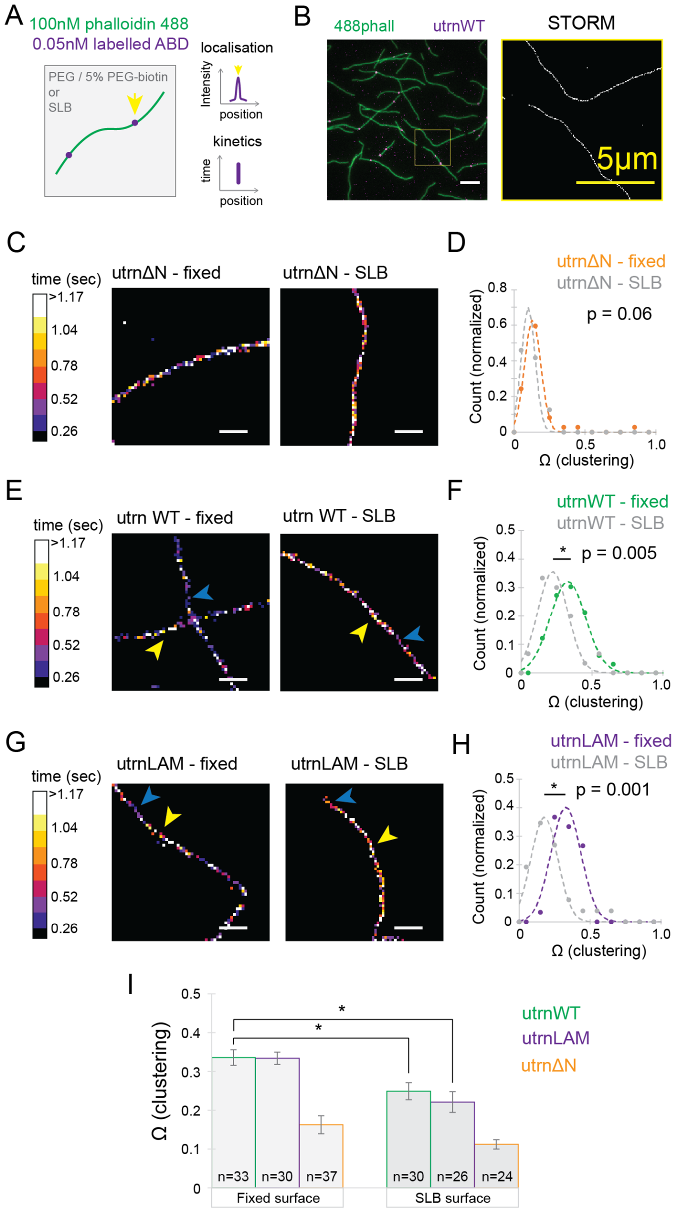

Response Figure 18: *(A) kSTORM combines subpixel localization with measurement of binding kinetics at that location. (B) kSTORM images are generated using low concentrations of 488-phalloidin (100nM) which is used for image registration and correction for sample drift. Example STORM reconstructions of single molecule measurements from utrnWT (right). Scale bar is 5μm. (C) kSTORM images for utrnΔN on glass and supported lipid bilayer (SLB) surfaces. Scale bar is 1μm. (D) Quantification of clustering in the different surface conditions (right). (E) kSTORM images for utrnWT on glass and supported lipid bilayer (SLB) surfaces. Scale bar is 1μm. (F) Quantification of clustering in the different surface conditions (right). (G) kSTORM images for utrnΔN on glass and supported lipid bilayer (SLB) surfaces. Scale bar is 1μm. (H) Quantification of clustering in the different surface conditions (right). (I) Summary of clustering in different conditions.*

Minor comments
1. The quantitative measurements of the two kinetic contestants for different mutants and proteins could be very useful for other work, including simulations. To exclude that photo bleaching has an effect on the values (especially the long time constant) I would like to see additional controls. Either taking the same measurements at different intensities to test from which intensity value a change in the kinetic parameters occurs, or an independent measurement of photo bleaching times to demonstrate that they are much longer than the slow time scales.

We thank the Reviewer for this suggestion. We have now incorporated the contribution of photobleaching into our analysis (as also requested by Reviewer 1). We characterized bleaching in two different ways.

(1) Firstly, we measured the bleaching rate of Streptavidin conjugated to Alexa555 attached to our biotinylated glass coverslips (Response Figure 19A). Alexa555 is the same dye we use to measure actin binding domain kinetics. The high affinity of the Biotin-Streptavidin interaction (~10^{-14} M) means that loss in fluorescence molecules during imaging is dominated by bleaching and allows us to characterize the overall bleaching rate. We used the same imaging conditions as for our single molecule experiments including the laser power (~7mW) and exposure time (130ms per frame, 600 frames). The bleaching time constant we measured was 5.6 ± 0.1 seconds. We note that this is approximately double that of the second timescale (longer) of the utrophin mutants that we measured (utrnWT = 3.2 ± 0.3 sec, utrnLAM = 3.0 ± 0.1 sec and utrnN = 2.3 sec ± 0.1 sec).

(2) Secondly, we repeated the single molecule utrnWT binding measurement with a range of different laser powers to examine potential changes in the second timescale, as suggested by Reviewer 2 (Response Figure 20B). We observed little change in the second timescale between 30% and 40% power settings (~7 mW and ~10 mW measured out of the objective), though the measured second timescale did begin to change significantly (τ_2 = 2.17 ± 0.4 sec, a 32% change) at double the laser intensity (60% power ~21mW).

Based on these measurements we can conclude that the double timescale that we observe is not due to photobleaching, since (1) the photobleaching time is longer than our measured timescale and (2) we use laser powers <10 mW. However, to account for the photobleaching rate in our measurements, we added a correction to the measured timescales for each actin binding domain based on the approach of Hayakawa et al[1]. The bleaching rate can be subtracted from the measured off-rate to provide a corrected off-rate (inverse of the dwell time), where $\frac{1}{\tau corr} = \frac{1}{\tau measured} - \frac{1}{\tau bleach}$. In the manuscript (Figures 2-6), we now report values in bar charts for both τ_1 and τ_2 as bleaching corrected dwell times. For cumulative distribution function plots (Figure 2 and Supplementary Figures 4,6,7), we left these as the raw collected data.

The bleaching measurements have been added to the manuscript in the supplementary information (Supplementary Figure 4). We have added additional details both to the materials and methods and figure captions to clarify this point.

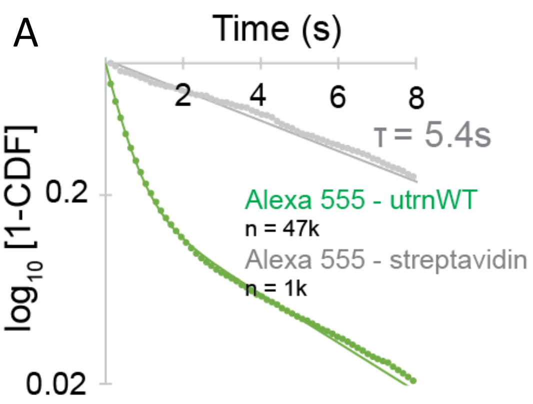

Response Figure 19: *(A) Measurement of the photobleaching rate of alexa555-streptavidin. (B) Measurement of the CDF of utrnWT imaged with different laser powers. 30%, 7mW shown in green, 40% 10mW shown in orange, 60% 21mW shown in blue.*

2. All plots showing the 1-CDF. The y-axis is a bit confusing. I assume it is in logarithmic units? Then please point this out and add a few more values to make it clear. Also, for comparison, it would be great to scale all 1-CDF plots the same way with the y-axis ranging from 0.02 to 1.

We thank the Reviewer for pointing this out and have updated the axis titles to Log_{10} [1-CDF] to reflect that this is a logarithmic axis. All CDFs are now plotted from 0.02 to 1 for consistency.

3. Fig. 3A. The authors write that phalloidin has no effect on utm wt or LAM, but that Jasp does. Looking at the graphs I don't agree with the interpretation. If a result is not significant, it does not mean there is no effect. The change in t1 is about 1/3 as big for phalloidin as for Jasp, so there seems to be an effect, however a weaker one for phalloidin compared to Jasp.

We thank the Reviewer for this point. We have updated our description in the manuscript to include that there might be a small difference in the presence of phalloidin, although this was not statistically significant.

4. Fig. 4 D, Fig. S4. I am not entirely convinced that the results are not just due to statistical variability. Reporting the confidence intervals of fits is different to looking at actual variations of results (as is done for all the rest of the manuscript). I would prefer fitting individual experiments individually. If this is not robust, consider fixing t2 and its amplitude (same for far vs near) to show that there is indeed a significant change in t1. Or take more data.

We thank the Reviewer for this comment. In order to address this, we have taken additional data as well as updated the analysis so that statistical comparisons can be made between replicates. The error bars now represent the standard error of the mean based on experimental replicates, and we have updated the materials and methods and statistics section of the manuscript. To generate these fits, we fixed τ_2 and its amplitude as measured for the 'far from cofilin' data and fit τ_1 as a free parameter for the near cofilin CDF, as the

Reviewer suggested (Response Figure 20 and 21). There was a change in τ_1 for utrnWT and utrnLAM that was statistically significant, in addition to a smaller change in τ_1 for utrnΔN that was not statistically significant which is consistent with our initial observation. We conclude that conformational changes induced by cofilin binding impact the dwell times of the utrophin mutants in different ways.

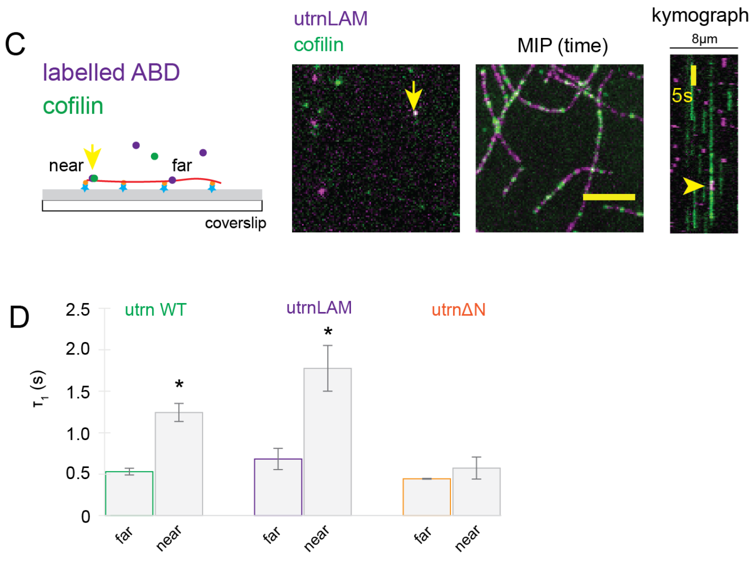

Response Figure 20: *Experiments measuring utrophin ABD binding events near and far from cofilin binding events. (C) The experimental paradigm. (D) the τ_1 timescales for the different utrophin mutants near and far from cofilin binding events. The timescales were measured by fitting the CDF's shown in Response Figure 22.*

The CDFs have been updated and added to the supplementary information in the manuscript file, shown below.

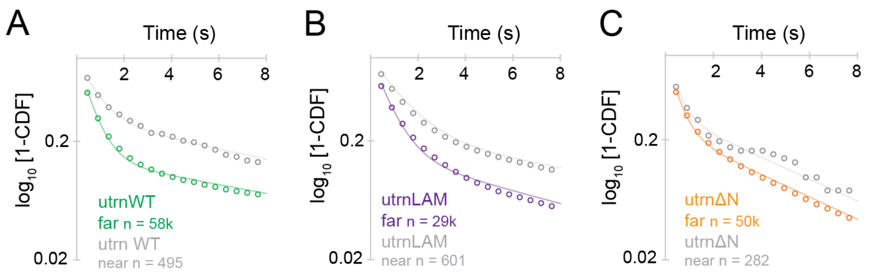

Response Figure 21: *CDF's for cofilin 'near' vs 'far' experiments for three different actin binding domain mutants.*

5. Figure S3: F is missing.

We have corrected Fig S3 and its reference.

References

1. Hayakawa, K., Sakakibara, S., Sokabe, M. & Tatsumi, H. Single-molecule imaging and kinetic analysis of cooperative cofilin–actin filament interactions. *Proc. Natl. Acad. Sci.* **111**, 9810–9815 (2014).
2. Harris, A. R. *et al.* Steric regulation of tandem calponin homology domain actin-binding affinity. *Mol. Biol. Cell* mbc. E19-06-0317 (2019).
3. Galkin, V. E., Orlova, A., Salmazo, A., Djinovic-Carugo, K. & Egelman, E. H. Opening of tandem calponin homology domains regulates their affinity for F-actin. *Nat. Struct. Mol. Biol.* **17**, 614–616 (2010).
4. Iwamoto, D. V. *et al.* Structural basis of the filamin A actin-binding domain interaction with F-actin. *Nat. Struct. Mol. Biol.* 1 (2018).
5. Kumari, A., Kesarwani, S., Javoor, M. G., Vinothkumar, K. R. & Sirajuddin, M. Structural insights into actin filament recognition by commonly used cellular actin markers. *EMBO J.* **39**, e104006 (2020).
6. Pospich, S., Merino, F. & Raunser, S. Structural effects and functional implications of phalloidin and jasplakinolide binding to actin filaments. *Structure* (2020).
7. Wulf, E., Deboben, A., Bautz, F. A., Faulstich, H. & Wieland, T. Fluorescent phallotoxin, a tool for the visualization of cellular actin. *Proc. Natl. Acad. Sci.* **76**, 4498–4502 (1979).
8. Bubb, M. R., Senderowicz, A. M., Sausville, E. A., Duncan, K. L. & Korn, E. D. Jasplakinolide, a cytotoxic natural product, induces actin polymerization and competitively inhibits the binding of phalloidin to F-actin. *J. Biol. Chem.* **269**, 14869–14871 (1994).
9. Grintsevich, E. E. *et al.* Mapping of drebrin binding site on F-actin. *J. Mol. Biol.* **398**, 542–554 (2010).
10. Grintsevich, E. E. & Reisler, E. Drebrin inhibits cofilin-induced severing of F-actin. *Cytoskeleton* **71**, 472–483 (2014).

11. Hansen, S. D. *et al.* αE-catenin actin-binding domain alters actin filament conformation and regulates binding of nucleation and disassembly factors. *Mol. Biol. Cell* **24**, 3710–3720 (2013).

12. Galkin, V. E. *et al.* Remodeling of actin filaments by ADF/cofilin proteins. *Proc. Natl. Acad. Sci.* **108**, 20568–20572 (2011).

13. Huehn, A. *et al.* The actin filament twist changes abruptly at boundaries between bare and cofilin-decorated segments. *J. Biol. Chem.* **293**, 5377–5383 (2018).

14. Ngo, K. X., Kodera, N., Katayama, E., Ando, T. & Uyeda, T. Q. Cofilin-induced unidirectional cooperative conformational changes in actin filaments revealed by high-speed atomic force microscopy. *Elife* **4**, e04806 (2015).

15. Lieleg, O., Claessens, M. M. & Bausch, A. R. Structure and dynamics of cross-linked actin networks. *Soft Matter* **6**, 218–225 (2010).

16. Falzone, T. T., Lenz, M., Kovar, D. R. & Gardel, M. L. Assembly kinetics determine the architecture of α-actinin crosslinked F-actin networks. *Nat. Commun.* **3**, 861 (2012).

17. Gardel, M. L. *et al.* Elastic behavior of cross-linked and bundled actin networks. *Science* **304**, 1301–1305 (2004).

18. Kovács, M., Tóth, J., Hetényi, C., Málnási-Csizmadia, A. & Sellers, J. R. Mechanism of blebbistatin inhibition of myosin II. *J. Biol. Chem.* **279**, 35557–35563 (2004).

19. Greene, L. E. & Eisenberg, E. The binding of heavy meromyosin to F-actin. *J. Biol. Chem.* **255**, 549–554 (1980).

20. Martin, E. W. *et al.* Valence and patterning of aromatic residues determine the phase behavior of prion-like domains. *Science* **367**, 694–699 (2020).

REVIEWER COMMENTS

Reviewer #1 (Remarks to the Author):

The authors have extensively revised the manuscript, which is now more convincing. The part on phalloidin and jasplakinolide, in particular, is now much improved. I still have a some concerns, which I think the authors can easily address (with no additional experiments).

1. The dwell times have changed because they are now corrected for photobleaching. I get that. But some other numbers have changed, with no mention of the change by the authors, and I find no explanation:

- The on-rates (Fig 2C) differ by approximately a factor 2 compared to the initial version. Why?

- The dwell times near and far cofilin spots (figure 4D) have changed, and I see no explanation. The change doesn't match the expected photobleaching correction, as far as I can tell.

These undisclosed changes are very discomforting. I had the impression that the technique was very accurate and robust, relying on a large number of events. Now I wonder how much I can trust these numbers.

2. Some statements or words are misleading and should be changed:

- About the longer dwell times near cofilin spots, the authors write "These results show that the conformational changes in F-actin induced by cofilin binding feed-back on the binding dwell times of other regulatory proteins" (page 16). This is an overstatement. The results suggest that this could be the case, but other interpretations are possible. For example, cofilin and utrn could both bind preferably to the same regions on the anchored filaments (the subsequent experiments on physical confinement indicate that such a mechanism is possible).

- Now that the experiments with actomyosin contraction have been removed, it is misleading to talk about "myosin activity". Readers will get the impression that mechanical tension is being applied. Rather, it is "myosin binding" that is studied.

3. Some key information should be highlighted:

- The fact that anchoring filaments to the coverslip alters the dwell times is an important aspect. It does not mean that the experiments made with this attachment strategy are meaningless, of course, but the reader should be made aware that the numbers reported in this paper may be biased by the mode of anchoring. This should be said clearly.

- Regarding the results of Fig2D-I, where mutations Q33A and T36A appear to counter the effect of mutation K121A, the explanations provided in the rebuttal (page 5 of the rebuttal) are interesting and insightful. Some of it should be made available to readers, in the main text.

4. Page 15 and Fig 4AB: cofilin-induced severing is a very indirect and uncertain way to estimate the amount of cofilin binding. Since these experiments are done with fluorescently labeled cofilin, I don't understand why the authors do not directly quantify fluorescence to estimate binding. I believe several readers will be puzzled, and this should be explained when introducing the experiment (or, even better, an analysis based on cofilin fluorescence could be presented).

5. I suggested a control (no cofilin) for Fig 4D. The authors replied "Thank you, we have added this experiment and reported the values" (on page 15 of the rebuttal) but I don't see anything. Perhaps telling the reader that the values "far" are comparable to the values of tau1 reported in figure 2 would

be enough.

6. Figure 4B: how are error bars determined? Has this experiment been repeated?
Fig 2A has scale bars with no numbers (not in figure legend either).

Reviewer #2 (Remarks to the Author):

The authors addressed my minor concerns and the manuscript is substantially improved and clear. However, the authors did not show that the kSTORM analysis provides robust data. Indeed, taking the additional information into account, I think it is even more likely that the authors report mainly noise. The authors could remove the kSTORM part entirely, the manuscript would still be an excellent contribution to the field, or they would need to characterize kSTORM better and show that with the current statistical limitations they can still draw conclusions.
1. The main problem I have is that the authors claim to estimate binding times from only on average three counts. For a single exponential decay, the standard deviation of the measurement is equal to its mean, for a double-exponential decay, tens to hundreds of measurements are required to estimate the two time constants.
2. From the previous experiments, the authors have estimates on tau1 and tau2, from those they could estimate the standard deviation of the distribution, and taking each count as a measurement they could plot the SEM of the kSTORM rendering as std/sqrt(counts). The counts need to be mentioned in the figure legend, I strongly recommend adding this SEM image to Figure 7.
3. I do not understand the difference between converged and average images. Please specify in S8 precisely what these images mean. I would rather add the SEM figure as suggested above and maybe show repeated images of the same structures.
4. If there is any information in the kSTORM images, then a repeated measurement should look somewhat similar. I suggested previously plotting average tau values pixelwise from one measurement vs a second measurement (or one half vs the second half). Even if the data are noisy (as expected for the small counts and if only half the data is used each time), there must be a correlation. The difference between the two measurements is an estimate for the statistical error. I would like to see this plot, even if the noise is large. There is no fundamental limitation in the length of kSTORM experiments, fluorophores are not depleted, thus taking a second measurements at the same position should be entirely feasible.
5. Although the sigma_time vs sigma_position analysis is interesting, it provides only some indication. For example, any variation of excitation intensity across the FoV can increase the variance in space. Also a statistically analysis with sometimes only 1 or 2 data points (as is common for on average 3 counts) needs to be performed properly beyond calculating the standard deviation, and much more information needs to be provided that the this comparison is meaningful.
6. As long as the values of the image have no concrete meaning, a cluster analysis seems pointless. The analysis procedure seems somewhat arbitrary. Also, the Omega analysis is not a common analysis, and it is not clear what secondary parameters (e.g. the average time constant, number of localizations,…) the Omega value could depend on. Thus, I am not convinced that the difference in Omega indeed indicates differences (or even existence) of spatial variations of the tau.
7. A minor point: Usually “STORM” is interpreted as single-molecule localization using organic dyes that enter a dark state, usually due to presence of a blinking buffer. The authors use “PAINT” and could consider adapting the acronym and citing previous literature that uses such transient binding to image structures, including actin.

Response to Reviewers

“Biased localization of actin binding proteins by actin filament conformation” Harris, et. al.

We thank the Reviewers and Editor for their careful consideration of our revised manuscript and for the additional suggestions to improve it.

In this document, we discuss each point raised by the Reviewers and describe the additional actions we have taken to address them. In particular, we have made the text modifications and carried out the additional analysis suggested by Reviewer 1, and we have removed the kSTORM experiments pending further validation experiments, as suggested by Reviewer 2 and the Editor.

Below please find our Point-by-Point Response to the Reviewers comments:

Reviewer #1 (Remarks to the Author):

The authors have extensively revised the manuscript, which is now more convincing. The part on phalloidin and jasplakinolide, in particular, is now much improved. I still have a some concerns, which I think the authors can easily address (with no additional experiments).

1. The dwell times have changed because they are now corrected for photobleaching. I get that. But some other numbers have changed, with no mention of the change by the authors, and I find no explanation:

- The on-rates (Fig 2C) differ by approximately a factor 2 compared to the initial version. Why?

The reported on-rate is now calculated from a linear regression to a concentration series that was requested by Reviewer 1, rather than a single concentration as we had used in the original manuscript. We agree with the Reviewer that fitting a range of concentrations provides a more accurate value than a single concentration, and we presented the data from our new measurements over a range of concentrations in our previous Response to Reviewers, in Response Figure 3. The difference in on-rates reflects this improved accuracy compared to on-rate estimates from a single concentration.

- The dwell times near and far cofilin spots (figure 4D) have changed, and I see no explanation. The change doesn’t match the expected photobleaching correction, as far as I can tell.

We apologize for not explaining this change more clearly. In the original review, Reviewer 2 had requested that we change our fitting procedure for the near-far cofilin spots, which now involves fixing tau2 in order to make statistical comparisons between tau1 for the near measurements. The modified method for fitting the data and the resulting analysis is detailed in the previous Response to Reviewers in Response Figures 20 and 21. This difference in fitting procedure, which is described in the Methods, is the source of the difference in dwell times.

These undisclosed changes are very discomforting. I had the impression that the technique was very accurate and robust, relying on a large number of events. Now I wonder how much I can trust these numbers.

We apologize for the confusion about the changes. We hope the explanations provided above make clear how the changes resulted from the additional data and/or analyses that were requested by Reviewers 1 and 2.

2. Some statements or words are misleading and should be changed:

- About the longer dwell times near cofilin spots, the authors write "These results show that the conformational changes in F-actin induced by cofilin binding feed-back on the binding dwell times of other regulatory proteins" (page 16). This is an overstatement. The results suggest that this could be the case, but other interpretations are possible. For example, cofilin and utrn could both bind preferably to the same regions on the anchored filaments (the subsequent experiments on physical confinement indicate that such a mechanism is possible).

We thank the Reviewer for helping us to be precise in our description of the data. We have changed the phrasing to read: "These results show that conformational changes in F-actin near a cofilin binding event may feed-back on the binding dwell times of nearby regulatory proteins."

- Now that the experiments with actomyosin contraction have been removed, it is misleading to talk about "myosin activity". Readers will get the impression that mechanical tension is being applied. Rather, it is "myosin binding" that is studied.

Good point. We replaced "myosin activity" with "myosin binding" throughout the manuscript.

3. Some key information should be highlighted:

- The fact that anchoring filaments to the coverslip alters the dwell times is an important aspect. It does not mean that the experiments made with this attachment strategy are meaningless, of course, but the reader should be made aware that the numbers reported in this paper may be biased by the mode of anchoring. This should be said clearly.

We agree that this point should be made clear. We have added the following statement to the discussion: "For example, the role of different actin isoforms was not assessed here, and the reported binding dwell times include the conformational effects of different actin filament tethering strategies."

- Regarding the results of Fig2D-I, where mutations Q33A and T36A appear to counter the effect of mutation K121A, the explanations provided in the rebuttal (page 5 of the rebuttal) are interesting and insightful. Some of it should be made available to readers, in the main text.

We agree that the compensatory interaction of some mutations is interesting. To clarify our thinking about this point, we have added further details to the beginning of the results section entitled "Two mechanisms regulate CH1-CH2 binding to F-actin."

4. Page 15 and Fig 4AB: cofilin-induced severing is a very indirect and uncertain way to estimate the amount of cofilin binding. Since these experiments are done with fluorescently labeled cofilin, I don't understand why the authors do not directly quantify fluorescence to estimate binding. I believe several readers will be puzzled, and this should be explained

when introducing the experiment (or, even better, an analysis based on cofilin fluorescence could be presented).

In the revised manuscript, we have updated the text where the experiment to clarify that we have used different concentrations of cofilin to obtain different levels of cofilin-induced severing. We have also analyzed the cofilin fluorescence in the images we presented in Fig. 4A and included this analysis in Response Figure 1.

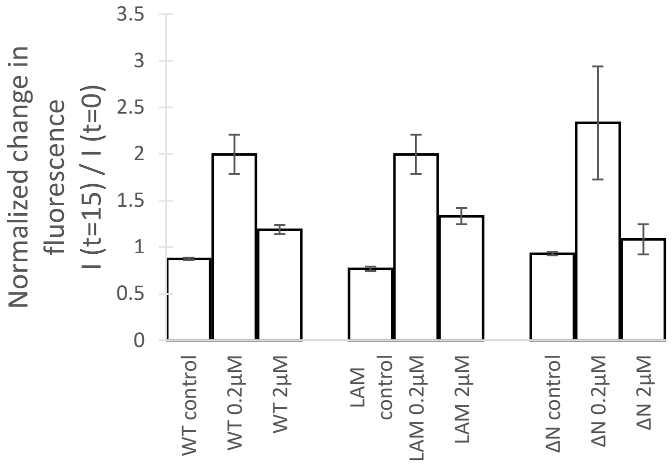

Response Fig 1: *Change in fluorescence intensity in the cofilin channel after 15 mins. Reduced cofilin intensity is observed with increasing concentration of the actin binding domain. Controls are in the absence of cofilin. Error bars are the standard deviation in intensity taken for 12 regions across 2 different sample chambers.*

5. I suggested a control (no cofilin) for Fig 4D. The authors replied "Thank you, we have added this experiment and reported the values" (on page 15 of the rebuttal) but I don't see anything. Perhaps telling the reader that the values "far" are comparable to the values of tau1 reported in figure 2 would be enough.

We apologize that in our initial response to reviewers we added no-cofilin controls to Fig 4A and were mistakenly referring to these experiments with this reply. To address the reviewers comment, we add the following statement: "In addition, the $\tau1$ values that were 'far' from cofilin in these experiments were comparable to those measured in the absence of cofilin (Fig 2A)."

6. Figure 4B: how are error bars determined? Has this experiment been repeated? Fig 2A has scale bars with no numbers (not in figure legend either).

The error bars are the standard deviation of the severing rate of >12 imaging regions collected from two different sample chambers. We have added this information to the statistics section.

We add the scale bar value to the Fig 2 caption.

Reviewer #2 (Remarks to the Author):

The authors addressed my minor concerns and the manuscript is substantially improved and clear. However, the authors did not show that the kSTORM analysis provides robust data. Indeed, taking the additional information into account, I think it is even more likely that the authors report mainly noise. The authors could remove the kSTORM part entirely, the manuscript would still be an excellent contribution to the field, or they would need to characterize kSTORM better and show that with the current statistical limitations they can still draw conclusions.

We thank Reviewer #2 for their comments and have carefully considered their concerns. Based on the suggestion of the Reviewer, we have decided to remove the kSTORM data from this manuscript and will continue to validate this method for use in future experiments.

Because we have removed the kSTORM data, we have not provided point-by-point responses to the Reviewer's comments below since they all apply to data now removed from the manuscript. However, we thank the Reviewer for the detailed questions about the method, and we will be following up on these important questions in subsequent measurements to fully validate the method. In the future, we hope the Reviewer will be convinced there is useful information in these images!

1. The main problem I have is that the authors claim to estimate binding times from only on average three counts. For a single exponential decay, the standard deviation of the measurement is equal to its mean, for a double-exponential decay, tens to hundreds of measurements are required to estimate the two time constants.

2. From the previous experiments, the authors have estimates on tau1 and tau2, from those they could estimate the standard deviation of the distribution, and taking each count as a measurement they could plot the SEM of the kSTORM rendering as std/sqrt(counts). The counts need to be mentioned in the figure legend, I strongly recommend adding this SEM image to Figure 7.

3. I do not understand the difference between converged and average images. Please specify in S8 precisely what these images mean. I would rather add the SEM figure as suggested above and maybe show repeated images of the same structures.

4. If there is any information in the kSTORM images, then a repeated measurement should look somewhat similar. I suggested previously plotting average tau values pixelwise from one measurement vs a second measurement (or one half vs the second half). Even if the data are noisy (as expected for the small counts and if only half the data is used each time), there must be a correlation. The difference between the two measurements is an estimate for the statistical error. I would like to see this plot, even if the noise is large. There is no fundamental limitation in the length of kSTORM experiments, fluorophores are not depleted, thus taking a second measurements at the same position should be entirely feasible.

5. Although the sigma_time vs sigma_position analysis is interesting, it provides only some indication. For example, any variation of excitation intensity across the FoV can increase the variance in space. Also a statistically analysis with sometimes only 1 or 2 data points (as is common for on average 3 counts) needs to be performed properly beyond calculating the

standard deviation, and much more information needs to be provided that the this comparison is meaningful.

6. As long as the values of the image have no concrete meaning, a cluster analysis seems pointless. The analysis procedure seems somewhat arbitrary. Also, the Omega analysis is not a common analysis, and it is not clear what secondary parameters (e.g. the average time constant, number of localizations,…) the Omega value could depend on. Thus, I am not convinced that the difference in Omega indeed indicates differences (or even existence) of spatial variations of the tau.

7. A minor point: Usually "STORM" is interpreted as single-molecule localization using organic dyes that enter a dark state, usually due to presence of a blinking buffer. The authors use "PAINT" and could consider adapting the acronym and citing previous literature that uses such transient binding to image structures, including actin.
